# Mechanochemical synthesis of aromatic ketones: pyrylium tetrafluoroborate mediated deaminative arylation of amides†

**DOI:** 10.1039/d4sc00904e

**Published:** 2024-05-14

**Authors:** Satenik Mkrtchyan, Oleksandr Shalimov, Michael G. Garcia, Jiří Zapletal, Viktor O. Iaroshenko

**Affiliations:** a Department of Chemistry, Faculty of Natural Sciences, Matej Bel University Tajovského 40 97401 Banska Bystrica Slovakia iva108@googlemail.com viktor.iaroshenko@umb.sk Iaroshenko.V@gust.edu.kw; b Department of Heteroatom Chemistry, Institute of Organic Chemistry, National Academy of Sciences of Ukraine 5 Murmans'ka 02660 Kyiv Ukraine; c Department of Biology/Chemistry, Center for Cellular Nanoanalytics (CellNanOs), Universität Osnabrück Barbarastr. 7 D-49076 Osnabrück Germany; d Division of Wood Chemistry and Pulp Technology, Department of Fiber and Polymer Technology, School of Chemistry, Biotechnology and Health, KTH Royal Institute of Technology Teknikringen 56-58 SE-100 44 Stockholm Sweden; e Functional Materials Group, Gulf University for Science and Technology Mubarak Al-Abdullah 32093 Kuwait; f Centre of Research Impact and Outcome, Chitkara University Institute of Engineering and Technology, Chitkara University Rajpura 140401 Punjab India

## Abstract

A new method has been introduced that is able to tackle the complexities of N–C(O) activation in amide moieties through utilization of pyrylium tetrafluoroborate in a mechanochemical setting, where amide bonds undergo activation and subsequent conversion to biaryl ketones. Due to the employment of a mechanochemical setting, the reaction conforms to green chemistry principles, offering an environmentally friendly approach to traditional amide derivatization techniques that rely on transition metals to achieve further functionalization.

## Abstract 2

A new method has been introduced that is able to tackle the complexities of N–C(O) activation in amide moieties through utilization of pyrylium tetrafluoroborate in a mechanochemical setting, where amide bonds undergo activation.

## Graphical abstract

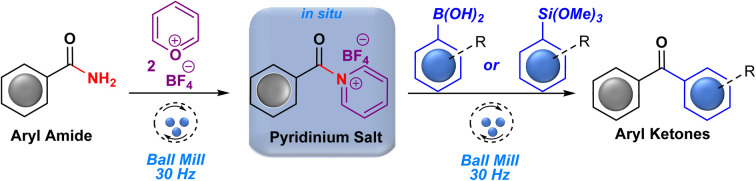

## Introduction

Due to their ubiquity in natural compounds and their role as buildings blocks for transformation into specific functional groups, conversion of the C–N bond in amide groups, R–CO(sp^2^)–NH_2_, is of great significance to organic chemistry^1^ as it permits modification of molecular properties, such as polarity, solubility, and reactivity,^2,3^ thereby enabling the optimization of the biological activity and pharmacokinetic profiles of target compounds.^4,5^ Despite significant advancements in amide functionalization, notable obstacles arise from the bond's inertness and its poor leaving group nature, making their modification a difficult endeavour.^6,7^ In research pioneered by Katritzky, it was determined that through complexation with pyrylium salts, amines can be converted into pyridinium groups that are good leaving groups. With this discovery, the traditional view on non-reactive C–N bonds was altered to that of potential electrophiles capable of undergoing further modification.^8^ Building on this line of work, Cornella *et al.* reported selective S_N_Ar functionalization of aminoheterocycles *via* utilization of pyrylium tetrafluoroborate.^9–12^ Partly inspired by their findings, we developed a new, green, mechanochemical procedure that applies a mechanochemical protocol established by Hajime Ito's group^13^ and combines it with the established work on pyrylium tetrafluoroborates to develop a unique method that allows for the activation and subsequent functionalization of amides. With our method, we previously managed to convert aromatic amines into aryl trifluoromethyl ethers, which produced high yields and excellent selectivity under liquid assisted conditions.^14^ For this work, we continue to broaden the scope of our approach towards the conversion of aryl amides into biaryl ketones.

As previously mentioned, the C–N bond in amides is generally considered unreactive towards substitution reactions, making functionalization of amides a complicated synthetic task. This is due to their ability to participate in resonance, leading to partial double bond character between the carbon and nitrogen atoms.^6,15^ In addition, the conversion of primary amides CO(sp^2^)–NH_2_ into functional derivatives is further complicated due to their low heterolytic nucleofugality (C_6_H_5_–CO–NH_2_, BDE of 96.4 kcal mol^−1^).^16^ In recent years, there has been progress in the development of alternative strategies on deaminative functionalization of amides. This has allowed further functionalization of amides into a variety of functional groups.^17,18^

Since amides are inert and stable, functionalization *via* substitution can be difficult if conducted through polar processes. Common approaches to tackle the inert nature of amides are through pre-functionalization, followed by derivatization *via* a substitution reaction to achieve targeted functionalization, which can often turn into a time-consuming endeavour.^6^ Other approaches towards derivatization may require transformation into different functional groups *via* diazotization^19^ or polyalkylation.^20,21^

**Scheme 1:**
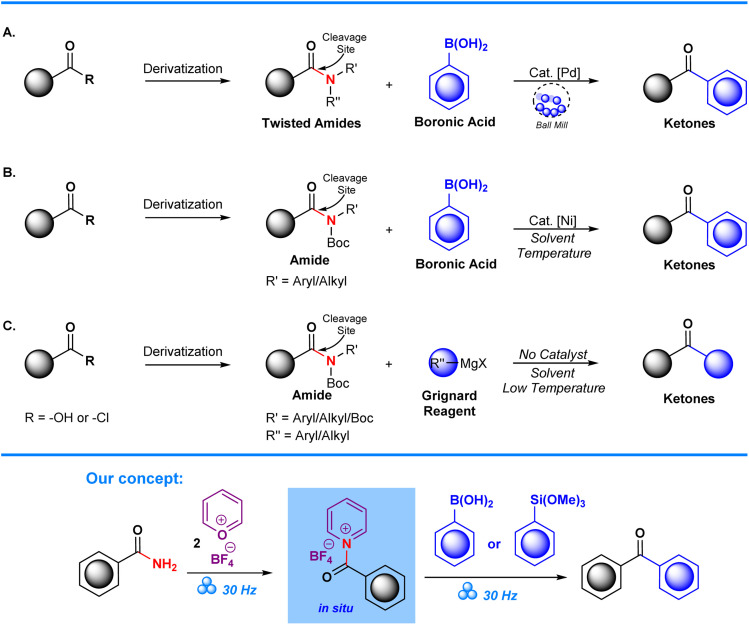
Sample reported strategies for amide bond functionalization and our concept.

Transformation of substituted amides into ketonic derivatives has been achieved through the employment of Suzuki–Miyaura cross-couplings on substituted amides as demonstrated by the Szostak group. In their work, chemoselective C–N activation is achieved through utilization of transition metals, in this case Pd, which facilitates cross-coupling of aryl boronic acids to synthesize aryl ketone derivatives (Scheme 1A).^22–25^ Garg *et al.* also demonstrated successful conversion of N-Boc and alkyl/aryl amides into ketones through Ni-catalyzed Suzuki–Miyaura cross-coupling. Despite their success, a significant limitation in this approach is the need to employ amide derivatives, such as N-Boc amides,^26,27^ and other forms of twisted amides (Scheme 1B), which places further emphasis on the starting material's pre-functionalization. Additionally, the use of transition metal catalysts can be in itself detrimental due to their potential toxicity, high cost, and environmental impact.^28,29^ These factors can potentially lead to several issues with regards to low yields, increased reaction times, and the need for extra reagents and purification steps. Subsequent studies have tried to circumvent the issues posed by utilization of metal catalysts *via* Grignard modification of aryl ketones from N-Boc substituted amides. However, despite the improvement with regards to absence of a metal catalyst, complications arise due to the exothermic nature of the Grignard reaction which requires that reactions are performed in a low-temperature environment. This limitation reduces the attractiveness of the method for green chemistry (Scheme 1C).^30^

Deamination of amides is possible through utilization of pyrylium salts.^9,14,31^ As already established, Katritzky *et al.* managed to activate primary amines, C(sp^3^)–NH_2_, *via* formation of pyridinium salts. This led to a more systematic approach in the development of novel, synthetic pathways that enable the transformation of primary amines into reactive pyridinium salts.^8,32^ Further research demonstrated that pyridinium salts are able to undergo coupling with boronic acids, thereby allowing for the incorporation of diverse groups into their molecular framework.^33–35^ Consequently, several functionalities were introduced *via* this versatile approach, thus broadening the scope of compounds with potential applications in medicinal chemistry.^36^

This study's main aim is to continue the exploration of the capabilities of pyrylium tetrafluoroborate within a mechanochemical setting by deamination and arylation of aryl amide substrates, aryl–CO(sp^2^)–NH_2_. In doing so, we aim to develop an optimum mechanochemical protocol, and continue to explore the substrate scope. Additionally, we include the production of arylated ketones with fluorine substituents into our product scope as they are highly sought after due to their unique physical, chemical, and biological properties.^37^ The presence of fluorine is known to influence the reactivity, stability, and bioavailability of organic molecules, making them relevant compounds for pharmaceutical research.^38,39^

**Scheme 2:**
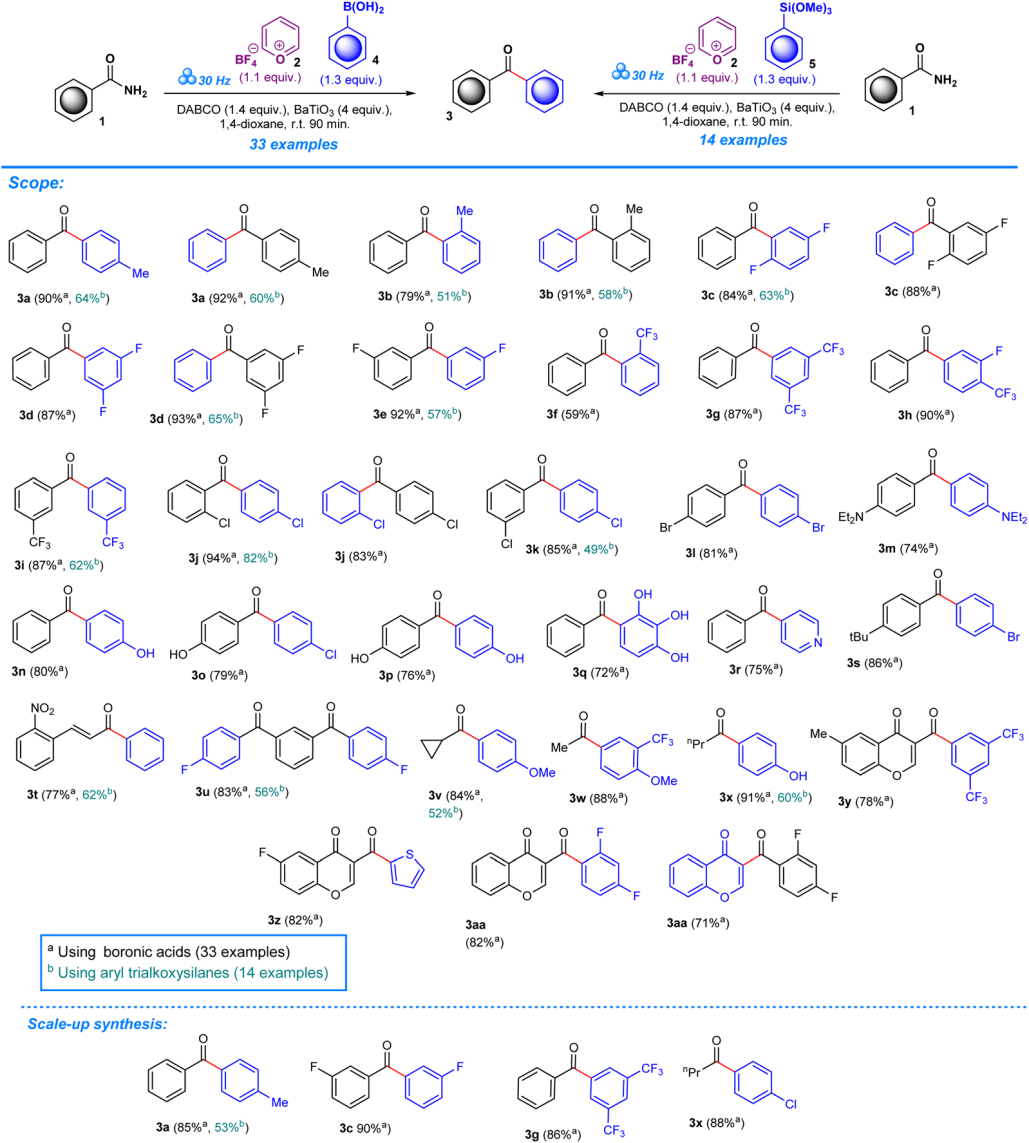
Scope of aromatic ketones.

The use of pyrylium tetrafluoroborate in a mechanochemical deaminative arylation is a promising route for efficiently synthesizing fluorinated aryl molecules.^9,14,40^ A mechanochemical setting has distinct advantages over conventional methodologies in contrast to standard approaches that involve pre-functionalization of amides (Scheme 2).^27,41^ Fewer synthetic steps, lower reaction times and diminished resource consumption are achieved by avoidance of amine pre-modification, which in turn leads to less production of chemical waste. In this synthetic approach, ball milling is employed as a solvent-less mechanochemically driven process that optimizes reaction conditions through mechanical stimuli of a piezoelectric barium titanate, BaTiO_3_. Through this approach we ensure that product formation is performed in an environmentally favorable and sustainable way.^13,42–44^ Overall, the application of pyrylium tetrafluoroborate in a mechanochemical setting is a noteworthy development in the field, providing a productive, long-lasting, and environmentally benign substitute for conventional amine derivatization techniques.

## Results and discussion

The deamination of aryl amines builds upon previous research work which focused on the introduction of trifluoromethoxy moieties to aromatic amines.^14^ The scope of this methodology is aimed at further broadening its versatility by including the deamination of aryl amides in order to produce ketonic arylated compounds.

**Scheme 3:**
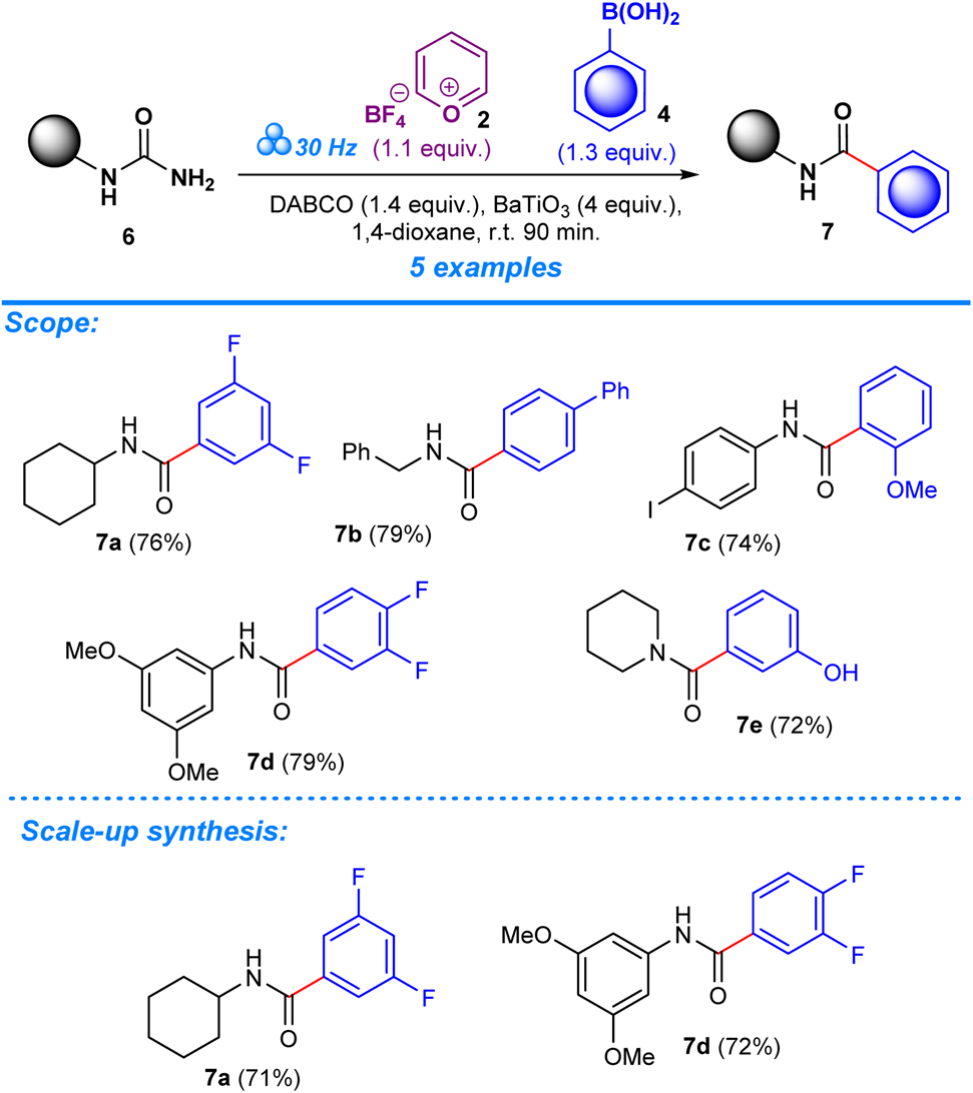
Scope of aromatic amides.

As in our previous work, experiments were performed in a one-pot mechanochemical setting, for which a formulaic approach was developed to test for a variety of reagents until optimum conditions were achieved. To create a general formulaic protocol from the given series of reactions, we took into consideration the variable parts in each reaction and standardized common components. Each reaction consistently employed 1 equivalent of aryl amides (1) (Scheme 2) or ureas (6) (Scheme 3), 1.1 equivalents of pyrylium tetrafluoroborate 2, and 1.3 equivalents of boronic acids (4) or trialkoxysilanes (5). In all reactions, 4 equivalents of BaTiO_3_ are consistently used. Control experiments were designed to assess the importance of BaTiO_3_ and other additives in the reaction outcome. In the first experimental control, standard quantities of 1 equivalent of aryl amides, or ureas, 1.1 equivalents of pyrylium tetrafluoroborate, and 1.3 equivalents of boronic acids, or trialkoxysilanes, were tested for product formation, while omitting BaTiO_3_ and any additives (Tables S1 and S2, entry 1). The second control included all the standard components in the same quantities as described above, and BaTiO_3_, but no additive was added, which helped reveal the significance of additives to the success of the reaction (Tables S1 and S2, entry 2).

The addition of varying quantities of distinct bases or additives is the variable component across these reactions. These include Na_2_CO_3_, K_2_CO_3_, Cs_2_CO_3_, NEt_3_, NEt_2_Ph, (^i^Pr)_2_NEt, quinuclidine, and DABCO (1,4-diazabicyclo[2.2.2]octane), with their quantities ranging from 1.4 to 1.8 equivalents. Systematic analysis of the impact of different additives and their quantities on the success of target product formation and yield efficiency was allowed by this approach. All the reactions were conducted at a consistent milling frequency of 30 Hz (ref. 45) for 90 minutes, with the addition of 0.25 mL of 1,4-dioxane as a liquid-assisted grinding (LAG) additive.^46^

In the set of reactions involving boronic acid as a coupling agent (Table S1†) varying yields are displayed based on the type of additive present in the reaction. For the first reactions up to NEt_3_ (Table S1,† entries 1–6), the yield was 0%. The reaction did not proceed effectively under these conditions – milling frequency and duration, along with the reactant ratios. However, upon introducing different additives in subsequent reactions, a different result was obtained. For example, the reaction involving NEt_2_Ph showed a yield of 58%. Furthermore, additives like (^i^Pr)_2_NEt, quinuclidine, and DABCO produced yields of 63%, 90%, and 90%, respectively (Table S1, entries 7–10). A similar pattern was observed for reactions employing trialkoxysilane as a coupling reagent, with product formation occurring in the presence of hindered NEt_2_Ph, (^i^Pr)_2_NEt, quinuclidine, and DABCO, although the yields were comparatively lower than in reactions employing boronic acids as a coupling agent (Table S2,† entries 1–6). For instance, the yield obtained for NEt_2_Ph was recorded at 41%, whereas the compounds (^i^Pr)_2_NEt, quinuclidine, and DABCO were observed to have yields of 12%, 52%, and 64% (Table S2, entries 7–10).

We established a sequence in our additives to discern patterns in our data. Initially, we have the carbonates (Na_2_CO_3_, K_2_CO_3_, Cs_2_CO_3_), which are classified as mild bases.^47^ Then, we introduced stronger bases and/or nucleophilic catalysts: NEt_3_, NEt_2_Ph, and (^i^Pr)_2_NEt. Finally, the trend culminated with structurally complex and highly nucleophilic enhancing compounds like quinuclidine and DABCO.^48^

Of note, the reactions were able to proceed outside of a glovebox. However, an issue was consistently encountered with respect to the reproducibility of our results, which produced lower yields. The problem was attributed to the influence of ambient moisture which likely resulted in partial hydrolysis of the *in situ* formed pyridinium salts. As a result, experiments were conducted within a glovebox setting in order to ensure reproducibility of our results.

The study was expanded to examine whether different solvents could affect the synthesis of our target molecule to assess the viability of our approach in a variety of solvent systems. In line with previous studies, it was found that the production of the target compound was not possible under solvent conditions.^14^ The experimental setups were methodically varied by incorporating a different solvent system: 1 equivalent of aryl amides, 1.1 equivalents of 2, 1.3 equivalents of (4 and 5), 1.4 equivalents of DABCO and 4 equivalents of BaTiO_3_, which produced the optimum reaction conditions for our solid-phase reactions. This mixture was then subjected to reflux in a range of solvents including methanol (MeOH), acetonitrile (CH_3_CN), 1,4-dioxane, and dimethylformamide (DMF), with the DMF reactions being conducted at two different temperatures: 100 °C and 130 °C (Tables S1 and S2†). The reactions consistently produced a yield of 0% with regards to our target compound in the selected solvent systems in contrast to the results obtained under mechanochemical conditions, indicating that the piezoelectric properties of barium titanate (BaTiO_3_) were crucial to the method's success, particularly when combined with DABCO. Activation of barium titanate requires mechanical force to deform its structure, resulting in the formation of transitory, polarized particles. Because of this, an element of probability or randomness is introduced to the activation process, as the mechanical stress applied to BaTiO_3_ depends on the size, frequency, and quantity of the ball mills employed.^13,49^ A typical solvent system fails to provide the mechanical force required to alter the structure of BaTiO_3_, emphasizing the importance of a mechanochemical strategy for effective coupling processes. The inclusion of BaTiO_3_ may help overcome the reaction's activation energy barrier.^50^

After establishing the optimum conditions to functionalize aryl amides, the viability of the method was assessed by testing different substrates with substituents at the -*ortho*, -*meta*, and -*para* positions. Target products (3a–aa) achieved between 59% and 94% isolated yield for reactions carried out in the presence of boronic acid. Product formation was similarly accomplished for reactions that employed trialkoxysilane as a coupling agent (3a–e, 3i–j, 3k, 3t–v, 3x), albeit with slightly lower efficiency based on the yields which ranged from 49% to 82%. The scope was also expanded to include the deamination of amines belonging to the urea functional group, applying the same conditions with regards to milling frequency, duration, reactant ratios, and additives (Scheme 3). Mirroring the success of products from the aryl amide series, formation from urea substrates resulted in favorable yields in which a yield of 76% was obtained for 1-cyclohexylurea, 7a, with a similar consistency observed for products 7b–e, where yields ranged from 72% to 79%. Gram-scale reactions were performed to assess the scalability of the method to determine its potential in a large-scale, industrial setting. For the aryl amide series, the reactions resulted in favorable yield, although being comparatively lower to the yields obtained for reactions performed in the smaller ball mill setting: 3a (85%, 53%), 3c (90%), 3g (86%), and 3x (88%) (Scheme 2). Similarly, products obtained through reactions employing urea substrates produced the following results: 7a (71%) and 7d (72%) (Scheme 3). In light of the data provided by our results and established literature on the subject of ball milling,^51^ it becomes clear that the equipment of choice significantly influenced our mechanochemical reaction settings. Our investigations, which compared yields from reactions in a 5 mL grinding vessel with three 5 mm diameter balls to those in a 25 mL vessel with four 10 mm diameter balls, demonstrate this fact. Consistently higher outputs were produced by the smaller ball mill, most likely owing to differences in mechanical energy inputs and collision dynamics in these two configurations. It is likely that in the smaller ball mill setup, the concentrated and effective distribution of mechanical energy, due to the confined space and the lower ball size and number, enhances the piezoelectric effect of barium titanate, resulting in higher frequencies and intensities of collisions that lead to better polarization and higher electrical energy input by the piezoelectric material, whereas in the 25 mL tank mechanical energy dispersion is greater than in the smaller tank. As a result, fewer and less powerful collisions lead to lower mechanical stress on the piezoelectric barium titanate, which would activate the reactants less effectively.^13,49^

**Scheme 4:**
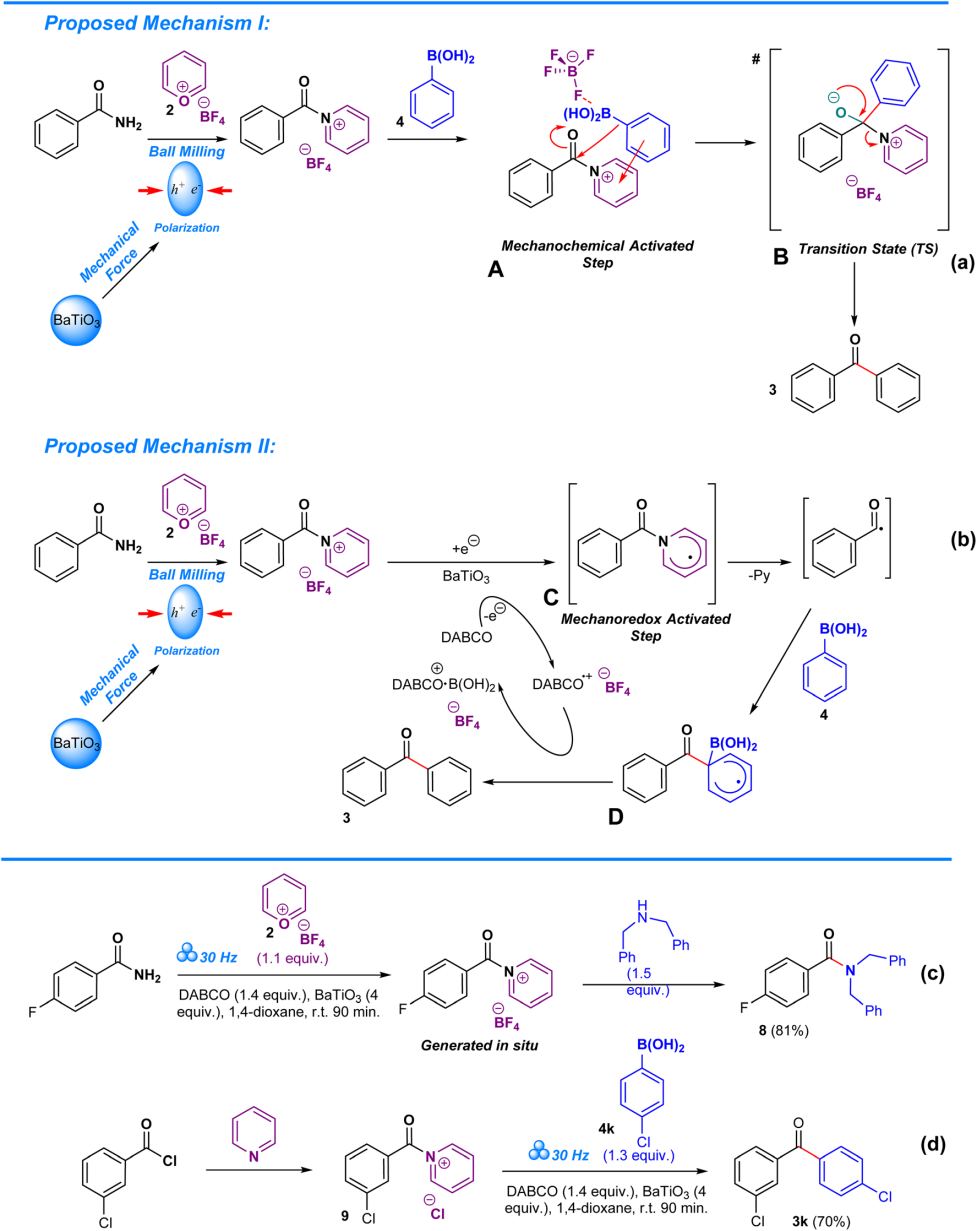
Mechanism of deaminative arylation of aryl amides.

Deamination in aryl amides is driven *via* a mechanochemically transition-metal-free C–C coupling of boronic acids (or trialkoxysilanes). Two proposals were devised that explain the role of pyrylium tetrafluoroborate and barium titanate polarization in the deaminative arylation process. In the first step of proposed mechanism I (Scheme 4a), barium titanate undergoes polarization when subjected to mechanical stress during ball milling^13,44,50^ while amide undergoes *in situ* complexation with pyrylium tetrafluoroborate to form a pyridinium.^8,14,40^ Due to the energy input by barium titanate, the pyridinium intermediate becomes excited, becoming a channel for the arylation of carbonyl in the absence of a transition metal, thereby enabling a unique reaction pathway that differs from typical solvothermal conditions.^13^ Consequently, the proposed mechanochemically driven step A proceeds towards nucleophilic addition, in which boronic acid attacks the carbonyl moiety, resulting in the formation of the transition state B. As a result, the aryl group from the boronic acid becomes covalently bonded to the carbonyl carbon, leading to the formation of the target ketonic aryl upon elimination of the pyridine moiety. Structure B is crucial to this process as it allows for direct arylation of the carbonyl, a process that is challenging in the absence of a transition metal catalyst.^35^ In the case of proposed alternative mechanism II (Scheme 4b), barium titanate undergoes polarization when subjected to mechanical stress during ball milling^13,44,50^ while amide undergoes *in situ* complexation with pyrylium tetrafluoroborate to form a pyridinium.^8,14,40^ Polarization of barium titanate facilitates monoradical electron transfer from DABCO to the pyridinium intermediate. This mechanoredox activated step C is crucial towards achieving deamination of the amide and allowing formation of a channel for carbonyl arylation in the absence of a transition metal, thus also enabling a unique reaction pathway that differs from typical solvothermal conditions.^13^ For this case, the deamination of the aryl amide leads towards a radical intermediate, which upon coupling with the boronic acid results in the formation of transition state D, allowing for direct coupling of an aryl group to the carbonyl without a transition metal catalyst. The electron transfer from the transition state D to DABCO is vital as it ensures a continuous redox cycle through enabling DABCO to alternate between reduced and oxidized states.^13,35^ In both proposals, the driving force of the process is the energy released by the polarization of barium titanate.

To prove the involvement of the pyridinium salts as intermediates in this methodology we conducted two reactions which are depicted in Schemes 4c and d. The two-step, one-pot consecutive reaction of 4-fluorobenzamide with pyrylium tetrafluoroborate 2 under the standard reaction conditions meant the formation of the pyridinium salt at the first step, followed by the addition of dibenzylamine, resulting in the formation of the corresponding amide 8. Analogously, the reaction of freshly prepared pyridinium salt 9 with boronic acid 4k delivered the ketone 3k. Cornella's work partially inspired us to develop this approach towards amides *via* his work on functionalization of sulfonyl chlorides and sulfonamides.^10,12^ In particular, his work on hydroxylation of aminoheterocycles and electron deficient anilines provides us with an in-depth view on the mechanistic complexities arising from pyrylium tetrafluoroborate mediated hydroxylation of the –NH_2_ group in heteroaromatic compounds through a Lossen-type rearrangement.^52^ Despite the veracity of their mechanistic proposal, our approach remains consistently different as it requires solid-state conditions to allow reaction completion and utilizes amides as the starting material, in contrast to Cornella's solution-based approach which targets sulfonamides, alluding to the possibility of distinct mechanistic pathways that are not as typically expected.

## Conclusion

Mechanochemical deaminative arylation *via* utilization of pyrylium tetrafluoroborate represents a step forward for the further development of green methods in synthetic organic chemistry by addressing the challenges associated with the activation of the CO(sp^2^)–NH_2_ bond in primary amine activation within amide moieties. Direct functionalization of amides is an important synthetic method because of their critical role as building blocks and ubiquity in biological molecules, making them important structures due to their ability to modify molecular properties for optimal biological activity and pharmacokinetics. In addition to improvements being made to optimize the reaction process, scalability was investigated through gram-scale reactions, which yielded promising results for industrial applications; furthermore, the high yields obtained for both aryl amides and ureas underscore the potential of our method to be adopted for the mass production of complex molecules, crucial for the pharmaceutical industry. Therefore, in this research the potential of pyrylium tetrafluoroborate in a mechanochemical setting was established as a solid synthetic alternative for the creation of a wide range of important substituted products.

## Data availability

The datasets supporting this article have been uploaded as part of the ESI.†

## Author contributions

Conceptualization: S. M. and V. O. I.; methodology: S. M., V. O. I. and O. S.; investigation: S. M., O. S., M. G. G., J. Z., and V. O. I.; writing – original draft: M. G. G., S. M. and V. O I.; writing – review & editing: M. G. G., S. M. and V. O. I.; funding acquisition: V. O. I.; resources: V. O. I.; supervision: V. O. I.

## Conflicts of interest

The authors declare no competing financial interests.

## Supplementary Material

SC-015-D4SC00904E-s001
