# Supplementary material for: Mechanochemical synthesis of aromatic ketones: pyrylium tetrafluoroborate mediated deaminative arylation of amides
Source: Chem Sci. 2024 May 14;15(24):9155–63. doi: 10.1039/d4sc00904e (PMC11186303; doi:10.1039/d4sc00904e)
Supplement: SC-015-D4SC00904E-s001 [file SC-015-D4SC00904E-s001.pdf]

Electronic Supplementary Material (ESI) for Chemical Science.
This journal is © The Royal Society of Chemistry 2024

Supporting Information

Mechanochemical Synthesis of Aromatic Ketones: Pyrylium Tetrafluoroborate Mediated Deaminative Arylation of Amides.

Satenik Mkrtchyan,[a]* Oleksandr Shalimov,[b] Michael G. Garcia,[c] Jiří Zapletal,[a] Viktor O. Iaroshenko.[a,d,e,f]*

[a]Department of Chemistry, Faculty of Natural Sciences, Matej Bel University, Tajovského 40, 97401, Banska Bystrica, Slovakia.
[b]Department of Heteroatom Chemistry, Institute of Organic Chemistry, National Academy of Sciences of Ukraine, 5 Murmans'ka, 02660 Kyiv, Ukraine.
[c]Department of Biology/Chemistry, Center for Cellular Nanoanalytics (CellNanOs), Universität Osnabrück, Barbarastr. 7, D-49076 Osnabrück, Germany.
[d]Division of Wood Chemistry and Pulp Technology, Department of Fiber and Polymer Technology, School of Chemistry, Biotechnology and Health, KTH Royal Institute of Technology, Teknikringen 56-58, SE-100 44 Stockholm, Sweden.
[e]Functional Materials Group, Gulf University for Science and Technology, Mubarak Al-Abdullah 32093, Kuwait, Kuwait.
[f]Centre of Research Impact and Outcome, Chitkara University Institute of Engineering and Technology, Chitkara University, Rajpura-140401, Punjab, India.

Table of Contents

(A) Experimental Section S3

(A-1) Scope of reagents used S3

(A-2) Reaction conditions screening S5

(B) Characterization of products S9

(C) Copies ^{1}H and ^{13}C NMR spectra S28

(A) Experimental section.

Commercially available starting materials, reagents, catalysts, anhydrous and degassed solvents were used without further purification. Flash column chromatography was performed with Merck Silica gel 60 (230-400 mesh). The solvents for column chromatography were distilled before the use. Thin layer chromatography was carried out using Merck TLC Silica gel 60 F_{254} and visualized by short-wavelength ultraviolet light or by treatment with potassium permanganate ($KMnO_4$) stain. ^{1}H, ^{13}C and ^{19}F NMR spectra were recorded on a Bruker 250, 400 and 500 MHz at 20ºC. All ^{1}H NMR spectra are reported in parts per million (ppm) downfield of TMS and were measured relative to the signals for $CHCl_3$ (7.26 ppm) and DMSO (2.50 ppm). All $^{13}C\{^{1}H\}$ NMR spectra were reported in ppm relative to residual $CHCl_3$ (77.00 ppm) or DMSO (39.70 ppm) and were obtained with ^{1}H decoupling. Coupling constants, *J*, are reported in Hertz (Hz). Gas chromatographic analyses was performed on Gas Chromatograph Mass Spectrometer GCMS-QP2010 Ultra instrument. Mechanochemical synthesis was performed using the Retsch MM400 mill using the standard kit. Liquid chemicals were dosed using gas tight micro syringes. Isolation of obtained compounds was achieved by column chromatography on Silica gel. All commercially available compounds were purchased from appropriate vendors. The pyrylium tetrafluoroborate is commercially available, we prepared it following previously published literature:

1. Moser, D.; Duan, Y.; Wang, F.; Ma, Y.; O'Neill, M. J.; Cornella, J. *Angew. Chem. Int. Ed.* **2018**, *57*, 11035–11039.
2. Gómez-Palomino, A.; Ghiazza, C.; Busch, J.; Wagner, L.; Cornella. J. *Org. Synth.* **2023**, *100*, 361-381.

A-1. Scope of reagents used.

Scheme S1. List of amides **1** used.

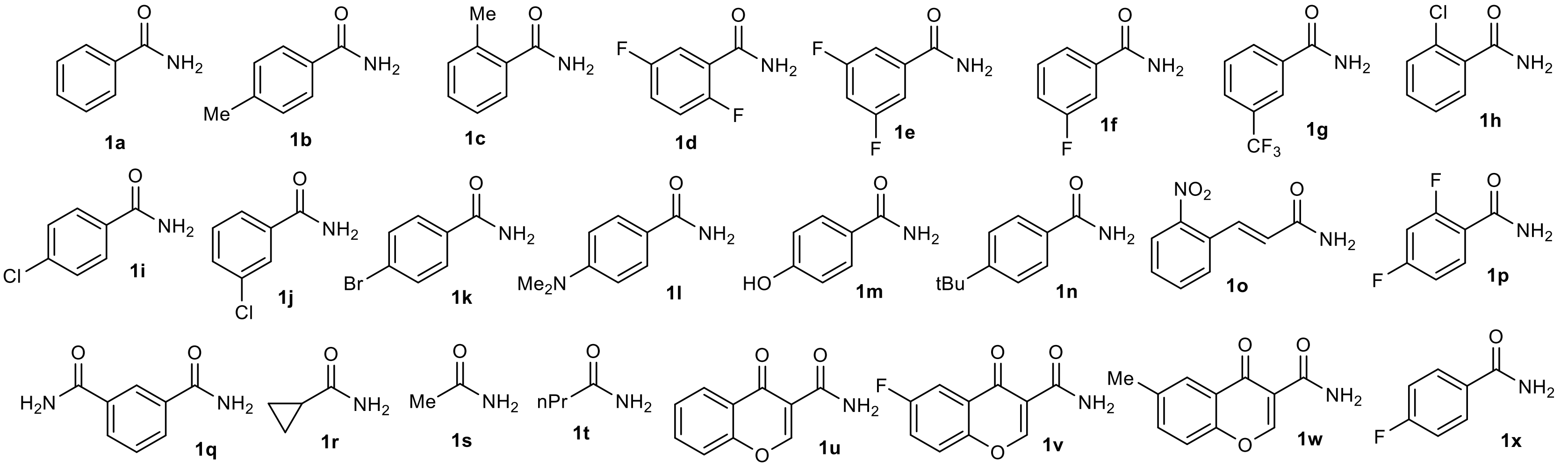

Scheme S2. List of aryl boronic acids **4** used.

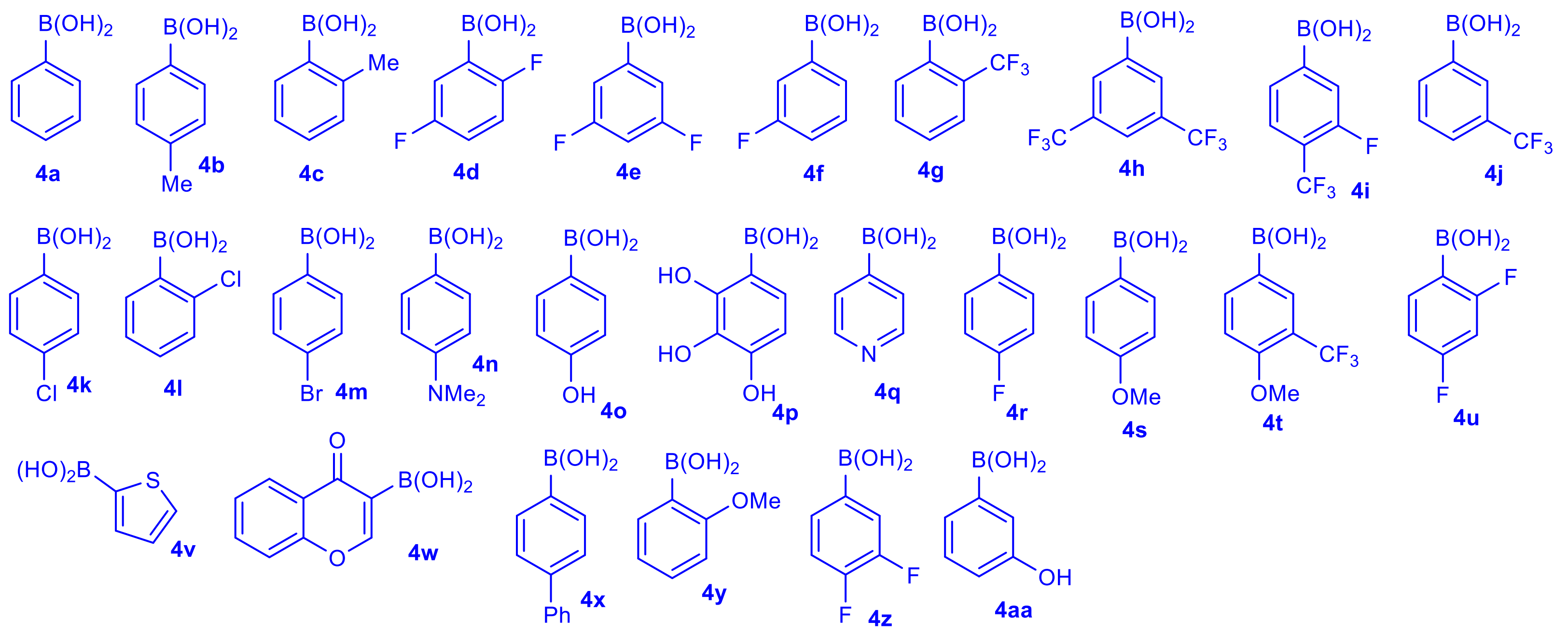

Scheme S3. List of aryl trialkoxysilanes **5** used.

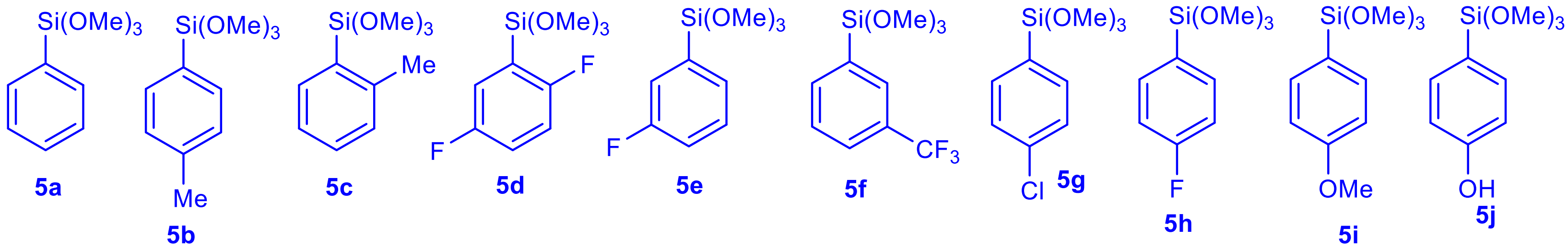

Scheme S4. List of urea **6** used.

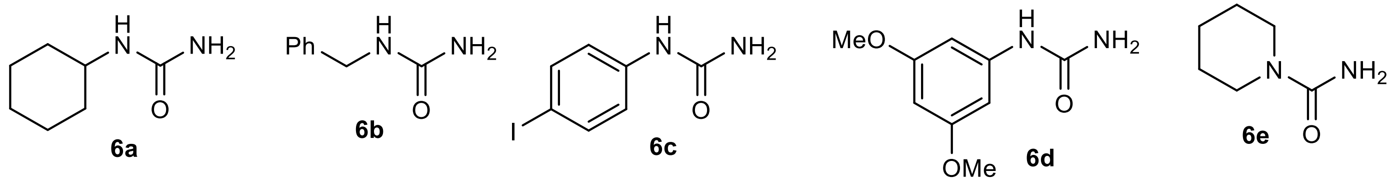

A-2. Reaction conditions screening.

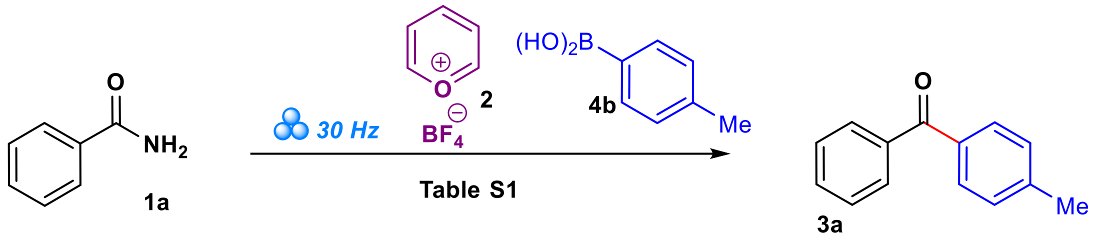

Table S1			
**Entry**	**Reaction components**	**Milling frequency/time**	**Yield (%) 3a**
	**Reactions in solid phase**		
1	**1a** (1 equiv.), **2** (1.1 equiv.), **4b** (1.3 equiv.)	30Hz/90 min	0
2	**1a** (1 equiv.), **2** (1.1 equiv.), **4b** (1.3 equiv.), $BaTiO_3$ (4 equiv.)	30Hz/90 min	0
3	**1a** (1 equiv.), **2** (1.1 equiv.), **4b** (1.3 equiv.), Na_2CO_3 (1.4 equiv.), $BaTiO_3$ (4 equiv.)	30Hz/90 min	0
4	**1a** (1 equiv.), **2** (1.1 equiv.), **4b** (1.3 equiv.), K_2CO_3 (1.4 equiv.), $BaTiO_3$ (4 equiv.)	30Hz/90 min	0
5	**1a** (1 equiv.), **2** (1.1 equiv.), **4b** (1.3 equiv.), Cs_2CO_3 (1.4 equiv.), $BaTiO_3$ (4 equiv.)	30Hz/90 min	0
6	**1a** (1 equiv.), **2** (1.1 equiv.), **4b** (1.3 equiv.), NEt_3 (1.4 equiv.), $BaTiO_3$ (4 equiv.)	30Hz/90 min	0
7	**1a** (1 equiv.), **2** (1.1 equiv.), **4b** (1.3 equiv.), NEt_2Ph (1.4 equiv.), $BaTiO_3$ (4 equiv.)	30Hz/90 min	58
8	**1a** (1 equiv.), **2** (1.1 equiv.), **4b** (1.3 equiv.), $(^iPr)_2NEt$ (1.4 equiv.), $BaTiO_3$ (4 equiv.)	30Hz/90 min	63
9	**1a** (1 equiv.), **2** (1.1 equiv.), **4b** (1.3 equiv.), Quinuclidine (1.8 equiv.), $BaTiO_3$ (4 equiv.)	30Hz/90 min	90
**10**	**1a (1 equiv.), 2 (1.1 equiv.), 4b (1.3 equiv.), DABCO (1.4 equiv.), $BaTiO_3$ (4 equiv.)**	**30Hz/90 min**	**90**
	**Reactions in solution**		
11	**1a** (1 equiv.), **2** (1.1 equiv.), **4b** (1.3 equiv.), DABCO (1.4 equiv.), $BaTiO_3$ (4 equiv.), MeOH, reflux	------/24 h	0
12	**1a** (1 equiv.), **2** (1.1 equiv.), **4b** (1.3 equiv.), DABCO (1.4 equiv.), $BaTiO_3$ (4 equiv.), CH_3CN, reflux	------/24 h	0
13	**1a** (1 equiv.), **2** (1.1 equiv.), **4b** (1.3 equiv.), DABCO (1.4 equiv.), $BaTiO_3$ (4 equiv.), 1,4-dioxane, reflux	------/24 h	0
14	**1a** (1 equiv.), **2** (1.1 equiv.), **4b** (1.3 equiv.), DABCO (1.4 equiv.), $BaTiO_3$ (4 equiv.), CH_3CN, reflux	------/24 h	0
15	**1a** (1 equiv.), **2** (1.1 equiv.), **4b** (1.3 equiv.), DABCO (1.4 equiv.), $BaTiO_3$ (4 equiv.), DMF, 100 °C	------/24 h	0
16	**1a** (1 equiv.), **2** (1.1 equiv.), **4b** (1.3 equiv.), DABCO (1.4 equiv.), $BaTiO_3$ (4 equiv.), DMF, 130 °C	------/24 h	0

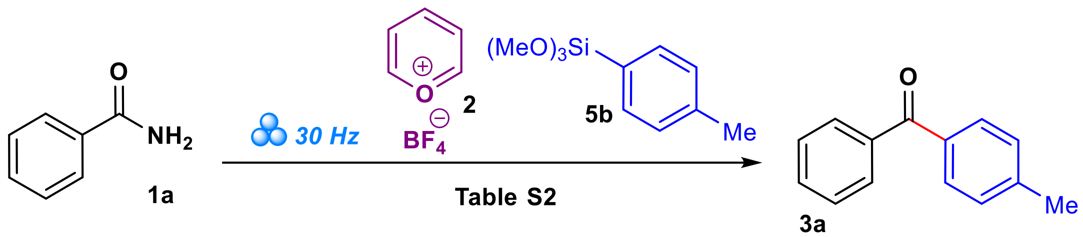

Table S2			
Entry	Reaction components	Milling frequency/time	Yield (%) 3a
	Reactions in solid phase		
1	**1a** (1 equiv.), **2** (1.1 equiv.), **5b** (1.3 equiv.)	30Hz/90 min	0
2	**1a** (1 equiv.), **2** (1.1 equiv.), **5b** (1.3 equiv.), $BaTiO_3$ (4 equiv.)	30Hz/90 min	0
3	**1a** (1 equiv.), **2** (1.1 equiv.), **5b** (1.3 equiv.), Na_2CO_3 (1.4 equiv.), $BaTiO_3$ (4 equiv.)	30Hz/90 min	0
4	**1a** (1 equiv.), **2** (1.1 equiv.), **5b** (1.3 equiv.), K_2CO_3 (1.4 equiv.), $BaTiO_3$ (4 equiv.)	30Hz/90 min	0
5	**1a** (1 equiv.), **2** (1.1 equiv.), **5b** (1.3 equiv.), Cs_2CO_3 (1.4 equiv.), $BaTiO_3$ (4 equiv.)	30Hz/90 min	0
6	**1a** (1 equiv.), **2** (1.1 equiv.), **5b** (1.3 equiv.), NEt_3 (1.4 equiv.), $BaTiO_3$ (4 equiv.)	30Hz/90 min	0
7	**1a** (1 equiv.), **2** (1.1 equiv.), **5b** (1.3 equiv.), NEt_2Ph (1.4 equiv.), $BaTiO_3$ (4 equiv.)	30Hz/90 min	41
8	**1a** (1 equiv.), **2** (1.1 equiv.), **5a** (1.3 equiv.), $(^{i}Pr)_2NEt$ (1.4 equiv.), $BaTiO_3$ (4 equiv.)	30Hz/90 min	12
9	**1a** (1 equiv.), **2** (1.1 equiv.), **5b** (1.3 equiv.), Quinuclidine (1.8 equiv.), $BaTiO_3$ (4 equiv.)	30Hz/90 min	52
**10**	**1a (1 equiv.), 2 (1.1 equiv.), 5b (1.3 equiv.), DABCO (1.4 equiv.), $BaTiO_3$ (4 equiv.)**	**30Hz/90 min**	**64**
	Reactions in solution		
11	**1a** (1 equiv.), **2** (1.1 equiv.), **5b** (1.3 equiv.), DABCO (1.4 equiv.), $BaTiO_3$ (4 equiv.), MeOH, reflux	------/24 h	0
12	**1a** (1 equiv.), **2** (1.1 equiv.), **5b** (1.3 equiv.), DABCO (1.4 equiv.), $BaTiO_3$ (4 equiv.), CH_3CN, reflux	------/24 h	0
13	**1a** (1 equiv.), **2** (1.1 equiv.), **5b** (1.3 equiv.), DABCO (1.4 equiv.), $BaTiO_3$ (4 equiv.), 1,4-dioxane, reflux	------/24 h	0
14	**1a** (1 equiv.), **2** (1.1 equiv.), **5b** (1.3 equiv.), DABCO (1.4 equiv.), $BaTiO_3$ (4 equiv.), THF, reflux	------/24 h	0
15	**1a** (1 equiv.), **2** (1.1 equiv.), **5b** (1.3 equiv.), DABCO (1.4 equiv.), $BaTiO_3$ (4 equiv.), DMF, 100 °C	------/24 h	0
16	**1a** (1 equiv.), **2** (1.1 equiv.), **5b** (1.3 equiv.), DABCO (1.4 equiv.), $BaTiO_3$ (4 equiv.), DMF, 130 °C	------/24 h	0

Reaction procedure with optimised reaction conditions.

General procedure for the in-solution attempts:

With aryl boronic acids: Inside a glovebox, starting amide (1 mmol, 1 equiv.), pyrylium tetrafluoroborate **2** (185 mg, 1.1 mmol, 1.1 equiv.), $BaTiO_3$ (933 mg, 4 mmol, 4 equiv) and an appropriate aryl boronic acid (1.3 mmol, 1.3 equiv.), freshly sublimated DABCO (157 mg, 1.4 mmol, 1.4 equiv.) were weighed and placed successively into an Ace Pressure Tube equipped with a magnetic stir bar. Finally, 12 mL of dry methanol (or other solvents)

were added inside the glovebox, then the reaction vessel was capped with a stopper. Subsequently, the Pressure Tube was taken out of the glovebox and heated at appropriate temperature. Upon completion, the reaction mixture was cooled to room temperature and analysed by TLC and GS MS. Finally, the reaction mixture was concentrated under vacuum, the formed crude was washed with water, filtrated and dried. The residue was subjected to preparative column chromatography on Silica gel using hexane/ethyl acetate mixtures.

With aryl trialkoxysilanes: Inside a glovebox, starting amide (1 mmol, 1 equiv.), pyrylium tetrafluoroborate **2** (1.1 mmol, 1.1 equiv., 185 mg), $BaTiO_3$ (4 mmol, 4 equiv., 933 mg) and an appropriate aryl trialkoxysilane (1.3 mmol, 1.3 equiv.), freshly sublimated DABCO (1.4 mmol, 1.4 equiv., 157 mg) were weighed and placed successively into an Ace Pressure Tube equipped with a magnetic stir bar. Finally, 12 mL of dry methanol (or other solvents) were added inside the glovebox, then the reaction vessel was capped with a stopper. Subsequently, the Pressure Tube was taken out of the glovebox and heated at appropriate temperature. Upon completion, the reaction mixture was cooled to room temperature and analysed by TLC and GS MS. Finally, the reaction mixture was concentrated under vacuum, the formed crude was washed with water, filtrated and dried. The residue was subjected to preparative column chromatography on Silica gel using hexane/ethyl acetate mixtures.

General procedure for the solid-state synthesis:

General procedure for the solid-state synthesis of ketones 3 starting from boronic acids 4.

Inside a glovebox, a stainless steel 5 mL grinding vessel equipped with three balls (stainless steel, Φ=5 mm) was loaded consecutively with an appropriate amide (1 mmol, 1 equiv.), pyrylium tetrafluoroborate **2** (185 mg, 1.1 mmol, 1.1 equiv.), $BaTiO_3$ (933 mg, 4 mmol, 4 equiv.) and an appropriate aryl boronic acid (1.3 mmol, 1.3 equiv.), freshly sublimated DABCO (157 mg, 1.4 mmol, 1.4 equiv.). Lastly, freshly distilled 1,4-dioxane (0.25 mL) was added and the reaction vessel was properly sealed. The reaction vessel was installed on the ball mill outside the glovebox and the content was pulverized at 30Hz for 90 minutes. After completion of the reaction, the content of the vessel was directly subjected to flash column chromatography on silica gel to isolate the desired compound using gradient elution.

The gram scale synthesis was performed with 10 mmol of the starting amine in 25 mL grinding vessel using four balls Φ=10 mm balls.

General procedure for the solid-state synthesis of ketones 3 starting from aryl trialkoxysilane 5.

Inside a glovebox, a stainless steel 5 mL grinding vessel equipped with three balls (stainless steel, Φ=5 mm) was loaded consecutively with an appropriate amide (1 mmol, 1 equiv.), pyrylium tetrafluoroborate **2** (185 mg, 1.1 mmol, 1.1 equiv.), $BaTiO_3$ (933 mg, 4 mmol, 4 equiv.) and an appropriate aryl trialkoxysilane (1.3 mmol, 1.3 equiv.), freshly sublimated DABCO (157 mg, 1.4 mmol, 1.4 equiv.). Lastly, freshly distilled 1,4-dioxane (0.25 mL) was added and the reaction vessel was properly sealed. The reaction vessel was installed on the ball mill outside the glovebox and the content was pulverized at 30Hz for 90 minutes. After completion of the reaction, the content of the vessel was directly subjected to flash column chromatography on silica gel to isolate the desired compound using gradient elution.

The gram scale synthesis was performed with 10 mmol of the starting amine in 25 mL grinding vessel four balls Φ=10 mm balls.

General procedure for the solid-state synthesis of amides 7 starting from urea 6.

Inside a glovebox, a stainless steel 5 mL grinding vessel equipped with three balls (stainless steel, Φ=5 mm) was loaded consecutively with an appropriate urea (1 mmol, 1 equiv.), pyrylium tetrafluoroborate **2** (185 mg, 1.1 mmol, 1.1 equiv.), $BaTiO_3$ (933 mg, 4 mmol, 4 equiv.) and an appropriate aryl boronic acid (1.3 mmol, 1.3 equiv.), freshly sublimated DABCO (157 mg, 1.4 mmol, 1.4 equiv.). Lastly, freshly distilled 1,4-dioxane (0.25 mL) was added and the reaction vessel was properly sealed. The reaction vessel was installed on the ball mill outside the glovebox and the content was pulverized at 30Hz for 90 minutes. After completion of the reaction, the content of the vessel was directly subjected to flash column chromatography on silica gel to isolate the desired compound using gradient elution.

The gram scale synthesis was performed with 10 mmol of the starting amine in 25 mL grinding vessel using four balls Φ=10 mm balls.

General procedure for the solid-state synthesis of amide 3k starting from pyridinium salt 9 and boronic acid 4k.

Inside a glovebox, a stainless steel 5 mL grinding vessel equipped with three balls (stainless steel, Φ=5 mm) was loaded consecutively with the freshly prepared pyridinium salt (1 mmol, 1 equiv.), pyrylium tetrafluoroborate **2** (185 mg, 1.1 mmol, 1.1 equiv.), $BaTiO_3$ (933 mg, 4 mmol, 4 equiv.) and aryl boronic acid **4k** (1.3 mmol, 1.3 equiv.) and freshly sublimated DABCO (157 mg, 1.4 mmol, 1.4 equiv.). Lastly, freshly distilled 1,4-dioxane (0.25 mL) was added and the reaction vessel was properly sealed. The reaction vessel was installed on the ball mill outside the glovebox and the content was pulverized at 30Hz for 90 minutes. After completion of the reaction, the content of the vessel was directly subjected to flash column chromatography on silica gel to isolate the desired compound using gradient elution.

General procedure for the solid-state synthesis of amide 8 following one-pot two-step procedure starting from amide 1x and dibenzylamine.

Inside a glovebox, a stainless steel 5 mL grinding vessel equipped with three balls (stainless steel, Φ=5 mm) was loaded consecutively with the freshly prepared pyridinium salt (1 mmol, 1 equiv.), pyrylium tetrafluoroborate **2** (185 mg, 1.1 mmol, 1.1 equiv.), $BaTiO_3$ (933 mg, 4 mmol, 4 equiv.) and freshly sublimated DABCO (157 mg, 1.4 mmol, 1.4 equiv.). Lastly, freshly distilled 1,4-dioxane (0.25 mL) was added and the reaction vessel was properly sealed. The reaction vessel was installed on the ball mill outside the glovebox and the content was pulverized at 30Hz for 35 minutes. Afterwords, dibenzylamine (296 mg, 1.5 mmol, 1.5 equiv.) was loaded inside the glovebox and the reaction vessel was pulverized at 30Hz for another 45 minutes. After completion of the reaction, the content of the vessel was directly subjected to flash column chromatography on silica gel to isolate the desired compound using gradient elution.

(B) Characterization of products.

phenyl(p-tolyl)methanone 3a

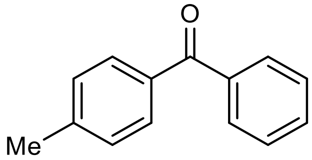

The title compound was prepared starting from an amide **1a** (121 mg, 1 mmol, 1 equiv.), pyrylium tetrafluoroborate **2** (185 mg, 1.1 mmol, 1.1 equiv.), $BaTiO_3$ (933 mg, 4 mmol, 4 equiv.), aryl boronic acid **4b** (177mg, 1.3 mmol, 1.3 equiv.), DABCO (157 mg, 1.4 mmol, 1.4 equiv.) and 1,4-dioxane (0.25 mL). The purification was accomplished by column chromatography on silica gel to provide the desired **3a** (176 mg, 0.90 mmol, 90%). The gram scale synthesis was performed on 10 mmol of starting amide **1a** and the product **3a** was prepared 85% in yield (1.66 g, 8.5 mmol).

Alternatively, the title compound was prepared starting from an amide **1b** (135 mg, 1 mmol, 1 equiv.), pyrylium tetrafluoroborate **2** (185 mg, 1.1 mmol, 1.1 equiv.), $BaTiO_3$ (933 mg, 4 mmol, 4 equiv.), aryl boronic acid **4a** (158 mg, 1.3 mmol, 1.3 equiv.), DABCO (157 mg, 1.4 mmol, 1.4 equiv.) and 1,4-dioxane (0.25 mL). The purification was accomplished by column chromatography on silica gel to provide the desired **3a** (180 mg, 0.92 mmol, 92%).

Alternatively, the title compound was prepared starting from an amide **1a** (121 mg, 1 mmol, 1 equiv.), pyrylium tetrafluoroborate **2** (185 mg, 1.1 mmol, 1.1 equiv.), $BaTiO_3$ (933 mg, 4 mmol, 4 equiv.), aryl trialkoxysilane **5b** (276 mg, 1.3 mmol, 1.3 equiv.), DABCO (157 mg, 1.4 mmol, 1.4 equiv.) and 1,4-dioxane (0.25 mL). The purification was accomplished by column chromatography on silica gel to provide the desired **3a** (125 mg, 0.64 mmol, 64%). The gram scale synthesis was performed on 10 mmol of starting amide **1a** and the product **3a** was prepared 53% in yield (1.04 g, 5.3 mmol).

Alternatively, the title compound was prepared starting from an amide **1b** (135 mg, 1 mmol, 1 equiv.), pyrylium tetrafluoroborate **2** (185 mg, 1.1 mmol, 1.1 equiv.), $BaTiO_3$ (933 mg, 4 mmol, 4 equiv.), aryl trialkoxysilane **5a** (257 mg, 1.3 mmol, 1.3 equiv.), DABCO (157 mg, 1.4 mmol, 1.4 equiv.) and 1,4-dioxane (0.25 mL). The purification was accomplished by column chromatography on silica gel to provide the desired **3a** (117 mg, 0.60 mmol, 60%).

Beige solid, mp 55-57 ^oC. ^{1}**H NMR** (400 MHz, $CDCl_3$): δ 2.42 (s, 3H, Me), 7.26 (d, 2H, *J* = 7.2 Hz, CH_{Ar}), 7.45 (t, 2H, *J* = 7.7 Hz, CH_{Ar}), 7.55 (t, 1H, *J* = 7.8 Hz, CH_{Ar}), 7.71 (d, 2H, *J* = 8.4 Hz, CH_{Ar}), 7.77 (d, 2H, *J* = 7.7 Hz, CH_{Ar}).

^{13}**C{1H} NMR** (100 MHz, $CDCl_3$): δ 21.6, 128.1, 128.9, 129.8, 130.2, 132.1, 134.8, 137.9, 143.1, 194.4.

Anal. calcd. for $C_{14}H_{12}O$: C, 85.68; H, 6.16. Found: C, 85.59; H, 6.13.

phenyl(o-tolyl)methanone 3b

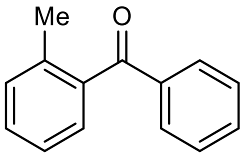

The title compound was prepared starting from an amide **1a** (121 mg, 1 mmol, 1 equiv.), pyrylium tetrafluoroborate **2** (185 mg, 1.1 mmol, 1.1 equiv.), $BaTiO_3$ (933 mg, 4 mmol, 4 equiv.), aryl boronic acid **4c** (177 mg, 1.3 mmol, 1.3 equiv.), DABCO (157 mg, 1.4 mmol, 1.4 equiv.) and 1,4-dioxane (0.25 mL). The purification was accomplished by column chromatography on silica gel to provide the desired **3b** (155 mg, 0.79 mmol, 79%).
Alternatively, the title compound was prepared starting from an amide **1c** (135 mg, 1 mmol, 1 equiv.), pyrylium tetrafluoroborate **2** (185 mg, 1.1 mmol, 1.1 equiv.), $BaTiO_3$ (933 mg, 4 mmol, 4 equiv.), aryl boronic acid **4a** (158 mg, 1.3 mmol, 1.3 equiv.), DABCO (157 mg, 1.4 mmol, 1.4 equiv.) and 1,4-dioxane (0.25 mL). The purification was accomplished by column chromatography on silica gel to provide the desired **3b** (178 mg, 0.91 mmol, 91%).
Alternatively, the title compound was prepared starting from an amide **1a** (121 mg, 1 mmol, 1 equiv.), pyrylium tetrafluoroborate **2** (185 mg, 1.1 mmol, 1.1 equiv.), $BaTiO_3$ (933 mg, 4 mmol, 4 equiv.), aryl trialkoxysilane **5c** (257 mg, 1.3 mmol, 1.3 equiv.), DABCO (157 mg, 1.4 mmol, 1.4 equiv.) and 1,4-dioxane (0.25 mL). The purification was accomplished by column chromatography on silica gel to provide the desired **3b** (100 mg, 0.51 mmol, 51%).
Alternatively, the title compound was prepared starting from an amide **1c** (135 mg, 1 mmol, 1 equiv.), pyrylium tetrafluoroborate **2** (185 mg, 1.1 mmol, 1.1 equiv.), $BaTiO_3$ (933 mg, 4 mmol, 4 equiv.), aryl trialkoxysilane **5a** (257 mg, 1.3 mmol, 1.3 equiv.), DABCO (157 mg, 1.4 mmol, 1.4 equiv.) and 1,4-dioxane (0.25 mL). The purification was accomplished by column chromatography on silica gel to provide the desired **3b** (114 mg, 0.58 mmol, 58%).
Colorless oil. **^{1}H NMR** (400 MHz, $CDCl_3$): δ 2.33 (s, 3H, Me), 7.23 – 7.32 (m, 3H, CH_{Ar}), 7.38 (t, 1H, J = 7.5 Hz, CH_{Ar}), 7.44 (t, 2H, J = 7.8 Hz, CH_{Ar}), 7.57 (t, 1H, J = 7.8 Hz, CH_{Ar}), 7.79 (d, 2H, J = 7.7 Hz, CH_{Ar}).
^{13}C{1H} NMR (100 MHz, $CDCl_3$): δ 19.9, 125.1, 128.4, 130.1, 130.2, 130.9, 133.1, 136.7, 137.7, 138.6, 198.6.
MS (GC, 70eV): m/z (%) = 196 (M^+, 62), 195 (100), 119 (25), 105 (20).
Anal. calcd. for $C_{14}H_{12}O$: C, 85.68; H, 6.16. Found: C, 85.81; H, 6.08.

(2,5-difluorophenyl)(phenyl)methanone 3c

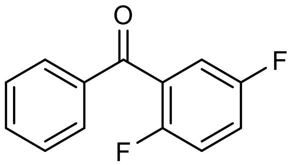

The title compound was prepared starting from an amide **1a** (121 mg, 1 mmol, 1 equiv.), pyrylium tetrafluoroborate **2** (185 mg, 1.1 mmol, 1.1 equiv.), $BaTiO_3$ (933 mg, 4 mmol, 4 equiv.), aryl boronic acid **4d** (1205 mg, 1.3 mmol, 1.3 equiv.), DABCO (157 mg, 1.4 mmol, 1.4 equiv.) and 1,4-dioxane (0.25 mL). The purification was accomplished by column chromatography on silica gel to provide the desired **3c** (183 mg, 0.84 mmol, 84%).
Alternatively, the title compound was prepared starting from an amide **1d** (157 mg, 1 mmol, 1 equiv.), pyrylium tetrafluoroborate **2** (185 mg, 1.1 mmol, 1.1 equiv.), $BaTiO_3$ (933 mg, 4 mmol, 4 equiv.), aryl boronic acid **4a** (158 mg, 1.3 mmol, 1.3 equiv.), DABCO (157 mg, 1.4 mmol, 1.4 equiv.) and 1,4-dioxane (0.25 mL). The purification was accomplished by column chromatography on silica gel to provide the desired **3c** (192 mg, 0.88 mmol, 88%).
Alternatively, the title compound was prepared starting from an amide **1a** (121 mg, 1 mmol, 1 equiv.), pyrylium tetrafluoroborate **2** (185 mg, 1.1 mmol, 1.1 equiv.), $BaTiO_3$ (933 mg, 4 mmol, 4 equiv.), aryl trialkoxysilane **5d** (304 mg, 1.3 mmol, 1.3 equiv.), DABCO (157 mg, 1.4 mmol, 1.4 equiv.) and 1,4-dioxane (0.25 mL). The purification was accomplished by column chromatography on silica gel to provide the desired **3c** (137 mg, 0.63 mmol, 63%).
Light yellow liquid. **^{1}H NMR** (500 MHz, $CDCl_3$): δ 6.87 – 6.91 (m, 1H, CH_{Ar}), 6.97 – 7.01 (m, 1H, CH_{Ar}), 7.45 – 7.48 (m, 2H, CH_{Ar}), 7.58 – 7.61 (m, 2H, CH_{Ar}), 7.79 – 7.81 (m, 2H, CH_{Ar}).
^{13}C{1H} NMR (125 MHz, $CDCl_3$): δ 104.7 (t, J_{CF} = 25.6 Hz), 111.9 (dd, J_{CF} = 21.5 Hz, J_{CF} = 3.5 Hz), 123.4 (dd, J_{CF} = 14.5 Hz, J_{CF} = 3.7 Hz), 128.5, 129.7, 132.6 (dd, J_{CF} = 10.4 Hz, J_{CF} = 4.3 Hz), 133.5, 137.4, 160.9 (dd, $^1J_{CF}$ = 256 Hz, J_{CF} = 12.2 Hz), 164.9 (dd, $^1J_{CF}$ = 255 Hz, J_{CF} = 11.7 Hz), 192.3.
MS (GC, 70eV): m/z (%) = 218 (M^+, 65), 141 (68), 113 (34), 105 (100).
Anal. calcd. for $C_{13}H_8F_2O$: C, 71.56; H, 3.70. Found: C, 71.63; H, 3.68.

(3,5-difluorophenyl)(phenyl)methanone 3d

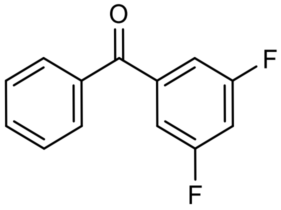

The title compound was prepared starting from an amide **1a** (121 mg, 1 mmol, 1 equiv.), pyrylium tetrafluoroborate **2** (185 mg, 1.1 mmol, 1.1 equiv.), $BaTiO_3$ (933 mg, 4 mmol, 4 equiv.), aryl boronic acid **4e** (205 mg, 1.3 mmol, 1.3 equiv.), DABCO (157 mg, 1.4 mmol, 1.4 equiv.) and 1,4-dioxane (0.25 mL). The purification was accomplished by column chromatography on silica gel to provide the desired **3d** (190 mg, 0.87 mmol, 87%).
Alternatively, the title compound was prepared starting from an amide **1e** (157 mg, 1 mmol, 1 equiv.), pyrylium tetrafluoroborate **2** (185 mg, 1.1 mmol, 1.1 equiv.), $BaTiO_3$ (933 mg, 4 mmol, 4 equiv.), aryl boronic acid **4a** (158 mg, 1.3 mmol, 1.3 equiv.), DABCO (157 mg, 1.4 mmol, 1.4 equiv.) and

1,4-dioxane (0.25 mL). The purification was accomplished by column chromatography on silica gel to provide the desired **3d** (203 mg, 0.93 mmol, 93%).

Alternatively, the title compound was prepared starting from an amide **1e** (157 mg, 1 mmol, 1 equiv.), pyrylium tetrafluoroborate **2** (185 mg, 1.1 mmol, 1.1 equiv.), $BaTiO_3$ (933 mg, 4 mmol, 4 equiv.), aryl trialkoxysilane **5a** (257 mg, 1.3 mmol, 1.3 equiv.), DABCO (157 mg, 1.4 mmol, 1.4 equiv.) and 1,4-dioxane (0.25 mL). The purification was accomplished by column chromatography on silica gel to provide the desired **3d** (142 mg, 0.65 mmol, 65%).

Yellow solid, mp 58-59 °C. **^{1}H NMR** (500 MHz, DMSO-*d*$_6$): δ 7.32 – 7.37 (m, 2H, CH_{Ar}), 7.54 – 7.58 (m, 3H, CH_{Ar}), 7.67 – 7.70 (m, 1H, CH_{Ar}), 7.74 – 7.76 (m, 2H, CH_{Ar}).

^{13}C{1H} NMR (125 MHz, DMSO-*d*$_6$): δ 117.8 (m), 122.6 (m), 136.7, 139.8, 143.4, 145.8, 150.4 (t, J_{CF} = 8 Hz), 172.1 (dd, $^1J_{CF}$ = 249 Hz, J_{CF} = 12.2 Hz), 203.2.

Anal. calcd. for $C_{13}H_8F_2O$: C, 71.56; H, 3.70. Found: C, 71.49; H, 3.79.

bis(3-fluorophenyl)methanone 3e

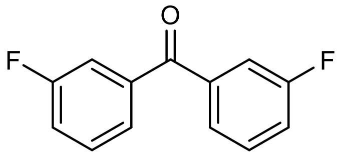

The title compound was prepared starting from an amide **1f** (139 mg, 1 mmol, 1 equiv.), pyrylium tetrafluoroborate **2** (185 mg, 1.1 mmol, 1.1 equiv.), $BaTiO_3$ (933 mg, 4 mmol, 4 equiv.), aryl boronic acid **4f** (182 mg, 1.3 mmol, 1.3 equiv.), DABCO (157 mg, 1.4 mmol, 1.4 equiv.) and 1,4-dioxane (0.25 mL). The purification was accomplished by column chromatography on silica gel to provide the desired **3e** (200 mg, 0.92 mmol, 92%). The gram scale synthesis was performed on 10 mmol of starting amide **1f** and the product **3e** was prepared 90% in yield (1.96 g, 9.0 mmol).

Alternatively, the title compound was prepared starting from an amide **1f** (139 mg, 1 mmol, 1 equiv.), pyrylium tetrafluoroborate **2** (185 mg, 1.1 mmol, 1.1 equiv.), $BaTiO_3$ (933 mg, 4 mmol, 4 equiv.), aryl trialkoxysilane **5e** (281 mg, 1.3 mmol, 1.3 equiv.), DABCO (157 mg, 1.4 mmol, 1.4 equiv.) and 1,4-dioxane (0.25 mL). The purification was accomplished by column chromatography on silica gel to provide the desired **3e** (124 mg, 0.57 mmol, 57%).

White solid, mp 60-61 °C. **^{1}H NMR** (400 MHz, $CDCl_3$): δ 7.28 – 7.33 (m, 2H, CH_{Ar}), 7.45 – 7.47 (m, 1H, CH_{Ar}), 7.48 – 7.52 (m, 3H, CH_{Ar}), 7.55 – 7.58 (m, 2H, CH_{Ar}).

^{19}F NMR (376 MHz, $CDCl_3$): δ -111.6 (td, *J* = 8.6 Hz, *J* = 5.6 Hz).

^{13}C{1H} NMR (100 MHz, $CDCl_3$): δ 116.7 (d, J_{CF} = 22.6 Hz), 119.8 (d, J_{CF} = 21.8 Hz), 125.8 (d, J_{CF} = 3 Hz), 130.1 (d, J_{CF} = 7.8 Hz), 139.1 (d, J_{CF} = 6.5 Hz), 162.5 (d, $^1J_{CF}$ = 248.5 Hz), 193.8 (t, $^4J_{CF}$ = 2.2 Hz).

MS (GC, 70eV): m/z (%) = 218 (M^+, 52), 123 (100).

Anal. calcd. for $C_{13}H_8F_2O$: C, 71.56; H, 3.70. Found: C, 71.62; H, 3.83.

phenyl(2-(trifluoromethyl)phenyl)methanone 3f

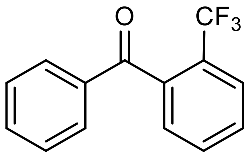

The title compound was prepared starting from an amide **1a** (121 mg, 1 mmol, 1 equiv.), pyrylium tetrafluoroborate **2** (185 mg, 1.1 mmol, 1.1 equiv.), $BaTiO_3$ (933 mg, 4 mmol, 4 equiv.), aryl boronic acid **4g** (247 mg, 1.3 mmol, 1.3 equiv.), DABCO (157 mg, 1.4 mmol, 1.4 equiv.) and 1,4-dioxane (0.25 mL). The purification was accomplished by column chromatography on silica gel to provide the desired **3f** (147 mg, 0.59 mmol, 59%).
White solid, mp 61-62 °C. **^{1}H NMR** (400 MHz, $CDCl_3$): δ 7.38 – 7.40 (m, 1H, CH_{Ar}), 7.44 – 7.48 (m, 2H, CH_{Ar}), 7.58 – 7.65 (m, 3H, CH_{Ar}), 7.77 – 7.79 (m, 3H, CH_{Ar}).
^{19}F NMR (376 MHz, $CDCl_3$): δ -58.04 (s, CF_3).
^{13}C{1H} NMR (100 MHz, $CDCl_3$): δ 123.6 (q, $^1J_{CF}$ = 273.9 Hz), 126.7 (q, J_{CF} = 4.7 Hz), 128.1, 128.2 (q, J_{CF} = 32.4 Hz), 128.5, 129.8, 130.2, 131.4, 133.9, 136.4, 138.3 (q, J_{CF} = 2.1 Hz).
MS (GC, 70eV): m/z (%) = 250 (M^+, 62), 173 (30), 105 (100).
Anal. calcd. for $C_{14}H_9F_3O$: C, 67.20; H, 3.63. Found: C, 67.09; H, 3.72.

(3,5-bis(trifluoromethyl)phenyl)(phenyl)methanone 3g

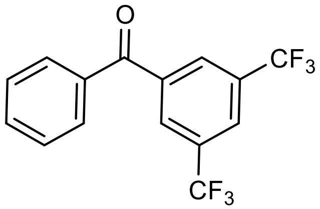

The title compound was prepared starting from an amide **1a** (121 mg, 1 mmol, 1 equiv.), pyrylium tetrafluoroborate **2** (185 mg, 1.1 mmol, 1.1 equiv.), $BaTiO_3$ (933 mg, 4 mmol, 4 equiv.), aryl boronic acid **4h** (335 mg, 1.3 mmol, 1.3 equiv.), DABCO (157 mg, 1.4 mmol, 1.4 equiv.) and 1,4-dioxane (0.25 mL). The purification was accomplished by column chromatography on silica gel to provide the desired **3g** (277 mg, 0.87 mmol, 87%). The gram scale synthesis was performed on 10 mmol of starting amide **1a** and the product **3g** was prepared 86% in yield (2.73 g, 8.6 mmol).
White viscous. **^{1}H NMR** (500 MHz, $CDCl_3$): δ 7.54 – 7.57 (m, 2H, CH_{Ar}), 7.66 – 7.69 (m, 1H, CH_{Ar}), 7.78 – 7.81 (m, 2H, CH_{Ar}), 8.10 (s, 1H, CH_{Ar}), 8.24 (s, 2H, CH_{Ar}).

^{13}C{1H} NMR (125 MHz, $CDCl_3$): δ 122.9 (q, $^1J_{CF}$ = 273 Hz), 125.6 (sep, $^3J_{CF}$ = 3.6 Hz), 128.8, 129.8 (m), 130, 132 (q, J_{CF} = 34 Hz), 133.6, 135.9, 139.4, 193.5.
MS (GC, 70eV): m/z (%) = 318 (M^+, 24), 213 (14), 105 (100).
Anal. calcd. for $C_{15}H_8F_6O$: C, 56.62; H, 2.53. Found: C, 56.55; H, 2.63.

(3-fluoro-4-(trifluoromethyl)phenyl)(phenyl)methanone 3h

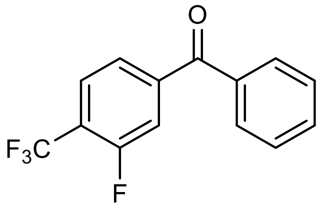

The title compound was prepared starting from an amide **1a** (121 mg, 1 mmol, 1 equiv.), pyrylium tetrafluoroborate **2** (185 mg, 1.1 mmol, 1.1 equiv.), $BaTiO_3$ (933 mg, 4 mmol, 4 equiv.), aryl boronic acid **4i** (270 mg, 1.3 mmol, 1.3 equiv.), DABCO (157 mg, 1.4 mmol, 1.4 equiv.) and 1,4-dioxane (0.25 mL). The purification was accomplished by column chromatography on silica gel to provide the desired **3h** (241 mg, 0.90 mmol, 90%).
White solid, mp 65-66 °C. **^{1}H NMR** (400 MHz, $CDCl_3$): δ 7.52 (t, 2H, J = 7.6 Hz, CH_{Ar}), 7.60 – 7.67 (m, 3H, CH_{Ar}), 7.75 (t, 1H, J = 7.3 Hz, CH_{Ar}), 7.79 – 7.81 (m, 2H, CH_{Ar}).
^{13}C{1H} NMR (100 MHz, $CDCl_3$): δ 118.0 (d, J_{CF} = 21.1 Hz), 121.5 (dq, J_{CF} = 33.1 Hz J_{CF} = 14.0 Hz), 122.2 (q, $^1J_{CF}$ = 272.8 Hz), 125.3 (d, J_{CF} = 4.3 Hz), 127.4 (m), 128.7, 130.1, 133.4, 136.2, 143.1 (d, J_{CF} = 6.8 Hz), 159.5 (dq, $^1J_{CF}$ = 258.1 Hz, J_{CF} = 2.0 Hz), 194.0 (d, J_{CF} = 1.0 Hz).
MS (GC, 70eV): m/z (%) = 268 (M^+, 41), 191 (16), 163 (22), 105 (100).
Anal. calcd. for $C_{14}H_8F_4O$: C, 62.69; H, 3.01. Found: C, 62.79; H, 2.96.

bis(3-(trifluoromethyl)phenyl)methanone 3i

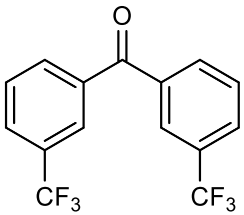

The title compound was prepared starting from an amide **1g** (189 mg, 1 mmol, 1 equiv.), pyrylium tetrafluoroborate **2** (185 mg, 1.1 mmol, 1.1 equiv.), $BaTiO_3$ (933 mg, 4 mmol, 4 equiv.), aryl boronic acid **4j** (247 mg, 1.3 mmol, 1.3 equiv.), DABCO (157 mg, 1.4 mmol, 1.4 equiv.) and 1,4-dioxane (0.25 mL). The purification was accomplished by column chromatography on silica gel to provide the desired **3i** (277 mg, 0.87 mmol, 87%).

Alternatively, the title compound was prepared starting from an amide **1g** (189 mg, 1 mmol, 1 equiv.), pyrylium tetrafluoroborate **2** (185 mg, 1.1 mmol, 1.1 equiv.), $BaTiO_3$ (933 mg, 4 mmol, 4 equiv.), aryl trialkoxysilane **5f** (345 mg, 1.3 mmol, 1.3 equiv.), DABCO (157 mg, 1.4 mmol, 1.4 equiv.) and 1,4-dioxane (0.25 mL). The purification was accomplished by column chromatography on silica gel to provide the desired **3i** (197 mg, 0.62 mmol, 62%).
White solid, mp 100-101 °C. **^{1}H NMR** (400 MHz, $CDCl_3$): δ 7.82 (t, 2H, *J* = 7.6 Hz, CH_{Ar}), 8.02 – 8.03 (m, 4H, CH_{Ar}), 8.07 (d, 2H, *J* = 7.8 Hz, CH_{Ar}).
^{13}C{1H} NMR (100 MHz, $CDCl_3$): δ 123.7 (q, $^1J_{CF}$ = 273 Hz), 125.9 (q, J_{CF} = 3.6 Hz), 129.4 (q, J_{CF} = 2.7 Hz), 129.5 (q, J_{CF} = 33.1 Hz), 130.0, 133.7, 137.2, 193.3.
MS (GC, 70eV): m/z (%) = 316 (M^+, 31), 301 (100), 183 (25).
Anal. calcd. for $C_{17}H_{17}BrO$: C, 64.37; H, 5.40. Found: C, 64.42; H, 5.33.

(2-chlorophenyl)(4-chlorophenyl)methanone 3j

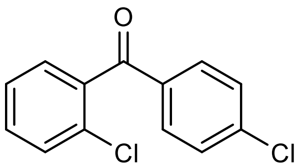

The title compound was prepared starting from an amide **1h** (156 mg, 1 mmol, 1 equiv.), pyrylium tetrafluoroborate **2** (185 mg, 1.1 mmol, 1.1 equiv.), $BaTiO_3$ (933 mg, 4 mmol, 4 equiv.), aryl boronic acid **4k** (203 mg, 1.3 mmol, 1.3 equiv.), DABCO (157 mg, 1.4 mmol, 1.4 equiv.) and 1,4-dioxane (0.25 mL). The purification was accomplished by column chromatography on silica gel to provide the desired **3j** (236 mg, 0.94 mmol, 94%).
Alternatively, the title compound was prepared starting from an amide **1i** (156 mg, 1 mmol, 1 equiv.), pyrylium tetrafluoroborate **2** (185 mg, 1.1 mmol, 1.1 equiv.), $BaTiO_3$ (933 mg, 4 mmol, 4 equiv.), aryl boronic acid **4l** (203 mg, 1.3 mmol, 1.3 equiv.), DABCO (157 mg, 1.4 mmol, 1.4 equiv.) and 1,4-dioxane (0.25 mL). The purification was accomplished by column chromatography on silica gel to provide the desired **3j** (208 mg, 0.83 mmol, 83%).
Alternatively, the title compound was prepared starting from an amide **1h** (156 mg, 1 mmol, 1 equiv.), pyrylium tetrafluoroborate **2** (185 mg, 1.1 mmol, 1.1 equiv.), $BaTiO_3$ (933 mg, 4 mmol, 4 equiv.), aryl trialkoxysilane **5g** (279 mg, 1.3 mmol, 1.3 equiv.), DABCO (157 mg, 1.4 mmol, 1.4 equiv.) and 1,4-dioxane (0.25 mL). The purification was accomplished by column chromatography on silica gel to provide the desired **3j** (206 mg, 0.82 mmol, 82%).
White solid, mp 64-65 °C. **^{1}H NMR** (400 MHz, $CDCl_3$): δ 7.37 – 7.38 (m, 2H, CH_{Ar}), 7.44 (dt, 2H, *J* = 8.7 Hz, *J* = 2.0 Hz, CH_{Ar}), 7.45 – 7.46 (m, 2H, CH_{Ar}), 7.74 (dt, 2H, *J* = 8.7 Hz, *J* = 2.0 Hz, CH_{Ar}).
^{13}C{1H} NMR (100 MHz, $CDCl_3$): δ 126.8, 129.0, 129.1, 130.1, 131.3, 131.4, 134.9, 138.1, 140.3, 194.0.
MS (GC, 70eV): m/z (%) = 252 (18), 250 (M^+, 28), 139 (100), 111 (27).

Anal. calcd. for $C_{13}H_8Cl_2O$: C, 62.18; H, 3.21. Found: C, 62.29; H, 3.27.

(3-chlorophenyl)(4-chlorophenyl)methanone 3k

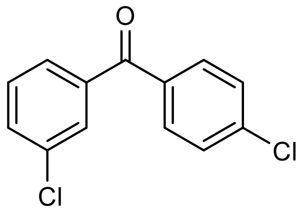

The title compound was prepared starting from an amide **1j** (156 mg, 1 mmol, 1 equiv.), pyrylium tetrafluoroborate **2** (185 mg, 1.1 mmol, 1.1 equiv.), $BaTiO_3$ (933 mg, 4 mmol, 4 equiv.), aryl boronic acid **4k** (203 mg, 1.3 mmol, 1.3 equiv.), DABCO (157 mg, 1.4 mmol, 1.4 equiv.) and 1,4-dioxane (0.25 mL). The purification was accomplished by column chromatography on silica gel to provide the desired **3k** (213 mg, 0.85 mmol, 85%).
Alternatively, the title compound was prepared starting from an amide **1j** (156 mg, 1 mmol, 1 equiv.), pyrylium tetrafluoroborate **2** (185 mg, 1.1 mmol, 1.1 equiv.), $BaTiO_3$ (933 mg, 4 mmol, 4 equiv.), aryl trialkoxysilane **5g** (279 mg, 1.3 mmol, 1.3 equiv.), DABCO (157 mg, 1.4 mmol, 1.4 equiv.) and 1,4-dioxane (0.25 mL). The purification was accomplished by column chromatography on silica gel to provide the desired **3k** (123 mg, 0.49 mmol, 49%).
Alternatively, the title compound was prepared starting from freshly prepared pyridinium salt (254 mg, 1 mmol, 1 equiv.), pyrylium tetrafluoroborate **2** (185 mg, 1.1 mmol, 1.1 equiv.), $BaTiO_3$ (933 mg, 4 mmol, 4 equiv.) and aryl boronic acid **4k** (203 mg, 1.3 mmol, 1.3 equiv.), DABCO (157 mg, 1.4 mmol, 1.4 equiv.) and freshly distilled 1,4-dioxane (0.25 mL). The purification was accomplished by column chromatography on silica gel to provide the desired **3k** (176 mg, 0.70 mmol, 70%).
Yellowish, mp 112-113 °C. **^{1}H NMR** (400 MHz, $CDCl_3$): δ 7.42 (t, 1H, *J* = 7.8 Hz, CH_{Ar}), 7.45 – 7.48 (m, 2H, CH_{Ar}), 7.56 (ddd, 1H, *J* = 8 Hz, *J* = 2.2 Hz, *J* = 1.2 Hz, CH_{Ar}), 7.61 – 7.64 (m, 1H, CH_{Ar}), 7.71 – 7.75 (m, 3H, CH_{Ar}).
^{13}C{1H} NMR (100 MHz, $CDCl_3$): δ 128, 128.6, 129.8, 131.4, 132.6, 134.7, 135.2, 138.9, 139.4, 193.9.
MS (GC, 70eV): m/z (%) = 252 (18), 250 (M^+, 28), 139 (100), 111 (35).
Anal. calcd. for $C_{13}H_8Cl_2O$: C, 62.18; H, 3.21. Found: C, 62.29; H, 3.27.

bis(4-bromophenyl)methanone 3l

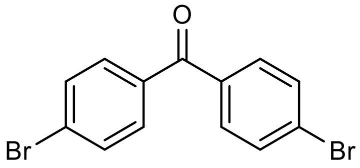

The title compound was prepared starting from an amide **1k** (200 mg, 1 mmol, 1 equiv.), pyrylium tetrafluoroborate **2** (185 mg, 1.1 mmol, 1.1 equiv.), $BaTiO_3$ (933 mg, 4 mmol, 4 equiv.), aryl boronic acid **4m** (260 mg, 1.3 mmol, 1.3 equiv.), DABCO (157 mg, 1.4 mmol, 1.4 equiv.) and 1,4-dioxane (0.25 mL). The purification was accomplished by column chromatography on silica gel to provide the desired **3l** (275 mg, 0.81 mmol, 81%).
Light brown solid, mp 173-174 °C. **^{1}H NMR** (400 MHz, $CDCl_3$): δ 7.62 (d, 4H, *J* = 8.8 Hz, CH_{Ar}), 7.65 (d, 4H, *J* = 8.8 Hz, CH_{Ar}).
^{13}C{1H} NMR (100 MHz, $CDCl_3$): δ 127.8, 131.4, 131.8, 135.9, 194.5.
Anal. calcd. for $C_{13}H_8Br_2O$: C, 45.92; H, 2.37. Found: C, 45.83; H, 2.42.

bis(4-(diethylamino)phenyl)methanone 3m

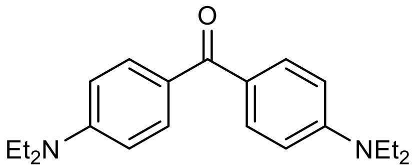

The title compound was prepared starting from an amide **1l** (164 mg, 1 mmol, 1 equiv.), pyrylium tetrafluoroborate **2** (185 mg, 1.1 mmol, 1.1 equiv.), $BaTiO_3$ (933 mg, 4 mmol, 4 equiv.), aryl boronic acid **4n** (215 mg, 1.3 mmol, 1.3 equiv.), DABCO (157 mg, 1.4 mmol, 1.4 equiv.) and 1,4-dioxane (0.25 mL). The purification was accomplished by column chromatography on silica gel to provide the desired **3m** (240 mg, 0.74 mmol, 74%).
Yellow solid, mp 90-91 °C. **^{1}H NMR** (400 MHz, $CDCl_3$): δ 1.22 (s, 12H, 4xCH_3), 3.43 (m, 8H, 4xCH_2), 7.65 (d, 4H, ^3J = 8.1 Hz, CH), 7.75 (d, 4H, *J* = 8.1 Hz, CH_{Ar}).
^{13}C{1H} NMR (100 MHz, $CDCl_3$): δ 12.8, 44.5, 110.0, 125.6, 132.5, 150.3, 193.8.
MS (GC, 70eV): m/z (%) = 342 (38), 309 (100), 265 (15).
Anal. calcd. for $C_{21}H_{28}N_2O$: C, 77.74; H, 8.70; N, 8.63. Found: C, 77.69; H, 8.73; N, 8.55.

(4-hydroxyphenyl)(phenyl)methanone 3n

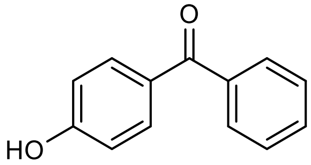

The title compound was prepared starting from an amide **1a** (121 mg, 1 mmol, 1 equiv.), pyrylium tetrafluoroborate **2** (185 mg, 1.1 mmol, 1.1 equiv.), $BaTiO_3$ (933 mg, 4 mmol, 4 equiv.), aryl boronic acid **4o** (179 mg, 1.3 mmol, 1.3 equiv.), DABCO (157 mg, 1.4 mmol, 1.4 equiv.) and 1,4-dioxane (0.25 mL). The purification was accomplished by column chromatography on silica gel to provide the desired **3n** (158 mg, 0.80 mmol, 80%).
White solid, mp 132-133 °C. **^{1}H NMR** (400 MHz, $CDCl_3$): δ 6.95 (d, 2H, *J* = 8.2 Hz, CH_{Ar}), 7.47 (t, 2H, *J* = 7..4 Hz, CH_{Ar}), 7.55 – 7.58 (m, 1H, CH_{Ar}), 7.75 – 7.79 (m, 5H, OH, CH_{Ar}).

^{13}C{1H} NMR (100 MHz, $CDCl_3$): δ 115.4, 128.3, 129.4, 129.9, 132.3, 133.2, 138.0, 161.0, 197.1.
Anal. calcd. for $C_{13}H_{10}O_2$: C, 78.77; H, 5.09. Found: C, 78.82; H, 5.16.

bis(4-bromophenyl)methanone 3o

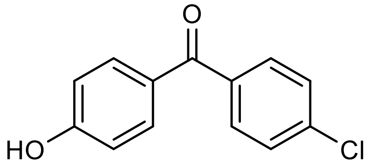

The title compound was prepared starting from an amide **1m** (137 mg, 1 mmol, 1 equiv.), pyrylium tetrafluoroborate **2** (185 mg, 1.1 mmol, 1.1 equiv.), $BaTiO_3$ (933 mg, 4 mmol, 4 equiv.), aryl boronic acid **4k** (203 mg, 1.3 mmol, 1.3 equiv.), DABCO (157 mg, 1.4 mmol, 1.4 equiv.) and 1,4-dioxane (0.25 mL). The purification was accomplished by column chromatography on silica gel to provide the desired **3o** (183 mg, 0.79 mmol, 79%).
Beige solid, mp 177-178 °C. **^{1}H NMR** (400 MHz, DMSO-d_6): δ 6.90 (d, 2H, *J* = 9.2 Hz, CH_{Ar}), 7.56 (d, 2H, *J* = 8.5 Hz, CH_{Ar}), 7.66 (t, 4H, *J* = 7.3 Hz, CH_{Ar}).
^{13}C{1H} NMR (100 MHz, DMSO-d_6): δ 115.4, 127.6, 128.5, 131.1, 132.6, 136.8, 162.2, 193.2.
MS (GC, 70eV): m/z (%) = 232 (M^+, 54), 139 (21), 21 (100), 111 (14).
Anal. calcd. for $C_{13}H_9ClO_2$: C, 67.11; H, 3.90. Found: C, 67.13; H, 3.99.

bis(4-hydroxyphenyl)methanone 3p

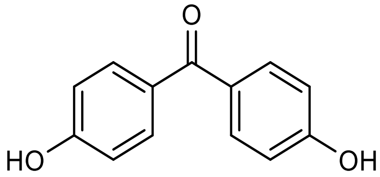

The title compound was prepared starting from an amide **1m** (137 mg, 1 mmol, 1 equiv.), pyrylium tetrafluoroborate **2** (185 mg, 1.1 mmol, 1.1 equiv.), $BaTiO_3$ (933 mg, 4 mmol, 4 equiv.), aryl boronic acid **4o** (165 mg, 1.3 mmol, 1.3 equiv.), DABCO (157 mg, 1.4 mmol, 1.4 equiv.) and 1,4-dioxane (0.25 mL). The purification was accomplished by column chromatography on silica gel to provide the desired **3p** (162 mg, 0.76 mmol, 76%).
Beige solid, mp 212-213 °C. **^{1}H NMR** (400 MHz, $CDCl_3$): δ 6.88 (d, 4H, *J* = 8.5 Hz, CH_{Ar}), 7.61 (d, 4H, *J* = 8.0 Hz, CH_{Ar}), 10.29 (s, 2H, OH).
^{13}C{1H} NMR (100 MHz, $CDCl_3$): δ 115.2, 129.0, 132.2, 161.4, 193.3.
Anal. calcd. for $C_{13}H_{10}O_3$: C, 72.89; H, 4.71. Found: C, 72.80; H, 4.69.

phenyl(2,3,4-trihydroxyphenyl)methanone 3q

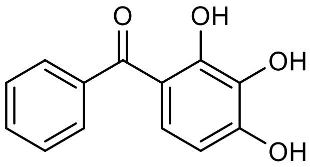

The title compound was prepared starting from an amide **1a** (121 mg, 1 mmol, 1 equiv.), pyrylium tetrafluoroborate **2** (185 mg, 1.1 mmol, 1.1 equiv.), $BaTiO_3$ (933 mg, 4 mmol, 4 equiv.), aryl boronic acid **4p** (221 mg, 1.3 mmol, 1.3 equiv.), DABCO (157 mg, 1.4 mmol, 1.4 equiv.) and 1,4-dioxane (0.25 mL). The purification was accomplished by column chromatography on silica gel to provide the desired **3q** (165 mg, 0.72 mmol, 72%). Yelloish solid, mp 139-140 °C. **^{1}H NMR** (400 MHz, $CDCl_3$): δ 5.94 (br. S, 2H, 2xOH), 6.50 (d, 1H, J = 9.1 Hz, CH_{Ar}), 7.15 (d, 1H, J = 9.1 Hz, CH_{Ar}), 7.47 – 7.51 (m, 2H, CH_{Ar}), 7.55 – 7.59 (m, 1H, CH_{Ar}), 7.67 (d, 2H, J = 7.4 Hz, CH_{Ar}), 10.68 (s, 1H, OH).

^{13}C{1H} NMR (100 MHz, $CDCl_3$): δ 107.1, 113.2, 126.6, 128.3, 129.0, 131.2, 131.7, 137.9, 150.0, 151.5, 200.6.

Anal. calcd. for $C_{13}H_{10}O_4$: C, 67.82; H, 4.38. Found: C, 67.90; H, 4.31.

phenyl(pyridin-4-yl)methanone 3r

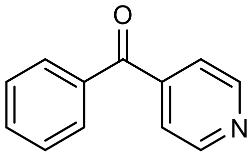

The title compound was prepared starting from an amide **1a** (121 mg, 1 mmol, 1 equiv.), pyrylium tetrafluoroborate **2** (185 mg, 1.1 mmol, 1.1 equiv.), $BaTiO_3$ (933 mg, 4 mmol, 4 equiv.), aryl boronic acid **4q** (160 mg, 1.3 mmol, 1.3 equiv.), DABCO (157 mg, 1.4 mmol, 1.4 equiv.) and 1,4-dioxane (0.25 mL). Yellowish solid, mp 69-71 °C. **^{1}H NMR** (400 MHz, $CDCl_3$): δ 7.51 (t, 2H, J = 7.6 Hz, CH_{Ar}), 7.56 (d, 2H, J = 5.8 Hz, CH_{Ar}), 7.64 (t, 1H, J = 7.5 Hz, CH_{Ar}), 7.81 (d, 2H, J = 7.1 Hz, CH_{Ar}), 8.80 (d, 2H, J = 5.8 Hz, CH_{Ar}).

^{13}C{1H} NMR (100 MHz, $CDCl_3$): δ 122.8, 128.6, 130.1, 133.5, 135.9, 144.6, 150.6, 195.1.

Anal. calcd. for $C_{12}H_9ON$: C, 78.67; H, 4.95; N, 7.65. Found: C, 78.77; H, 5.02; N, 7.58.

(4-bromophenyl)(4-(tert-butyl)phenyl)methanone 3s

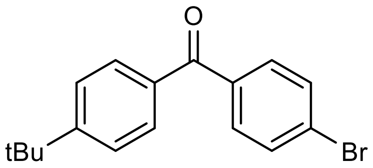

The title compound was prepared starting from an amide **1n** (177 mg, 1 mmol, 1 equiv.), pyrylium tetrafluoroborate **2** (185 mg, 1.1 mmol, 1.1 equiv.), $BaTiO_3$ (933 mg, 4 mmol, 4 equiv.), aryl boronic acid **4m** (260 mg, 1.3 mmol, 1.3 equiv.), DABCO (157 mg, 1.4 mmol, 1.4 equiv.) and 1,4-dioxane (0.25 mL). The purification was accomplished by column chromatography on silica gel to provide the desired **3s** (272 mg, 0.86 mmol, 86%).
Yellowish solid, mp 126-127 °C. **^{1}H NMR** (400 MHz, $CDCl_3$): δ 1.37 (s, 9H, $C(CH_3)_3$), 7.51 (d, 2H, *J* = 7.8 Hz, CH_{Ar}), 7.62 (d, 2H, *J* = 7.8 Hz, CH_{Ar}), 7.69 (d, 2H, *J* = 8.2 Hz, CH_{Ar}), 7.74 (d, 2H, *J* = 8.2 Hz, CH_{Ar}).
^{13}C{1H} NMR (100 MHz, $CDCl_3$): δ 31.1, 35.2, 125.4, 127.2, 130, 131.5, 134.4, 136.7, 156.5, 195.3.
MS (GC, 70eV): m/z (%) = 318 (30), 316 (M^+, 31), 300 (100), 183 (26).
Anal. calcd. for $C_{17}H_{17}BrO$: C, 64.37; H, 5.40. Found: C, 64.425; H, 5.29.

(E)-3-(2-nitrophenyl)-1-phenylprop-2-en-1-one 3t

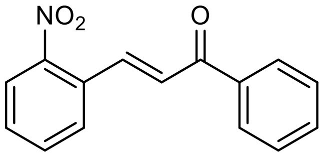

The title compound was prepared starting from an amide **1o** (192 mg, 1 mmol, 1 equiv.), pyrylium tetrafluoroborate **2** (185 mg, 1.1 mmol, 1.1 equiv.), $BaTiO_3$ (933 mg, 4 mmol, 4 equiv.), aryl boronic acid **4a** (158 mg, 1.3 mmol, 1.3 equiv.), DABCO (157 mg, 1.4 mmol, 1.4 equiv.) and 1,4-dioxane (0.25 mL). The purification was accomplished by column chromatography on silica gel to provide the desired **3t** (195 mg, 0.77 mmol, 77%).
Alternatively, the title compound was prepared starting from an amide **1o** (192 mg, 1 mmol, 1 equiv.), pyrylium tetrafluoroborate **2** (185 mg, 1.1 mmol, 1.1 equiv.), $BaTiO_3$ (933 mg, 4 mmol, 4 equiv.), aryl trialkoxysilane **5a** (257 mg, 1.3 mmol, 1.3 equiv.), DABCO (157 mg, 1.4 mmol, 1.4 equiv.) and 1,4-dioxane (0.25 mL). The purification was accomplished by column chromatography on silica gel to provide the desired **3t** (157 mg, 0.62 mmol, 62%).
Light yellow solid, mp 126-127 °C. **^{1}H NMR** (500 MHz, $CDCl_3$): δ 7.33 (d, 1H, ^3J = 15.6 Hz, CH), 7.49 – 7.52 (m, 2H, CH_{Ar}), 7.54 – 7.61 (m, 2H, CH_{Ar}), 7.67 – 7.70 (m, 1H, CH_{Ar}), 7.73 – 7.76 (m, 1H, CH_{Ar}), 8.00 – 8.02 (m, 2H, CH_{Ar}), 8.05 (d, 1H, *J* = 8.3 Hz, CH_{Ar}), 8.13 (d, 1H, ^3J = 15.6 Hz, CH).
^{13}C{1H} NMR (125 MHz, $CDCl_3$): δ 125, 127.3, 128.7, 128.8, 129.3, 130.4, 131.3, 133.2, 133.7, 137.4, 140.2, 148.6, 190.5.
Anal. calcd. for $C_{15}H_{11}NO_3$: C, 71.14; H, 4.38; N, 5.53. Found: C, 71.22; H, 4.31; N, 5.49.

1,3-phenylenebis((4-fluorophenyl)methanone) 3u

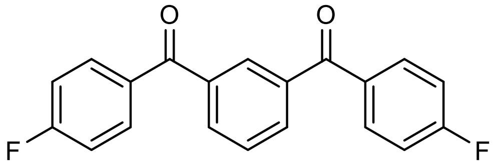

The title compound was prepared starting from an amide **1q** (164 mg, 1 mmol, 1 equiv.), pyrylium tetrafluoroborate **2** (370 mg, 2.2 mmol, 2.2 equiv.), $BaTiO_3$ (1.86 g, 8 mmol, 8 equiv.), aryl boronic acid **4r** (360 mg, 2.6 mmol, 2.6 equiv.), DABCO (314 mg, 2.8 mmol, 2.84 equiv.) and 1,4-dioxane (0.4 mL). The purification was accomplished by column chromatography on silica gel to provide the desired **3u** (267 mg, 0.83 mmol, 83%).
Alternatively, the title compound was prepared starting from an amide **1q** (164 mg, 1 mmol, 1 equiv.), pyrylium tetrafluoroborate **2** (370 mg, 2.2 mmol, 2.2 equiv.), $BaTiO_3$ (1.86 g, 8 mmol, 8 equiv.), aryl trialkoxysilane **5h** (518 mg, 2.6 mmol, 2.6 equiv.), DABCO (314 mg, 2.8 mmol, 2.84 equiv.) and 1,4-dioxane (0.4 mL). The purification was accomplished by column chromatography on silica gel to provide the desired **3u** (180 mg, 0.56 mmol, 56%).
Yellow solid, mp 181-182 °C. **^{1}H NMR** (400 MHz, $CDCl_3$): δ 7.17 (t, 4H, *J* = 8.8 Hz, CH_{Ar}), 7.64 (t, 1H, *J* = 7.7 Hz, CH_{Ar}), 7.84 – 7.88 (m, 4H, CH_{Ar}), 7.99 (dd, 2H, *J* = 7.7 Hz, *J* = 1.5 Hz, CH_{Ar}), 8.12 (s, 1H, CH_{Ar}).
^{13}C{1H} NMR (100 MHz, $CDCl_3$): δ 115.6, 115.8, 128.6, 130.8, 132.6, 132.7, 133.1, 133.2, 133.3, 164.4, 166.69, 194.2.
MS (GC, 70eV): m/z (%) = 322 (M^+, 40), 227 (23), 123 (100).
Anal. calcd. for $C_{20}H_{12}F_2O_2$: C, 74.53; H, 3.75. Found: C, 74.61; H, 3.88.

cyclopropyl(4-methoxyphenyl)methanone 3v

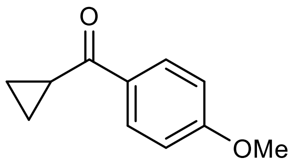

The title compound was prepared starting from an amide **1r** (85 mg, 1 mmol, 1 equiv.), pyrylium tetrafluoroborate **2** (185 mg, 1.1 mmol, 1.1 equiv.), $BaTiO_3$ (933 mg, 4 mmol, 4 equiv.), aryl boronic acid **4s** (197 mg, 1.3 mmol, 1.3 equiv.), DABCO (157 mg, 1.4 mmol, 1.4 equiv.) and 1,4-dioxane (0.25 mL). The purification was accomplished by column chromatography on silica gel to provide the desired **3v** (148 mg, 0.84 mmol, 84%).
Alternatively, the title compound was prepared starting from an amide **1r** (85 mg, 1 mmol, 1 equiv.), pyrylium tetrafluoroborate **2** (185 mg, 1.1 mmol, 1.1 equiv.), $BaTiO_3$ (933 mg, 4 mmol, 4 equiv.), aryl trialkoxysilane **5i** (296 mg, 1.3 mmol, 1.3 equiv.), DABCO (157 mg, 1.4 mmol, 1.4 equiv.) and 1,4-dioxane (0.25 mL). The purification was accomplished by column chromatography on silica gel to provide the desired **3v** (91 mg, 0.52 mmol, 52%).
White solid, mp 40-41 °C. **^{1}H NMR** (400 MHz, $CDCl_3$): δ 0.97 – 1.00 (m, 2H, CH_2-Cyclopropyl), 1.19 – 1.21 (m, 2H, CH_2-Cyclopropyl), 2.61 – 2.63 (m, 1H, CH-Cyclopropyl), 3.86 (s, 3H, Ome), 6.94 (d, 2H, *J* = 8.0 Hz, CH_{Ar}), 8.00 (d, 2H, *J* = 9.1 Hz, CH_{Ar}).
^{13}C{1H} NMR (100 MHz, $CDCl_3$): δ 11.2, 16.6, 55.4, 113.6, 130.2, 131.0, 163.2, 199.0.
MS (GC, 70eV): m/z (%) = 176 (M^+, 35), 135 (100).
Anal. calcd. for $C_{14}H_{12}O_2$: C, 74.98; H, 6.86. Found: C, 75.06; H, 6.73.

1-(4-methoxy-3-(trifluoromethyl)phenyl)ethan-1-one 3w

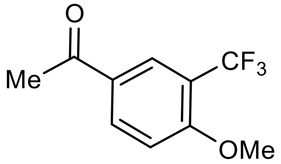

The title compound was prepared starting from an amide **1s** (59 mg, 1 mmol, 1 equiv.), pyrylium tetrafluoroborate **2** (185 mg, 1.1 mmol, 1.1 equiv.), $BaTiO_3$ (933 mg, 4 mmol, 4 equiv.), aryl boronic acid **4t** (286 mg, 1.3 mmol, 1.3 equiv.), DABCO (157 mg, 1.4 mmol, 1.4 equiv.) and 1,4-dioxane (0.25 mL). The purification was accomplished by column chromatography on silica gel to provide the desired **3w** (192 mg, 0.88 mmol, 88%).

Colourless kiquid **^{1}H NMR** (400 MHz, $CDCl_3$): δ 2.58 (s, 3H, $C(O)CH_3$), 3.98 (s, 3H, OCH_3), 7.06 (d, 1H, J = 8.7 Hz, CH_{Ar}), 8.13 (dd, 1H, J = 8.7 Hz, J = 2.1 Hz, CH_{Ar}), 8.19 (d, 1H, J = 2.1 Hz, CH_{Ar}).

^{19}F NMR (376 MHz, $CDCl_3$): δ -62.9 (s, CF_3).

^{13}C{1H} NMR (100 MHz, $CDCl_3$): δ 26.3, 56.3, 111.6, 118.8 (q, J_{CF} = 32 Hz), 123.1 (q, $^{1}J_{CF}$ = 273 Hz), 127.9 (q, J_{CF} = 5.2 Hz), 129.5, 133.9, 161 (q, J_{CF} = 1.5 Hz), 195.6.

MS (GC, 70eV): m/z (%) = 218 (M^+, 24), 203 (100), 127 (19).

Anal. calcd. for $C_{10}H_9F_3O_2$: C, 55.05; H, 4.16. Found: C, 55.19; H, 4.03.

1-(4-hydroxyphenyl)butan-1-one 3x

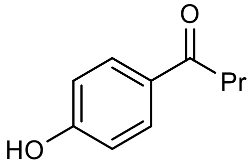

The title compound was prepared starting from an amide **1t** (87 mg, 1 mmol, 1 equiv.), pyrylium tetrafluoroborate **2** (185 mg, 1.1 mmol, 1.1 equiv.), $BaTiO_3$ (933 mg, 4 mmol, 4 equiv.), aryl boronic acid **4o** (179 mg, 1.3 mmol, 1.3 equiv.), DABCO (157 mg, 1.4 mmol, 1.4 equiv.) and 1,4-dioxane (0.25 mL). The purification was accomplished by column chromatography on silica gel to provide the desired **3x** (149 mg, 0.91 mmol, 91%). The gram scale synthesis was performed on 10 mmol of starting amide **1t** and the product **3x** was prepared 88% in yield (1.44 g, 8.8 mmol).

Alternatively, the title compound was prepared starting from an amide **1t** (87 mg, 1 mmol, 1 equiv.), pyrylium tetrafluoroborate **2** (185 mg, 1.1 mmol, 1.1 equiv.), $BaTiO_3$ (933 mg, 4 mmol, 4 equiv.), aryl trialkoxysilane **5j** (278 mg, 1.3 mmol, 1.3 equiv.), DABCO (157 mg, 1.4 mmol, 1.4 equiv.) and 1,4-dioxane (0.25 mL). The purification was accomplished by column chromatography on silica gel to provide the desired **3x** (98 mg, 0.60 mmol, 60%).

Pale yellow solid, mp 90-91 ^{o}C. **^{1}H NMR** (400 MHz, $CDCl_3$): δ 0.99 (t, 3H, J = 7.2 Hz, Me), 1.76 (m, 2H, CH_2), 2.93 (t, 2H, J = 7.2 Hz, CH_2), 6.99 (d, 2H, J = 8.9 Hz, CH_{Ar}), 7.92 (d, 2H, J = 8.9 Hz, CH_{Ar}), 8.71 (s, 1H, OH).

^{13}C{1H} NMR (100 MHz, $CDCl_3$): δ 13.8, 18.3, 40.2, 115.6, 129.0, 131.0, 161.6, 201.7.
MS (GC, 70eV): m/z (%) = 164 (M^+, 14), 121 (100).
Anal. calcd. for $C_{10}H_{12}O_2$: C, 73.15; H, 7.37. Found: C, 73.19; H, 7.42.

3-(3,5-bis(trifluoromethyl)benzoyl)-6-methyl-4H-chromen-4-one 3y

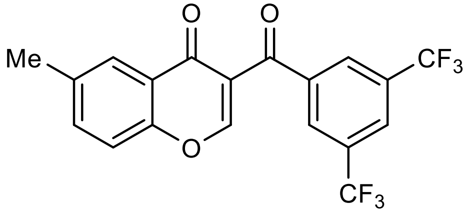

The title compound was prepared starting from an amide **1u** (203 mg, 1 mmol, 1 equiv.), pyrylium tetrafluoroborate **2** (185 mg, 1.1 mmol, 1.1 equiv.), $BaTiO_3$ (933 mg, 4 mmol, 4 equiv.), aryl boronic acid **4h** (335 mg, 1.3 mmol, 1.3 equiv.), DABCO (157 mg, 1.4 mmol, 1.4 equiv.) and 1,4-dioxane (0.25 mL). The purification was accomplished by column chromatography on silica gel to provide the desired **3y** (312 mg, 0.78 mmol, 78%).
Yellowish solid, mp 168-169 °C. **^{1}H NMR** (500 MHz, $CDCl_3$): δ 2.49 (s, 3H, Me), 7.48 (d, 1H, J = 8.3 Hz, CH_{Ar}), 7.58 (dd, 1H, J = 8.5 Hz, J = 1.9 Hz, CH_{Ar}), 8.01 (br.s, 1H, CH_{Ar}), 8.07 (s, 1H, CH_{Ar}), 8.22 (s, 2H, CH_{Ar}), 8.46 (s, 1H, CH_{Ar}).
^{13}C{1H} NMR (125 MHz, $CDCl_3$): δ 21.0, 118,2, 122,9 (₽, $^1J_{CF}$ = 270.1 Hz), 123.4, 124.5, 125.8, 126.2 (m), 129.4, 131.8 (q, J_{CF} = 33.8 Hz), 136.1, 136.9, 139.1, 154.3, 160.6, 174.5, 190.0.
MS (GC, 70eV): m/z (%) = 400 (M^+, 54), 371 (100), 303 (28), 213 (27), 187 (25), 135 (43).
Anal. calcd. for $C_{19}H_{10}F_6O_3$: C, 57.01; H, 2.52. Found: C, 57.12; H, 2.46.

6-fluoro-3-(thiophene-2-carbonyl)-4H-chromen-4-one 3z

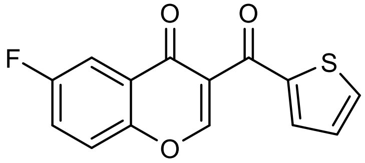

The title compound was prepared starting from an amide **1v** (207 mg, 1 mmol, 1 equiv.), pyrylium tetrafluoroborate **2** (185 mg, 1.1 mmol, 1.1 equiv.), $BaTiO_3$ (933 mg, 4 mmol, 4 equiv.), aryl boronic acid **4v** (166 mg, 1.2 mmol, 1.2 equiv.), DABCO (157 mg, 1.4 mmol, 1.4 equiv.) and 1,4-dioxane (0.25 mL). The purification was accomplished by column chromatography on silica gel to provide the desired **3z** (224 mg, 0.82 mmol, 82%).
Yellowish solid, mp 133-134 °C.**^{1}H NMR** (500 MHz, $CDCl_3$): δ 7.14 (br.s, 1H, CH_{Ar}), 7.46 (br.s, 1H, CH_{Ar}), 7.55 (br.s, 1H, CH_{Ar}), 7.72 (m, 2H, CH_{Ar}), 7.19 (s, 2H, CH_{Ar}), 8.31 (s, 1H, CH_{Ar}).

^{13}C{1H} NMR (125 MHz, $CDCl_3$): δ 111.5 (d, J_{CF} = 24.2 Hz), 120.6 (d, J_{CF} = 8.2 Hz), 122.7 (d, J_{CF} = 25.5 Hz), 124.6, 126.3 (d, J_{CF} = 7.9 Hz), 128.3, 135.4, 135.5, 143.6, 152.2, 158.3, 160.1 (d, $^1J_{CF}$ = 249 Hz), 173.6, 182.4.
MS (GC, 70eV): m/z (%) = 274 (M^+, 54), 246 (100), 191 (28), 138 (27), 111 (25).
Anal. calcd. for $C_{14}H_7FO_3S$: C, 61.31; H, 2.57. Found: C, 61.39; H, 2.63.

3-(2,4-difluorobenzoyl)-4H-chromen-4-one 3aa

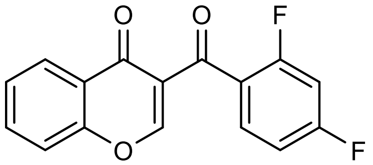

The title compound was prepared starting from an amide **1w** (189 mg, 1 mmol, 1 equiv.), pyrylium tetrafluoroborate **2** (185 mg, 1.1 mmol, 1.1 equiv.), $BaTiO_3$ (933 mg, 4 mmol, 4 equiv.), aryl boronic acid **4u** (205mg, 1.3 mmol, 1.3 equiv.), DABCO (157 mg, 1.4 mmol, 1.4 equiv.) and 1,4-dioxane (0.25 mL). The purification was accomplished by column chromatography on silica gel to provide the desired **3aa** (234 mg, 0.82 mmol, 82%).
Alternatively, the title compound was prepared starting from an amide **1p** (157 mg, 1 mmol, 1 equiv.), pyrylium tetrafluoroborate **2** (185 mg, 1.1 mmol, 1.1 equiv.), $BaTiO_3$ (933 mg, 4 mmol, 4 equiv.), aryl boronic acid **4w** (247 mg, 1.3 mmol, 1.3 equiv.), DABCO (157 mg, 1.4 mmol, 1.4 equiv.) and 1,4-dioxane (0.25 mL). The purification was accomplished by column chromatography on silica gel to provide the desired **3aa** (203 mg, 0.71 mmol, 71%).
Yellowish solid, mp 127-128 ^oC. **^{1}H NMR** (500 MHz, $CDCl_3$): δ 6.82 (ddd, 1H, J = 10.6 Hz, J = 8.5 Hz, J = 2.3 Hz, CH_{Ar}), 6.97 – 7.01 (m, 1H, CH_{Ar}), 7.44 – 7.47 (m, 1H, CH_{Ar}), 7.52 – 7.54 (m, 1H, CH_{Ar}), 7.71 – 7.74 (m, 1H, CH_{Ar}), 7.76 – 7.80 (m, 1H, CH_{Ar}), 8.21 (dd, 1H, J = 8 Hz, J = 1.7 Hz, CH_{Ar}), 8.43 (s, 1H, CH_{Ar}).
^{13}C{1H} NMR (125 MHz, $CDCl_3$): δ 104.4 (t, J_{CF} = 26 Hz), 112 (dd, J_{CF} = 22 Hz, J_{CF} = 3.2 Hz), 118.4, 124 (dd, J_{CF} = 12.5 Hz, J_{CF} = 3.5 Hz), 124.9, 125.7, 126.3, 126.4, 132.3 (dd, J_{CF} = 10.6 Hz, J_{CF} = 3.5 Hz), 134.5, 156, 159.6, 162.1 (dd, $^1J_{CF}$ = 256 Hz, J_{CF} = 12.1 Hz), 165.8 (dd, $^1J_{CF}$ = 256 Hz, J_{CF} = 12.1 Hz), 174.5 (d, J_{CF} = 2 Hz), 187.4.
MS (GC, 70eV): m/z (%) = 286 (M^+, 95), 267 (49), 257 (100), 239 (60), 173 (44), 141 (72), 113 (59).
Anal. calcd. for $C_{16}H_8F_2O_3$: C, 67.14; H, 2.82. Found: C, 67.31; H, 2.93.

N-cyclohexyl-3,5-difluorobenzamide 7a IVA

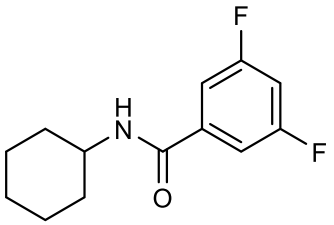

The title compound was prepared starting from a urea **6a** (189 mg, 1 mmol, 1 equiv.), pyrylium tetrafluoroborate **2** (185 mg, 1.1 mmol, 1.1 equiv.), $BaTiO_3$ (933 mg, 4 mmol, 4 equiv.), aryl boronic acid **4e** (205 mg, 1.3 mmol, 1.3 equiv.), DABCO (157 mg, 1.4 mmol, 1.4 equiv.) and 1,4-dioxane (0.25 mL). The purification was accomplished by column chromatography on silica gel to provide the desired **7a** (181 mg, 0.76 mmol, 76%). The gram scale synthesis was performed on 10 mmol of starting urea **6a** and the product **7a** was prepared 71% in yield (1.70 g, 7.1 mmol).

White solid, mp 138-139 °C. **^{1}H NMR** (500 MHz, $CDCl_3$): δ 1.12 (m, 1H, Cyclohexyl), 1.26 – 1.31 (m, 4H, cyclohexyl), 1.58 (m, 1H, Cyclohexyl), 1.71 – 1.81 (m, 4H, Cyclohexyl), 3.73 (m, 1H, CH-Cyclohexyl), 7.40 (tt, 1H, *J* = 9.0 Hz, *J* = 2.4 Hz, CH_{Ar}), 7.54 (dq, 2H, *J* = 8.4 Hz, *J* = 2.1 Hz, CH_{Ar}), 8.35 (d, 1H, *J* = 8.4 Hz, NH).

^{13}C{1H} NMR (125 MHz, $CDCl_3$): δ 24.5, 25.2, 32.3, 48.7, 106.4 (t, J_{CF} = 26.6 Hz), 110.5 (t, J_{CF} = 6.1 Hz), 110.7 (t, J_{CF} = 6.4 Hz), 138.3 (t, J_{CF} = 8.1 Hz), 162.2 (dd, $^1J_{CF}$ = 246.8 Hz, J_{CF} = 11.5 Hz),

MS (GC, 70eV): m/z (%) = 239 (M^+, 30), 158 (88), 141 (100), 113 (41).

Anal. calcd. for $C_{13}H_{15}F_2ON$: C, 65.36; H, 6.32; N, 5.85. Found: C, 65.29; H, 6.19; N, 5.93.

N-benzyl-[1,1'-biphenyl]-4-carboxamide 7b

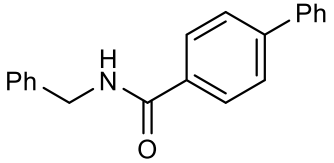

The title compound was prepared starting from a urea **6b** (150 mg, 1 mmol, 1 equiv.), pyrylium tetrafluoroborate **2** (185 mg, 1.1 mmol, 1.1 equiv.), $BaTiO_3$ (933 mg, 4 mmol, 4 equiv.), aryl boronic acid **4x** (257 mg, 1.3 mmol, 1.3 equiv.), DABCO (157 mg, 1.4 mmol, 1.4 equiv.) and 1,4-dioxane (0.25 mL). The purification was accomplished by column chromatography on silica gel to provide the desired **7b** (227 mg, 0.79 mmol, 79%).

White solid, mp 181-182 °C. **^{1}H NMR** (500 MHz, DMSO-*d$_6$*): δ 4.53 (d, 2H, *J* = 6.0 Hz, CH_2), 7.25 (m, 1H, CH_{Ar}), 7.32 – 7.36 (m, 4H, CH_{Ar}), 7.40 (t, 1H, *J* = 7.5 Hz, CH_{Ar}), 7.49 (t, 2H, *J* = 7.5 Hz, CH_{Ar}), 7.72 (d, 2H, *J* = 7.4 Hz, CH_{Ar}), 7.79 (d, 2H, *J* = 8.5 Hz, CH_{Ar}), 8.05 (d, 2H, *J* = 8.3 Hz, CH_{Ar}), 9.15 (t, 1H, *J* = 6.0 Hz, NH).

^{13}C{1H} NMR (125 MHz, DMSO-*d$_6$*): δ 42.6, 126.6, 126.7, 126.9, 127.2, 128.0, 128.1, 128.3, 129.0, 133.1, 139.2, 139.2, 139.7, 142.8.

MS (GC, 70eV): m/z (%) = 287 (M^+, 71), 181 (100), 152 (51).

Anal. calcd. for $C_{20}H_{17}ON$: C, 83.59; H, 5.96; N, 4.87. Found: C, 83.62; H, 6.02; N, 4.77.

N-(4-iodophenyl)-2-methoxybenzamide 7c

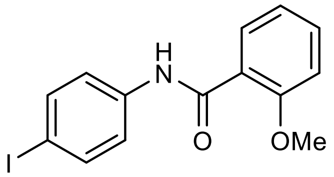

The title compound was prepared starting from a urea **6c** (262 mg, 1 mmol, 1 equiv.), pyrylium tetrafluoroborate **2** (185 mg, 1.1 mmol, 1.1 equiv.), $BaTiO_3$ (933 mg, 4 mmol, 4 equiv.), aryl boronic acid **4y** (197 mg, 1.3 mmol, 1.3 equiv.), DABCO (157 mg, 1.4 mmol, 1.4 equiv.) and 1,4-dioxane (0.25 mL). The purification was accomplished by column chromatography on silica gel to provide the desired **7c** (261 mg, 0.74 mmol, 74%).
White solid, mp 125-126 ^oC. **^{1}H NMR** (500 MHz, $CDCl_3$): δ 4.01 (s, 3H, OMe), 6.98 (d, 1H, J = 8.4 Hz, CH_{Ar}), 7.10 (t, 1H, J = 7.6 Hz, CH_{Ar}), 7.44-7.49 (m, 3H, CH_{Ar}), 7.61 (d, 2H, J = 8.4 Hz, CH_{Ar}), 8.22 (dd, 1H, J = 7.8 Hz, J = 1.5 Hz, CH_{Ar}), 9.80 (s, 1H, NH).
^{13}C{1H} NMR (125 MHz, $CDCl_3$): δ 56.2, 87.1, 114.4, 121.2, 121.6, 122.1, 132.3, 133.4, 137.7, 138.1, 157.0, 163.2.
MS (GC, 70eV): m/z (%) = 353 (M^+, 44), 135 (100).
Anal. calcd. for $C_{16}H_{12}O_2NI$: C, 47.61; H, 3.43; N, 3.97. Found: C, 47.52; H, 3.35; N, 4.01.

N-(3,5-dimethoxyphenyl)-3,4-difluorobenzamide 7d

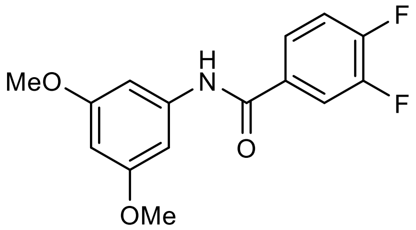

The title compound was prepared starting from a urea **6d** (196 mg, 1 mmol, 1 equiv.), pyrylium tetrafluoroborate **2** (185 mg, 1.1 mmol, 1.1 equiv.), $BaTiO_3$ (933 mg, 4 mmol, 4 equiv.), aryl boronic acid **4z** (205 mg, 1.3 mmol, 1.3 equiv.), DABCO (157 mg, 1.4 mmol, 1.4 equiv.) and 1,4-dioxane (0.25 mL). The purification was accomplished by column chromatography on silica gel to provide the desired **7d** (231 mg, 0.79 mmol, 79%). The gram scale synthesis was performed on 10 mmol of starting urea **6d** and the product **7d** was prepared 72% in yield (2.11 g, 7.2 mmol).
Yellowish solid, mp 112-113 ^oC. **^{1}H NMR** (500 MHz, DMSO-d_6): δ 3.70 (s, 6H, 2xOMe), 6.24 (t, 1H, J = 2.1 Hz, CH_{Ar}), 7.02 (d, 2H, J = 2.1 Hz, CH_{Ar}), 7.52-7.58 (m, 1H, CH_{Ar}), 7.81 (m, 1H, CH_{Ar}), 7.95 – 8.00 (m, 1H, CH_{Ar}), 10.17 (s, 1H, NH).
^{13}C{1H} NMR (125 MHz, $CDCl_3$): δ 55.1, 96.0, 98.6, 117.4 (dd, J_{CF} = 54.1 Hz, J_{CF} = 18.6 Hz), 125.2 (m), 132.2 (m), 140.5, 149.2 (dd, $^1J_{CF}$ = 245.6 Hz, J_{CF} = 14.5 Hz), 151.4(dd, $^1J_{CF}$ = 253.5 Hz, J_{CF} = 10.5 Hz), 160.4, 163.2.

MS (GC, 70eV): m/z (%) = 293 (M^+, 48), 141 (100), 113 (39).
Anal. calcd. for $C_{15}H_{13}O_3NF$: C, 61.43; H, 4.47; N, 4.78. Found: C, 61.53; H, 4.41; N, 4.89.

(3-hydroxyphenyl)(piperidin-1-yl)methanone 7e

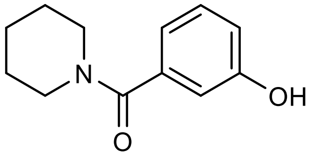

The title compound was prepared starting from a urea **6e** (128 mg, 1 mmol, 1 equiv.), pyrylium tetrafluoroborate **2** (185 mg, 1.1 mmol, 1.1 equiv.), $BaTiO_3$ (933 mg, 4 mmol, 4 equiv.), aryl boronic acid **4aa** (179 mg, 1.3 mmol, 1.3 equiv.), DABCO (157 mg, 1.4 mmol, 1.4 equiv.) and 1,4-dioxane (0.25 mL). The purification was accomplished by column chromatography on silica gel to provide the desired **7e** (147 mg, 0.72 mmol, 72%).
Yellowish solid, mp 120-122 °C. **^{1}H NMR** (400 MHz, DMSO-d_6): δ 1.49 (s, 2H, piperidyl), 1.66 (s, 2H, piperidyl), 3.34 (s, 2H, piperidyl), 3.70 (s, 2H, piperidyl), 6.79 (t, 2H, J = 6.6 Hz, CH_{Ar}), 6.95 (s, 1H, CH_{Ar}), 7.14-7.18 (m, 1H, CH_{Ar}), 8.22 (s, 1H, OH).
^{13}C{1H} NMR (100 MHz, $CDCl_3$): δ 24.5, 26.6, 26.5, 43.3, 48.9, 114.6, 117.2, 117.5, 129.4, 136.6, 157.1, 170.8.
MS (GC, 70eV): m/z (%) = 205 (M^+, 39), 204 (100), 121 (76).
Anal. calcd. for $C_{12}H_{15}O_2N$: C, 70.22; H, 7.37; N, 6.82. Found: C, 70.28; H, 7.31; N, 6.78.

N,N-dibenzyl-4-fluorobenzamide 8

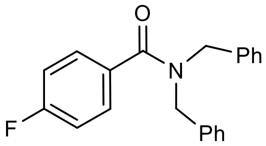

The title compound was prepared starting from the freshly prepared pyridinium salt (289 mg, 1 mmol, 1 equiv.), pyrylium tetrafluoroborate **2** (185 mg, 1.1 mmol, 1.1 equiv.), $BaTiO_3$ (933 mg, 4 mmol, 4 equiv.), freshly sublimated DABCO (157 mg, 1.4 mmol, 1.4 equiv.) and freshly distilled 1,4-dioxane (0.25 mL). Afterwords, dibenzylamine (296 mg, 1.5 mmol, 1.5 equiv.) The purification was accomplished by column chromatography on silica gel to provide the desired **8** (258 mg, 0.81 mmol, 81%).
Yellowish solid, mp 86-87 °C. **^{1}H NMR** (500 MHz, DMSO-d_6): δ 4.40 (s, 2H, CH_2), 4.58 (s, 2H, CH_2), 7.15 – 7.36 (m, 12H, CH_{Ar}), 7.52 – 7.55 (m, 2H, CH_{Ar}).
^{13}C{1H} NMR (125 MHz, DMSO-d_6): δ 47.1, 51.6, 115.5 (d, J_{CF} = 21.7 Hz), 126.8 (m), 127.3 (m), 127.6 (m), 128.7 (m), 129.0 (d, J_{CF} = 8.3 Hz), 132.5 (m), 136.5 (m), 137.0 (m), 162.5 (d, $^1J_{CF}$ = 247.1 Hz), 170.3.
MS (GC, 70eV): m/z (%) = 319 (M^+, 3), 228 (39), 123 (100).
Anal. calcd. for $C_{21}H_{18}ONF$: C, 78.98; H, 5.68; N, 4.39. Found: C, 79.11; H, 5.79; N, 4.23.

(C) Copies ^{1}H and ^{13}C NMR spectra

Compound 3a

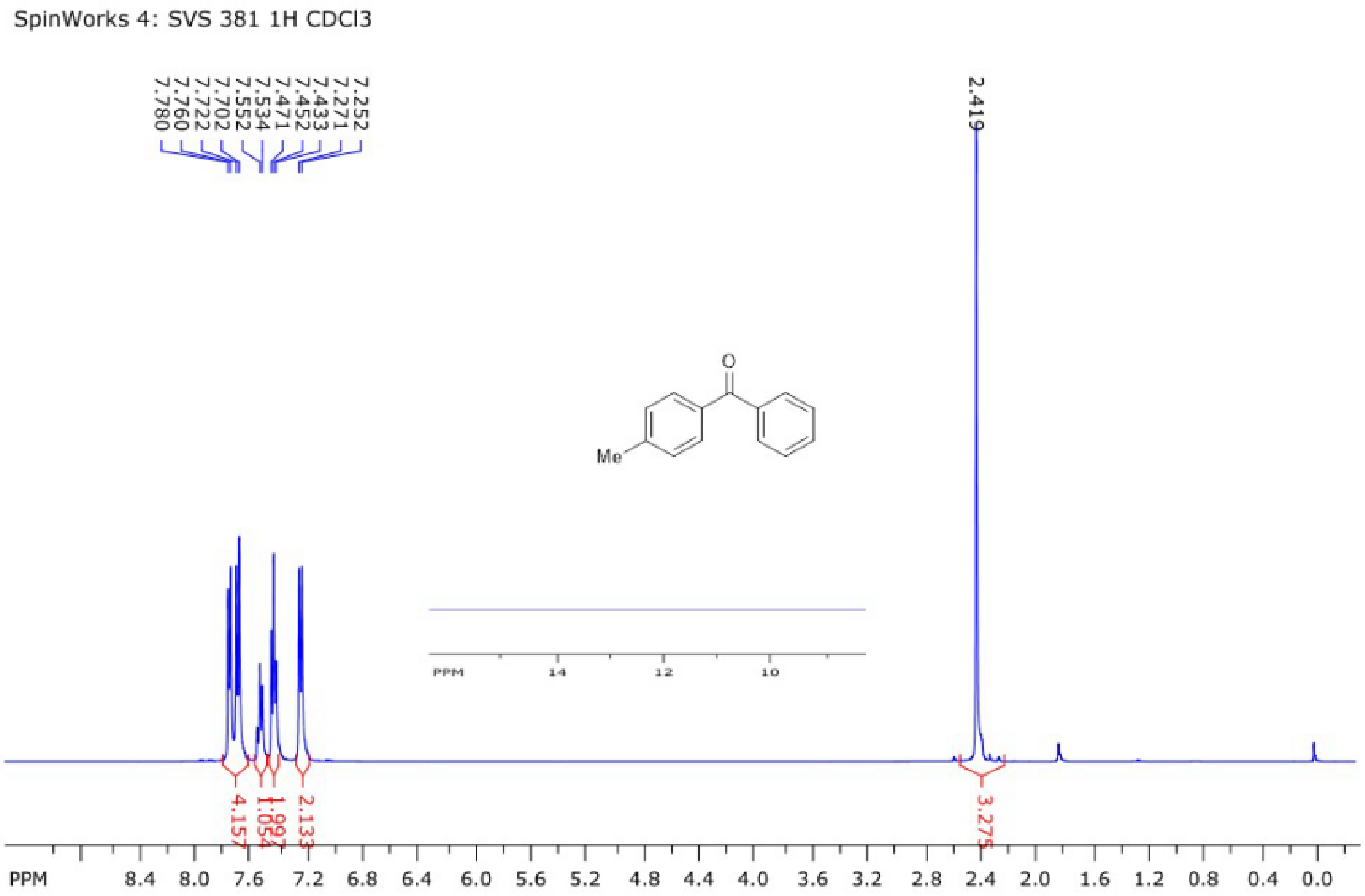

file: D:\NAPO\NMR\JELA\nmr\jn-381\2\fid expt: <zg30>
transmitter freq.: 400.132471 MHz
time domain size: 65536 points
width: 8196.72 Hz = 20.4850 ppm = 0.125072 Hz/pt
number of scans: 16

freq. of 0 ppm: 400.130014 MHz
processed size: 65536 complex points
LB: 0.300 GF: 0.0000
Hz/cm: 154.867 ppm/cm: 0.38704

Compound 3a

SpinWorks 4:

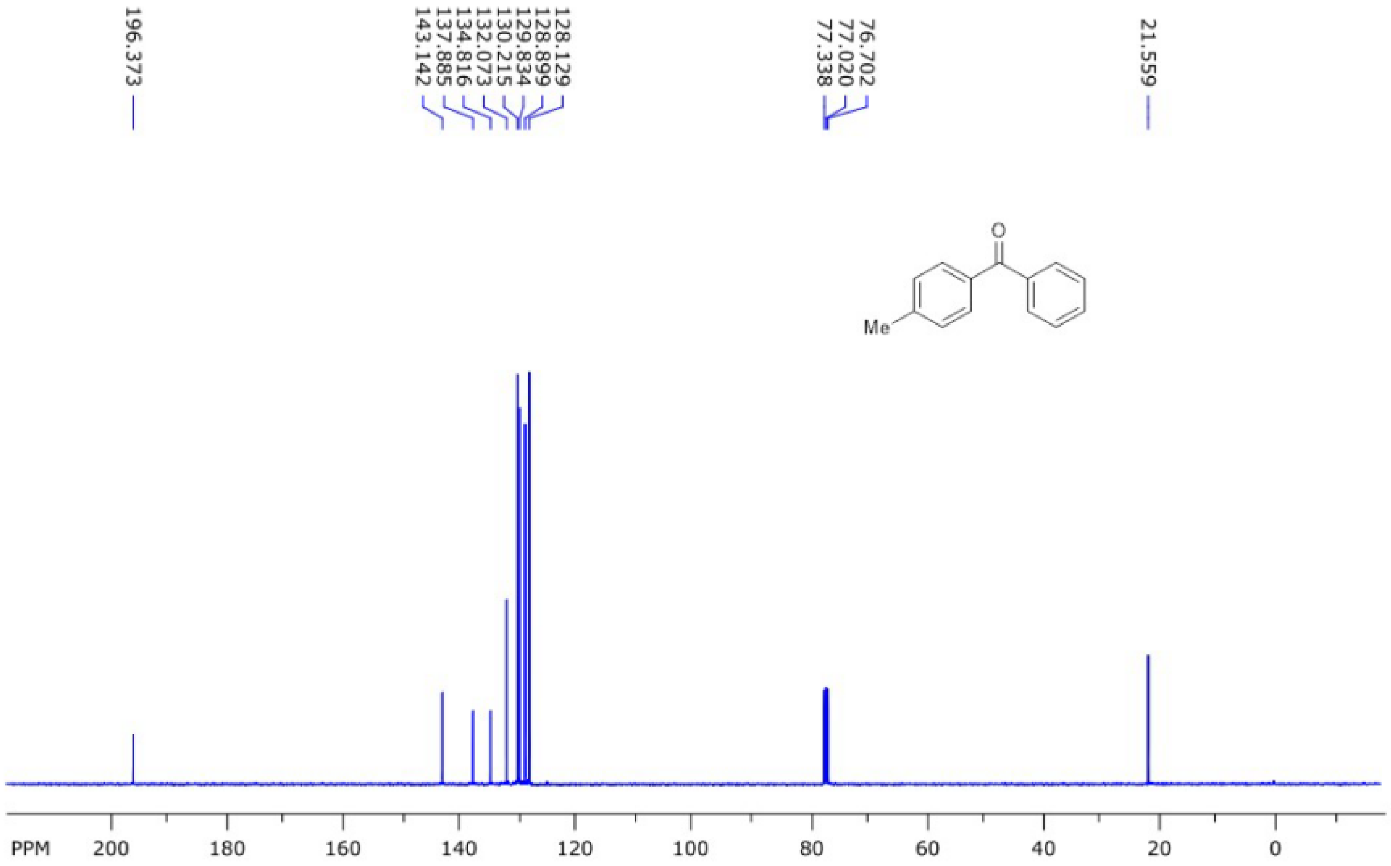

file: D:\NAPO\NMR\JELA\nmr\jn-381\1\fid expt: <zgpg30>
transmitter freq.: 100.622830 MHz
time domain size: 65536 points
width: 23809.52 Hz = 236.6215 ppm = 0.363305 Hz/pt
number of scans: 1100

freq. of 0 ppm: 100.612778 MHz
processed size: 32768 complex points
LB: 1.000 GF: 0.0000
Hz/cm: 952.381 ppm/cm: 9.46486

Compound 3b

SpinWorks 4: SVS 380 1H CDCl3

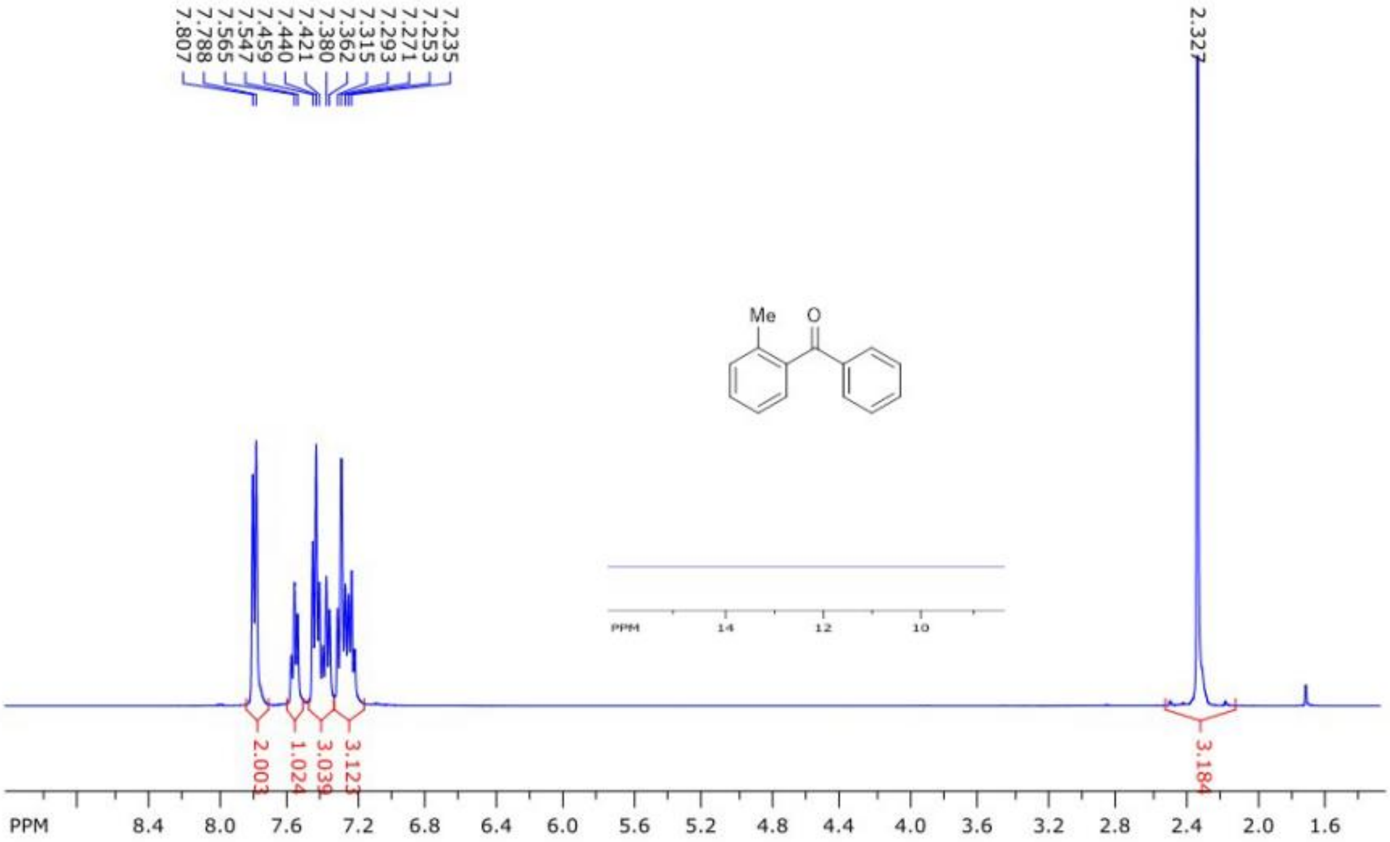

file: D:\NAPO\NMR\JELA\nmr\jn-380\3\fid expt: <zg30>
transmitter freq.: 400.132471 MHz
time domain size: 65536 points
width: 8196.72 Hz = 20.4850 ppm = 0.125072 Hz/pt
number of scans: 16

freq. of 0 ppm: 400.130014 MHz
processed size: 65536 complex points
LB: 0.300 GF: 0.0000
Hz/cm: 127.666 ppm/cm: 0.31906

Compound 3b

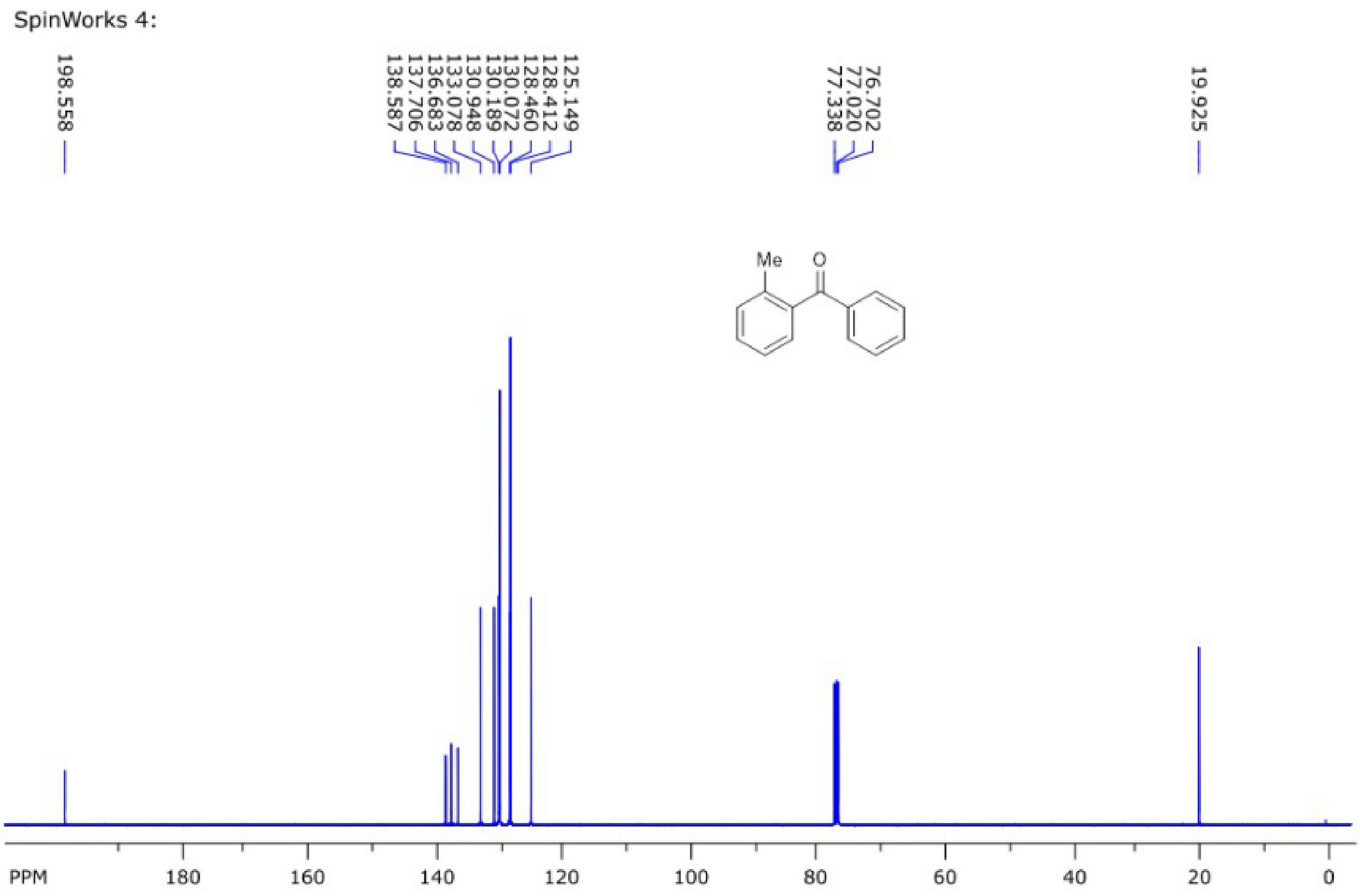

file: D:\NAPO\NMR\JELA\nmr\jn-380\1\fid expt: <zgpg30>
transmitter freq.: 100.622830 MHz
time domain size: 65536 points
width: 23809.52 Hz = 236.6215 ppm = 0.363305 Hz/pt
number of scans: 1024

freq. of 0 ppm: 100.612774 MHz
processed size: 32768 complex points
LB: 1.000 GF: 0.0000
Hz/cm: 853.350 ppm/cm: 8.48068

Compound 3c

SpinWorks 4: IVA 1951 1H CDCl3

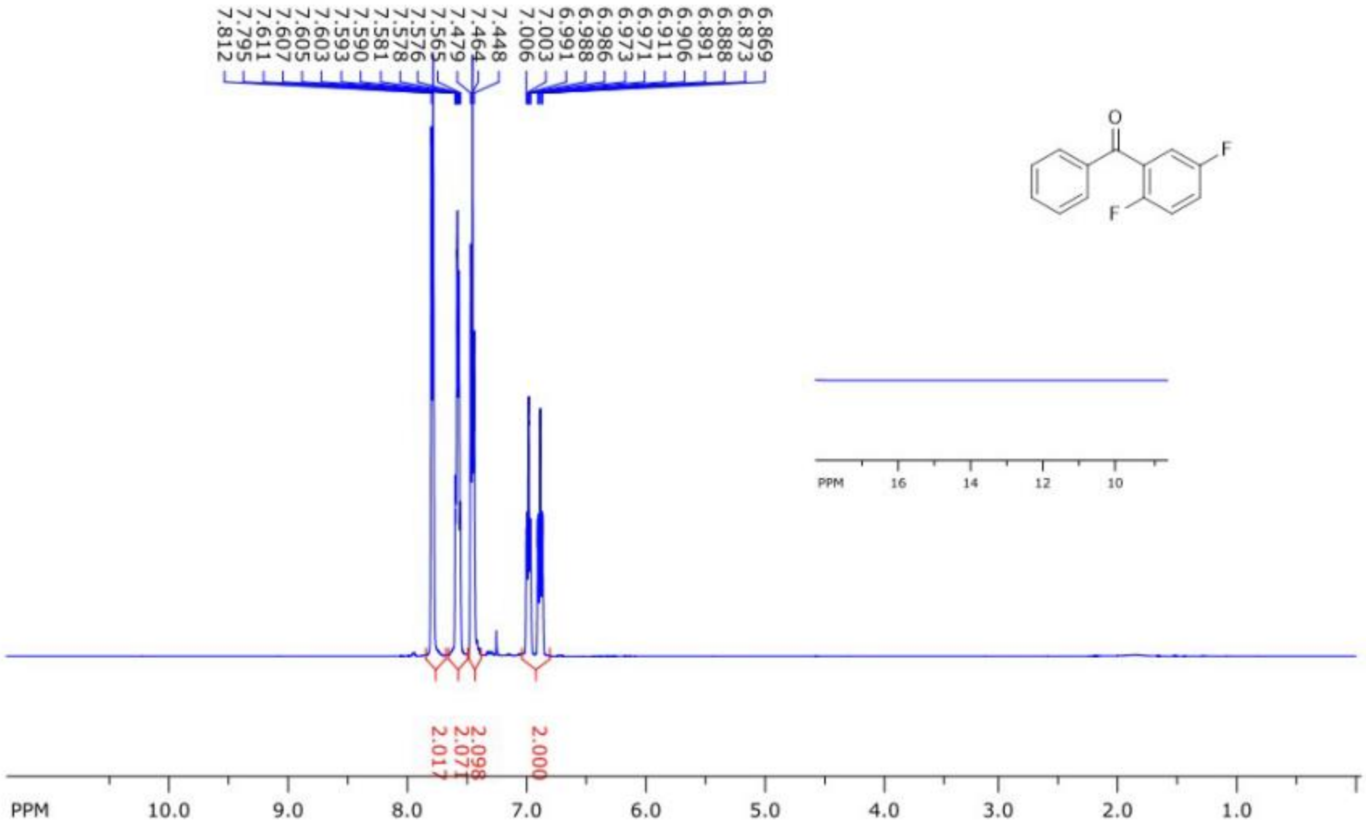

file: D:\NAPO\NMR\500-2\mkr10603\11\fid expt: <zg30>
transmitter freq.: 500.133001 MHz
time domain size: 65536 points
width: 12335.53 Hz = 24.6645 ppm = 0.188225 Hz/pt
number of scans: 24

freq. of 0 ppm: 500.130022 MHz
processed size: 65536 complex points
LB: 0.300 GF: 0.0000
Hz/cm: 228.698 ppm/cm: 0.45728

Compound 3c

SpinWorks 4: IVA 1951 13C CDCL3

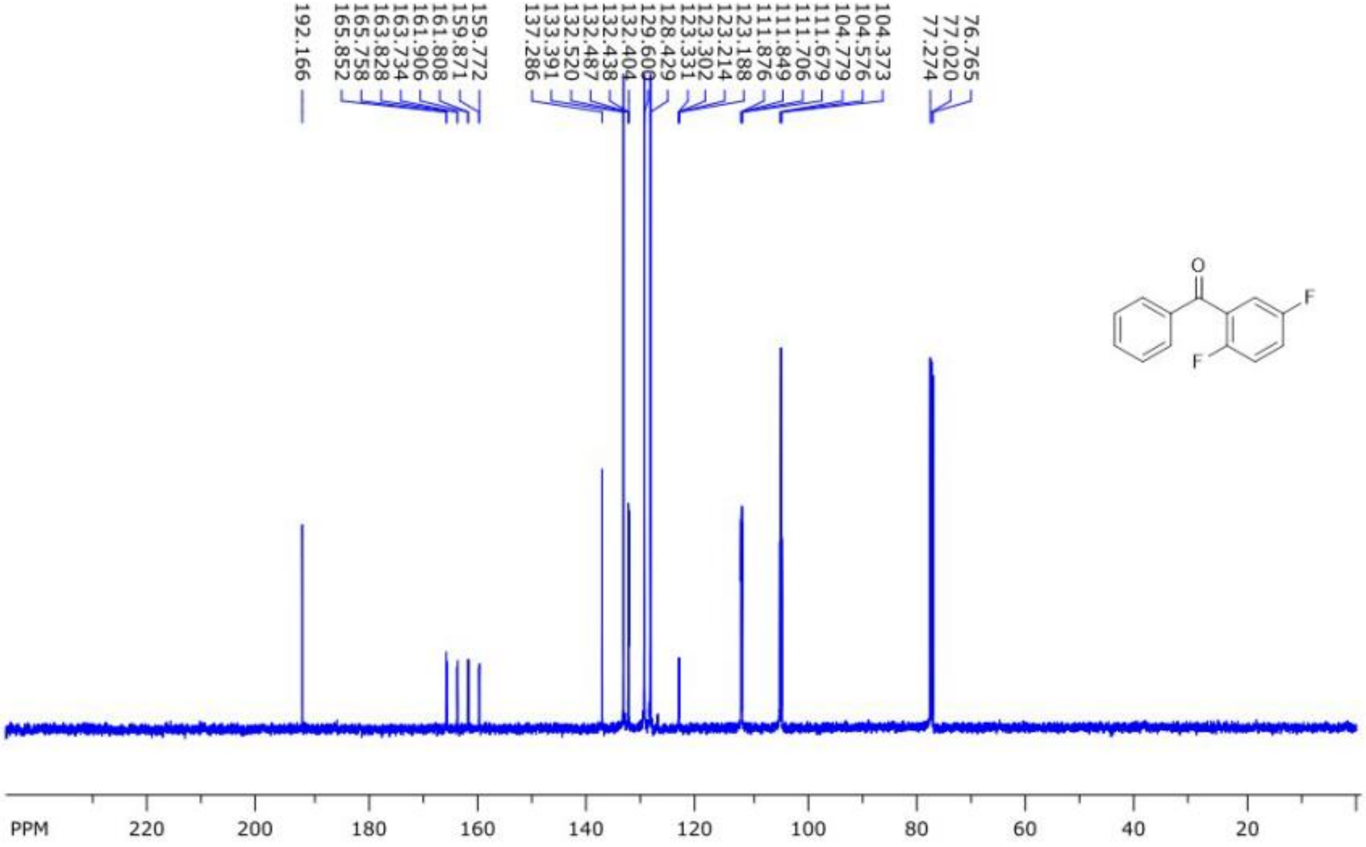

file: D:\NAPO\NMR\500-2\mkr10603\12\fid expt: <zgpg30>
transmitter freq.: 125.772879 MHz
time domain size: 65536 points
width: 36057.69 Hz = 286.6889 ppm = 0.550197 Hz/pt
number of scans: 512

freq. of 0 ppm: 125.757802 MHz
processed size: 32768 complex points
LB: 2.000 GF: 0.0000
Hz/cm: 1242.874 ppm/cm: 9.88189

Compound 3d

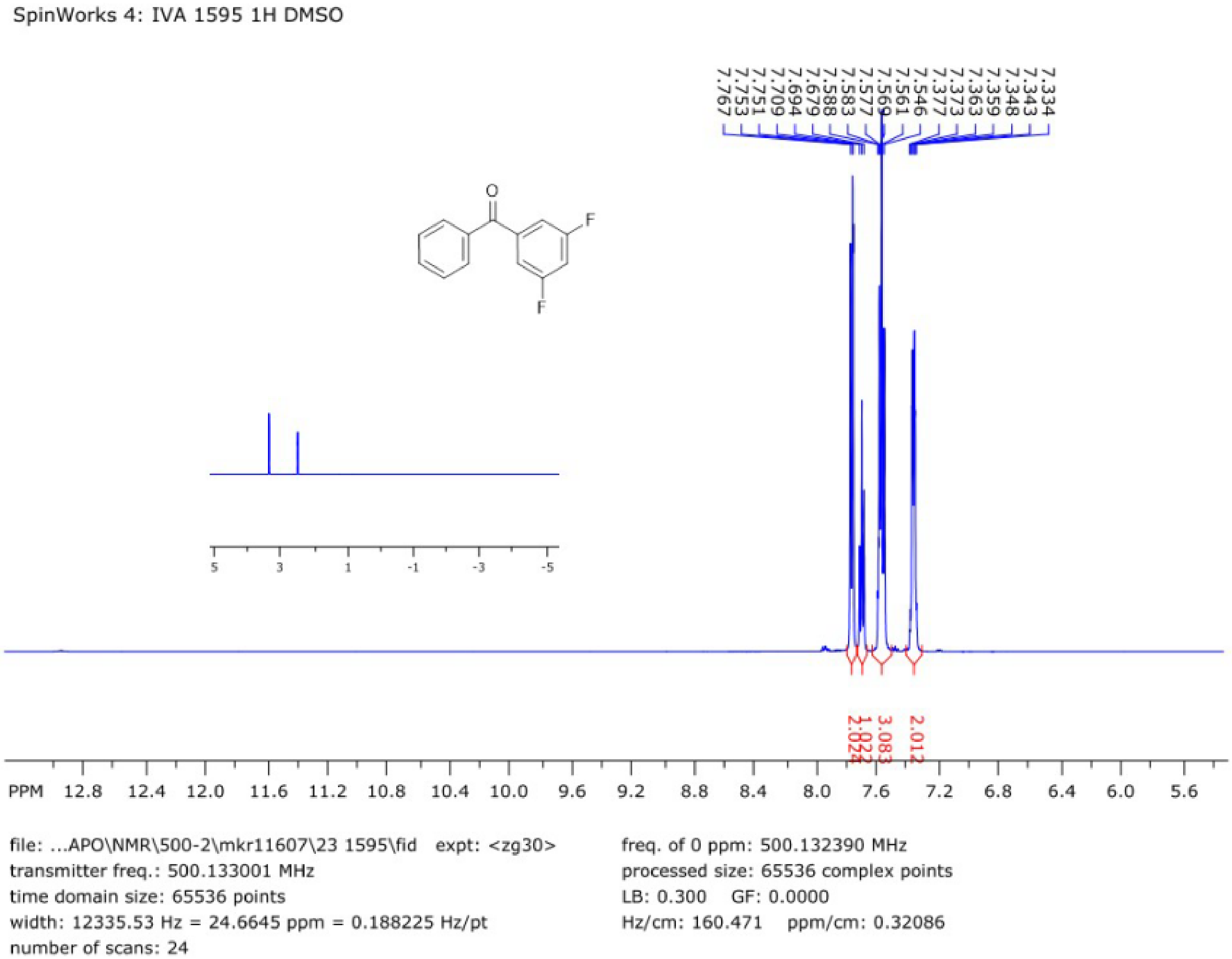

SpinWorks 4: IVA 1595 1H DMSO
7.767
7.753
7.751
7.709
7.694
7.679
7.588
7.583
7.577
7.569
7.561
7.546
7.377
7.373
7.363
7.359
7.348
7.343
7.334
O
F
F
5 3 1 -1 -3 -5
2.024
1.024
3.083
2.012
PPM 12.8 12.4 12.0 11.6 11.2 10.8 10.4 10.0 9.6 9.2 8.8 8.4 8.0 7.6 7.2 6.8 6.4 6.0 5.6
file: ...APO\NMR\500-2\mkr11607\23 1595\fid expt: <zg30>
transmitter freq.: 500.133001 MHz
time domain size: 65536 points
width: 12335.53 Hz = 24.6645 ppm = 0.188225 Hz/pt
number of scans: 24
freq. of 0 ppm: 500.132390 MHz
processed size: 65536 complex points
LB: 0.300 GF: 0.0000
Hz/cm: 160.471 ppm/cm: 0.32086

Compound 3d

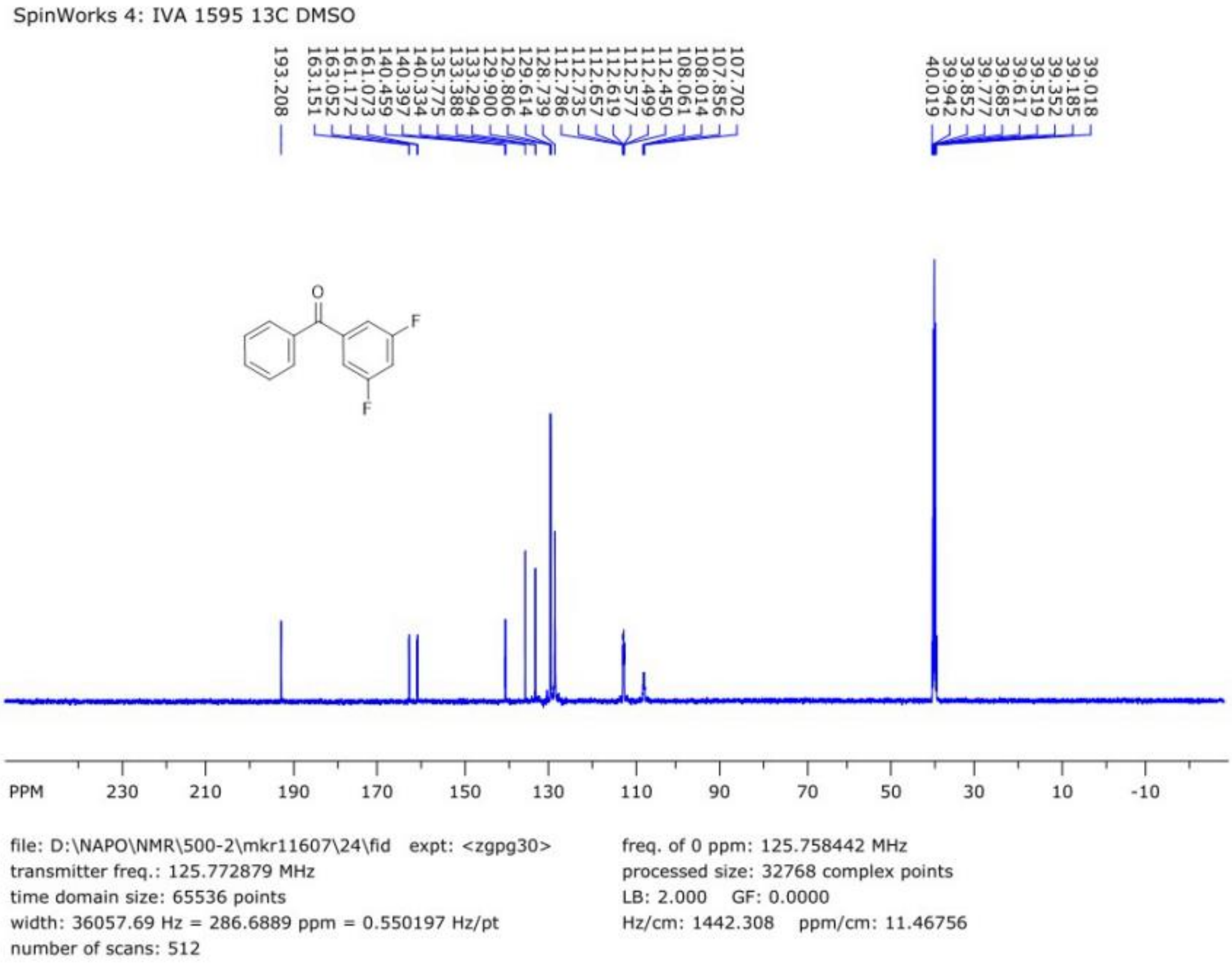
SpinWorks 4: IVA 1595 13C DMSO
193.208
163.151
163.052
161.172
161.073
140.459
140.397
140.334
135.775
133.388
133.294
129.900
129.806
129.614
128.739
112.786
112.735
112.657
112.619
112.577
112.499
112.450
108.061
108.014
107.856
107.702
40.019
39.942
39.852
39.777
39.685
39.617
39.519
39.352
39.185
39.018
O
F
F
PPM 230 210 190 170 150 130 110 90 70 50 30 10 -10
file: D:\NAPO\NMR\500-2\mkr11607\24\fid expt: <zgpg30>
transmitter freq.: 125.772879 MHz
time domain size: 65536 points
width: 36057.69 Hz = 286.6889 ppm = 0.550197 Hz/pt
number of scans: 512
freq. of 0 ppm: 125.758442 MHz
processed size: 32768 complex points
LB: 2.000 GF: 0.0000
Hz/cm: 1442.308 ppm/cm: 11.46756

Compound 3e

SpinWorks 4: SVS 215 1H CDCl3

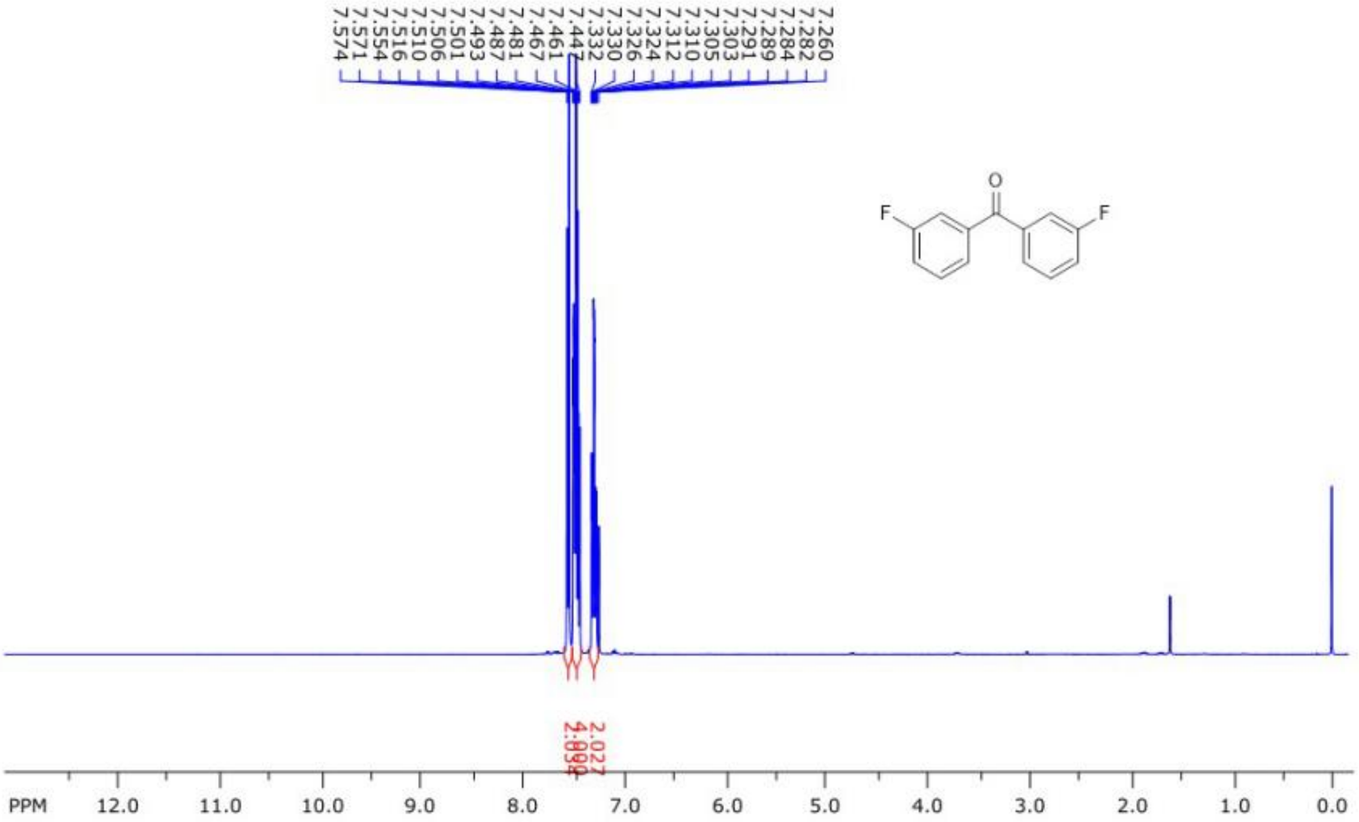

file: D:\NAPO\NMR\JELA\nmr\jn-215\1\fid expt: <zg30>
transmitter freq.: 400.132471 MHz
time domain size: 65536 points
width: 8196.72 Hz = 20.4850 ppm = 0.125072 Hz/pt
number of scans: 16

freq. of 0 ppm: 400.130009 MHz
processed size: 65536 complex points
LB: 0.300 GF: 0.0000
Hz/cm: 213.213 ppm/cm: 0.53286

Compound 3e

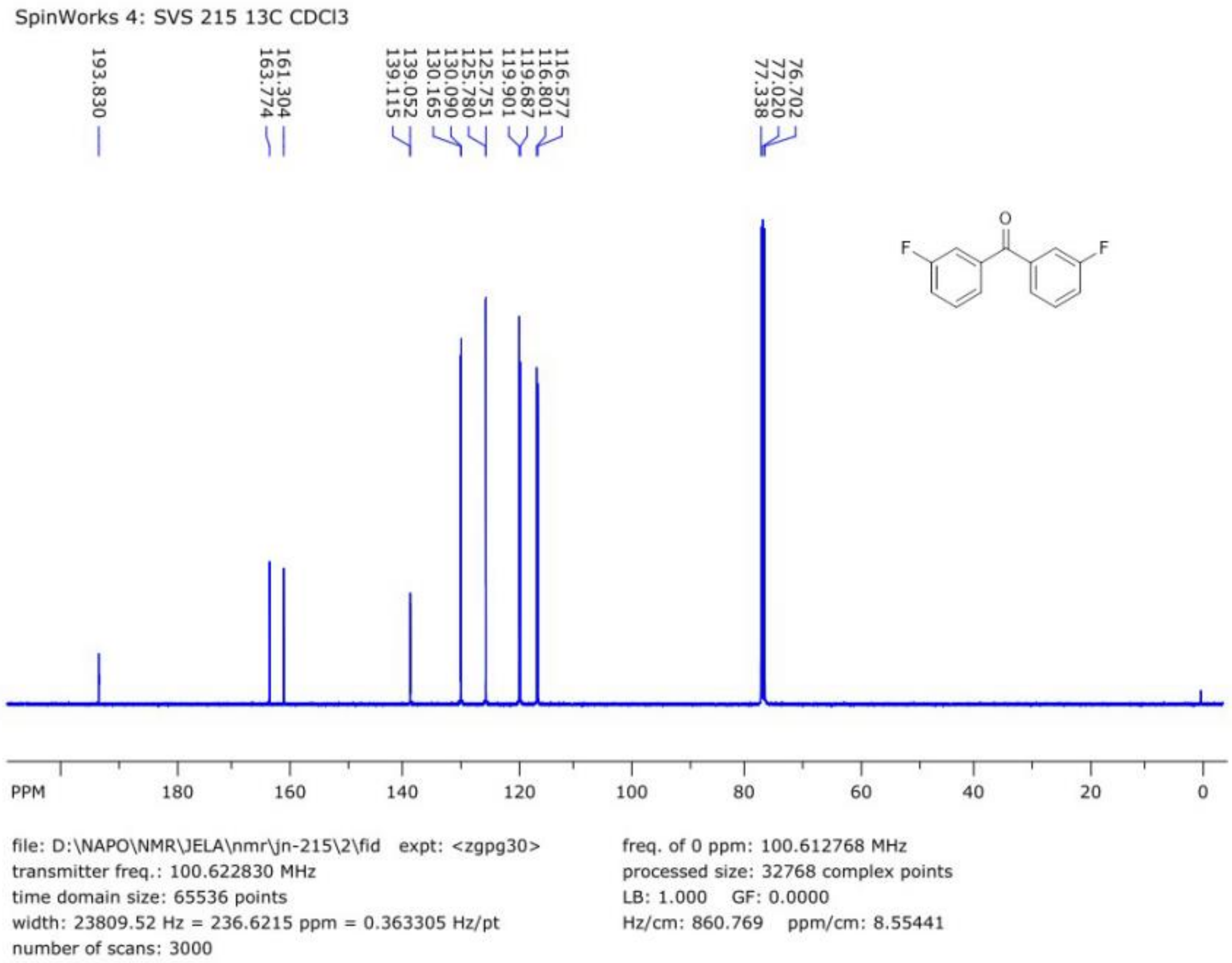
SpinWorks 4: SVS 215 13C CDCl3
193.830
163.774
161.304
139.115
139.052
130.165
130.090
125.780
125.751
119.901
119.687
116.801
116.577
77.338
77.020
76.702
O
F
F
PPM
180
160
140
120
100
80
60
40
20
0
file: D:\NAPO\NMR\JELA\nmr\jn-215\2\fid expt: <zgpg30>
transmitter freq.: 100.622830 MHz
time domain size: 65536 points
width: 23809.52 Hz = 236.6215 ppm = 0.363305 Hz/pt
number of scans: 3000
freq. of 0 ppm: 100.612768 MHz
processed size: 32768 complex points
LB: 1.000 GF: 0.0000
Hz/cm: 860.769 ppm/cm: 8.55441

Compound 3f

SpinWorks 4: SVS 306 1H CDCl3

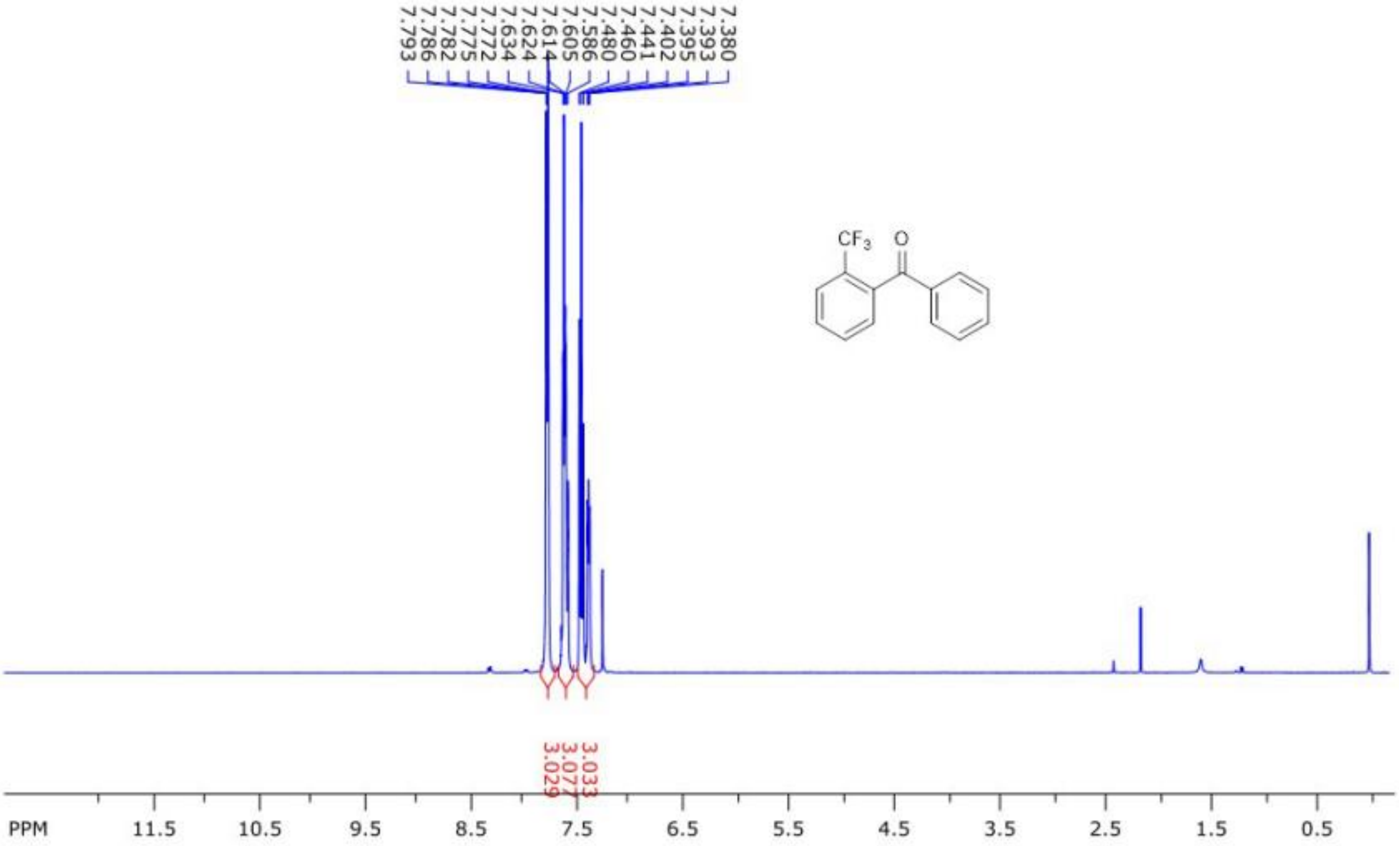

file: D:\NAPO\NMR\JELA\nmr\jn-306\1\fid expt: <zg30>
transmitter freq.: 400.132471 MHz
time domain size: 65536 points
width: 8196.72 Hz = 20.4850 ppm = 0.125072 Hz/pt
number of scans: 16

freq. of 0 ppm: 400.130009 MHz
processed size: 65536 complex points
LB: 0.300 GF: 0.0000
Hz/cm: 209.928 ppm/cm: 0.52465

Compound 3f

SpinWorks 4: SVS 306 13C CDCl3

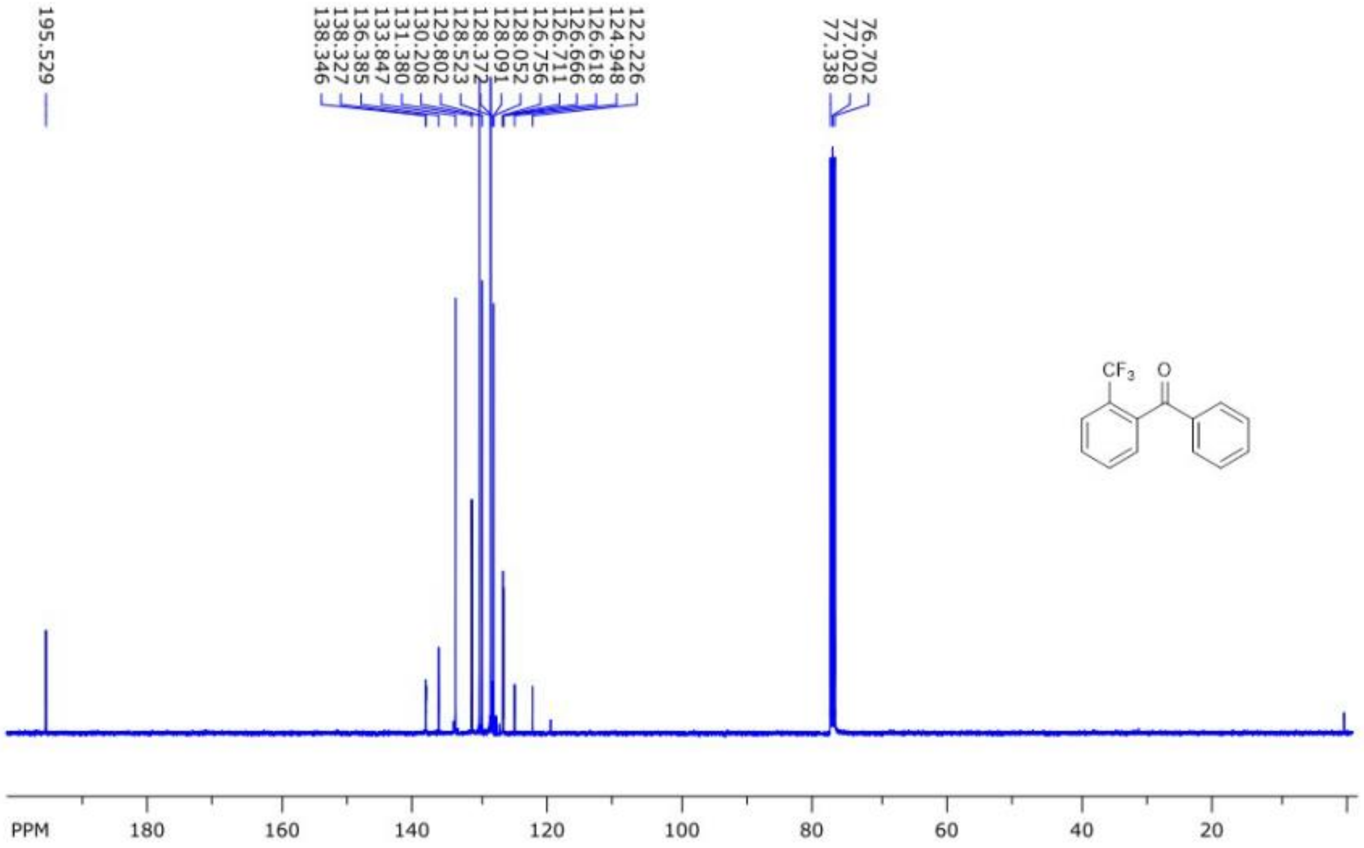

file: D:\NAPO\NMR\JELA\nmr\jn-306\2\fid expt: <zgpg30>
transmitter freq.: 100.622830 MHz
time domain size: 65536 points
width: 23809.52 Hz = 236.6215 ppm = 0.363305 Hz/pt
number of scans: 2000

freq. of 0 ppm: 100.612769 MHz
processed size: 32768 complex points
LB: 1.000 GF: 0.0000
Hz/cm: 815.918 ppm/cm: 8.10867

Compound 3g

SpinWorks 4: IVA 1880 1H CDCl3

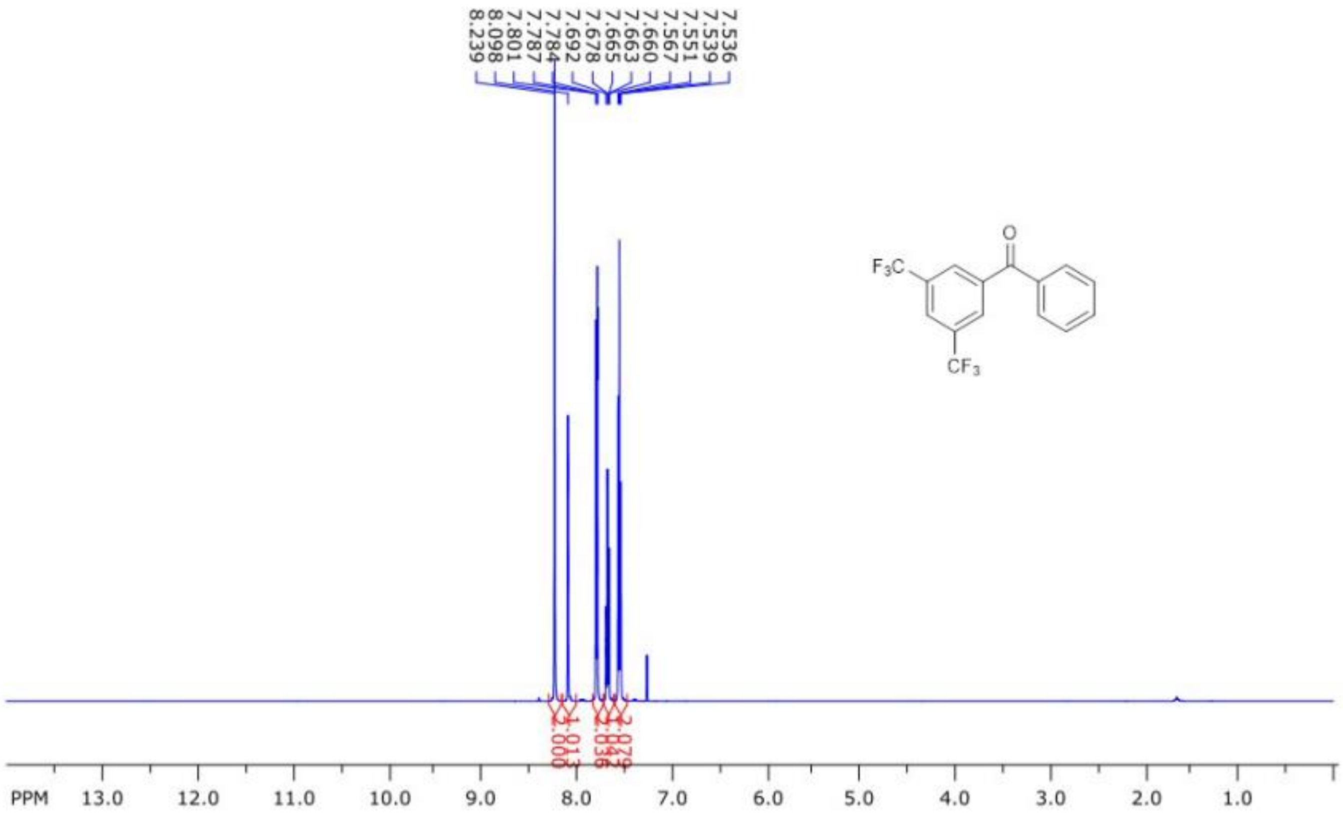

file: ...ketones\Fids part 1\IVA 1880\5\fid expt: <zg30>
transmitter freq.: 500.133001 MHz
time domain size: 65536 points
width: 12335.53 Hz = 24.6645 ppm = 0.188225 Hz/pt
number of scans: 24

freq. of 0 ppm: 500.127638 MHz
processed size: 65536 complex points
LB: 0.300 GF: 0.0000
Hz/cm: 281.814 ppm/cm: 0.56348

Compound 3g

SpinWorks 4: IVA 1880 13C CDCL3

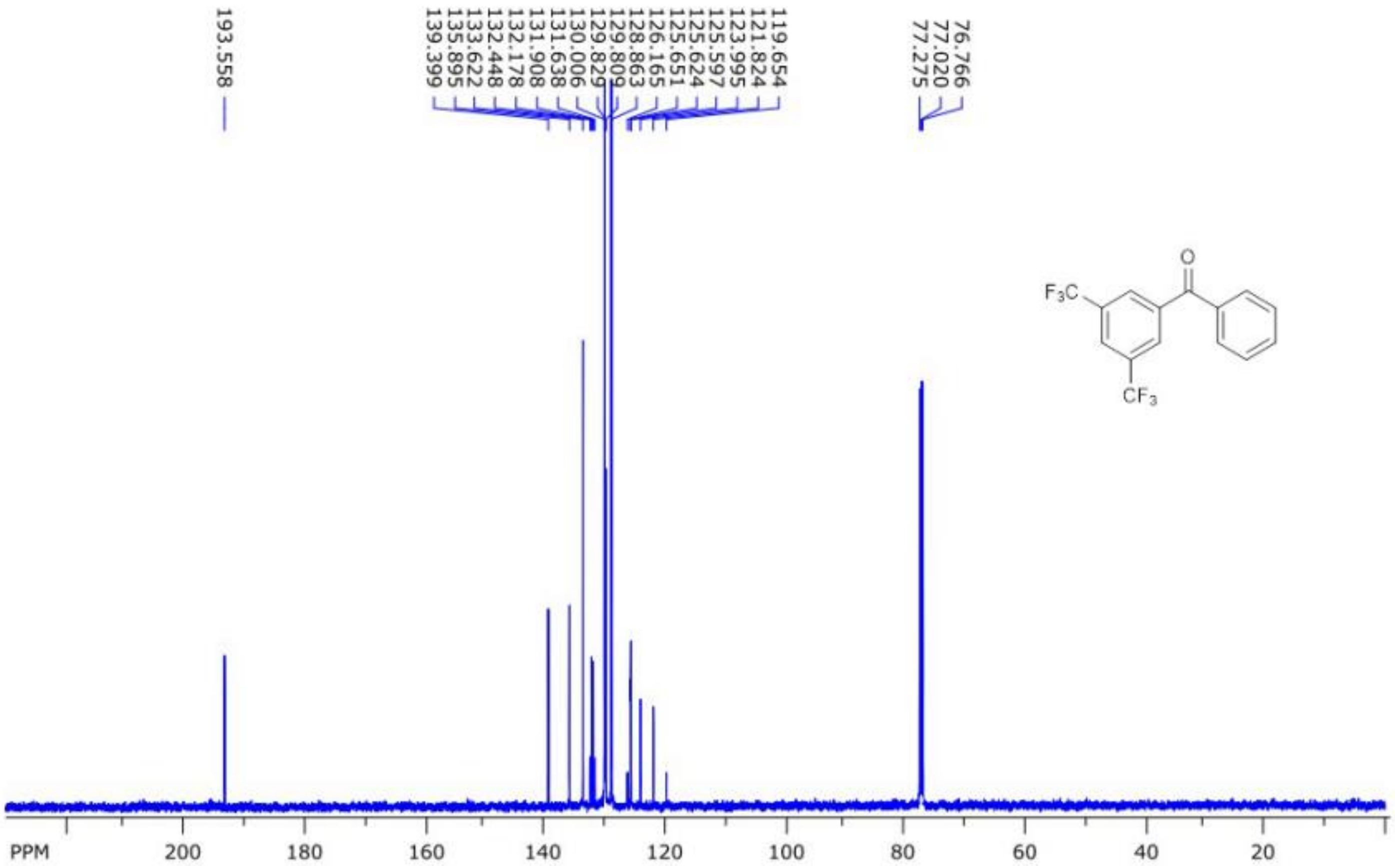

file: ...ketones\Fids part 1\IVA 1880\6\fid expt: <zgpg30>
transmitter freq.: 125.772879 MHz
time domain size: 65536 points
width: 36057.69 Hz = 286.6889 ppm = 0.550197 Hz/pt
number of scans: 512

freq. of 0 ppm: 125.757190 MHz
processed size: 32768 complex points
LB: 2.000 GF: 0.0000
Hz/cm: 1160.494 ppm/cm: 9.22690

Compound 3h

SpinWorks 4: SVS 218 1H CDCl3

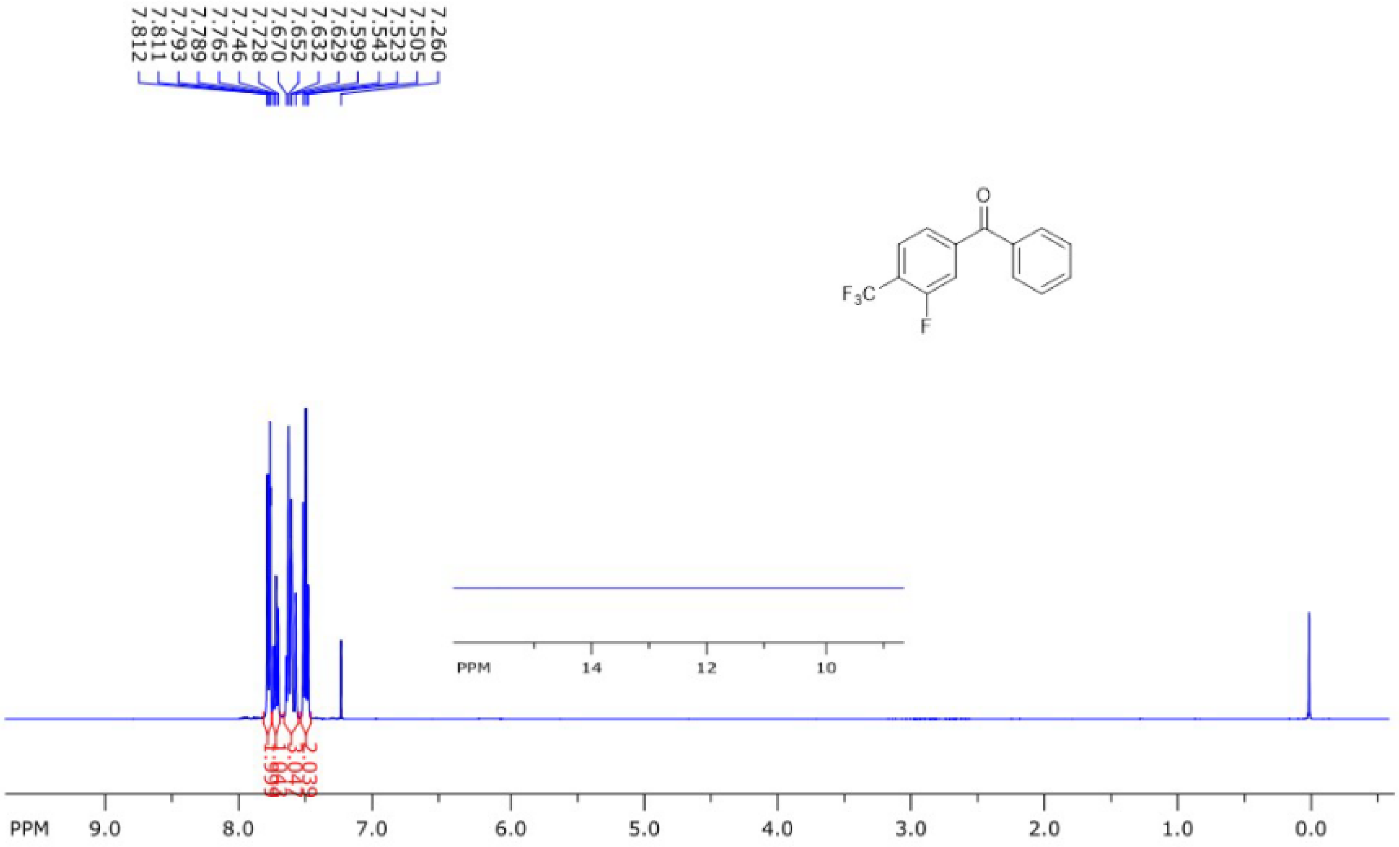

file: D:\NAPO\NMR\JELA\nmr\jn-218\1\fid expt: <zg30>
transmitter freq.: 400.132471 MHz
time domain size: 65536 points
width: 8196.72 Hz = 20.4850 ppm = 0.125072 Hz/pt
number of scans: 16

freq. of 0 ppm: 400.130009 MHz
processed size: 65536 complex points
LB: 0.300 GF: 0.0000
Hz/cm: 166.111 ppm/cm: 0.41514

Compound 3h

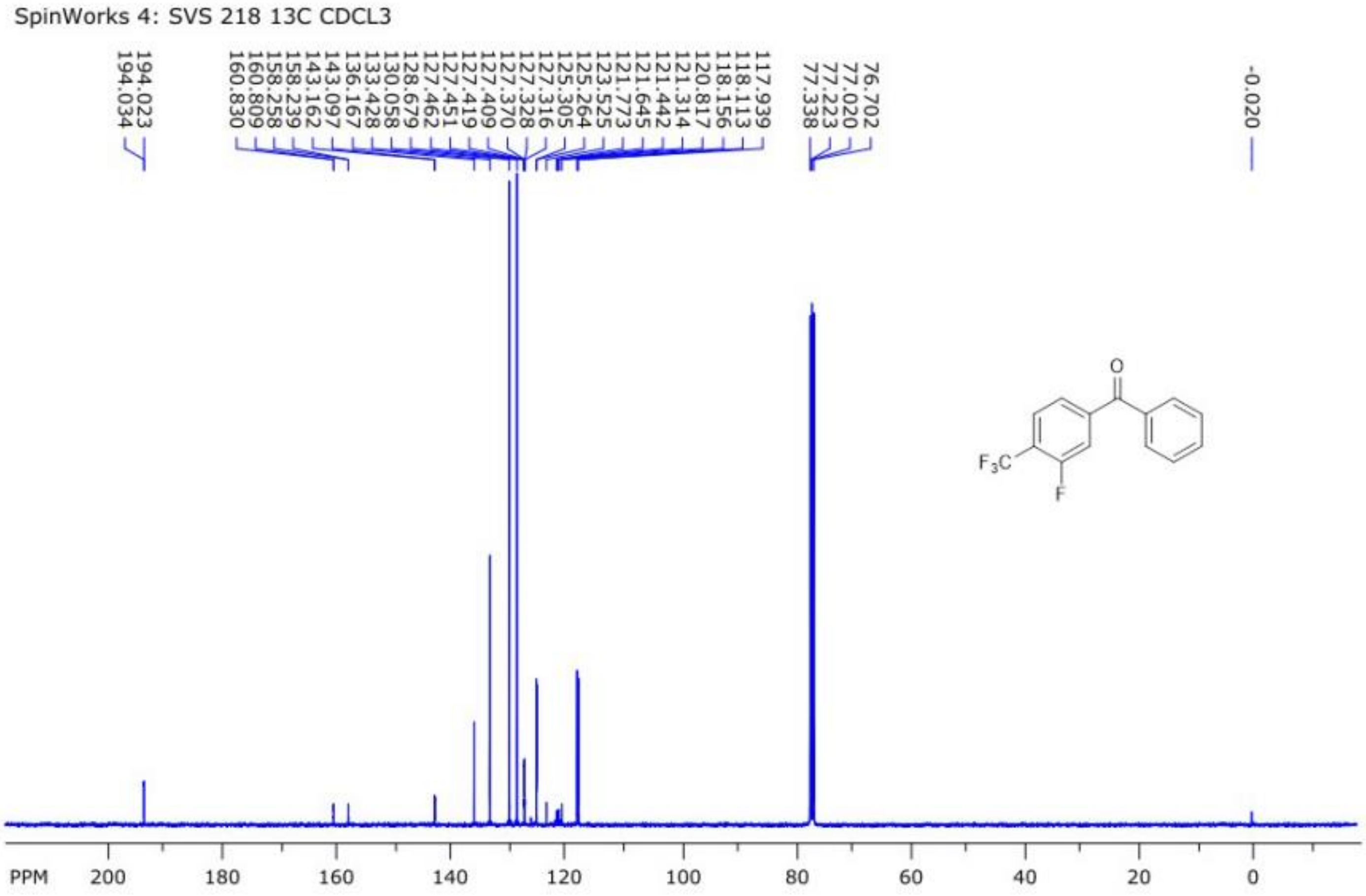

file: D:\NAPO\NMR\JELA\nmr\jn-218\2\fid expt: <zgpg30>
transmitter freq.: 100.622830 MHz
time domain size: 65536 points
width: 23809.52 Hz = 236.6215 ppm = 0.363305 Hz/pt
number of scans: 3500

freq. of 0 ppm: 100.612767 MHz
processed size: 32768 complex points
LB: 1.000 GF: 0.0000
Hz/cm: 952.381 ppm/cm: 9.46486

Compound 3i

SpinWorks 4: IVA 1597 1H DMSO

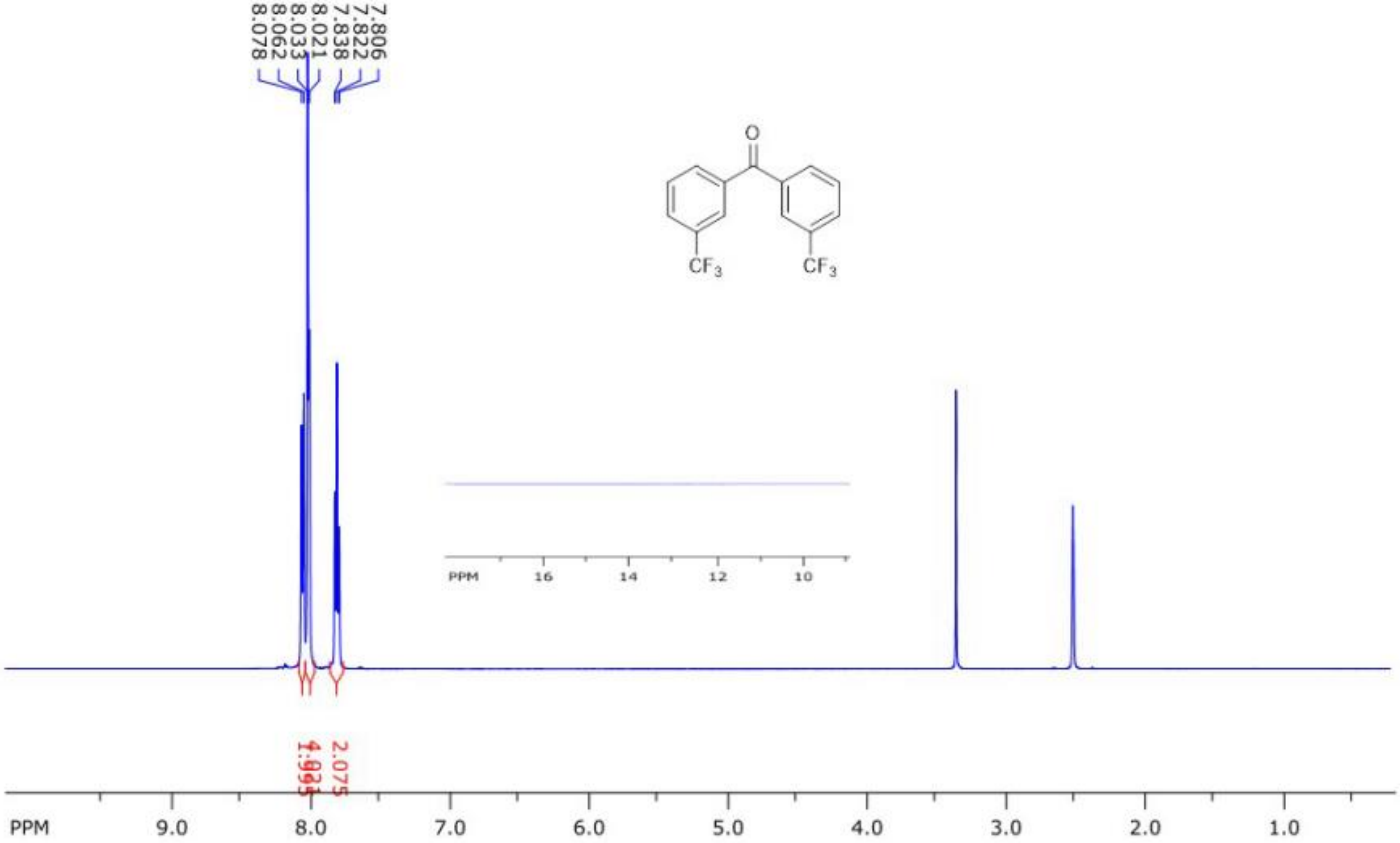

file: ...APO\NMR\500-2\mkr11207\23 1597\fid expt: <zg30>
transmitter freq.: 500.133001 MHz
time domain size: 65536 points
width: 12335.53 Hz = 24.6645 ppm = 0.188225 Hz/pt
number of scans: 24

freq. of 0 ppm: 500.130005 MHz
processed size: 65536 complex points
LB: 0.300 GF: 0.0000
Hz/cm: 200.316 ppm/cm: 0.40053

Compound 3i

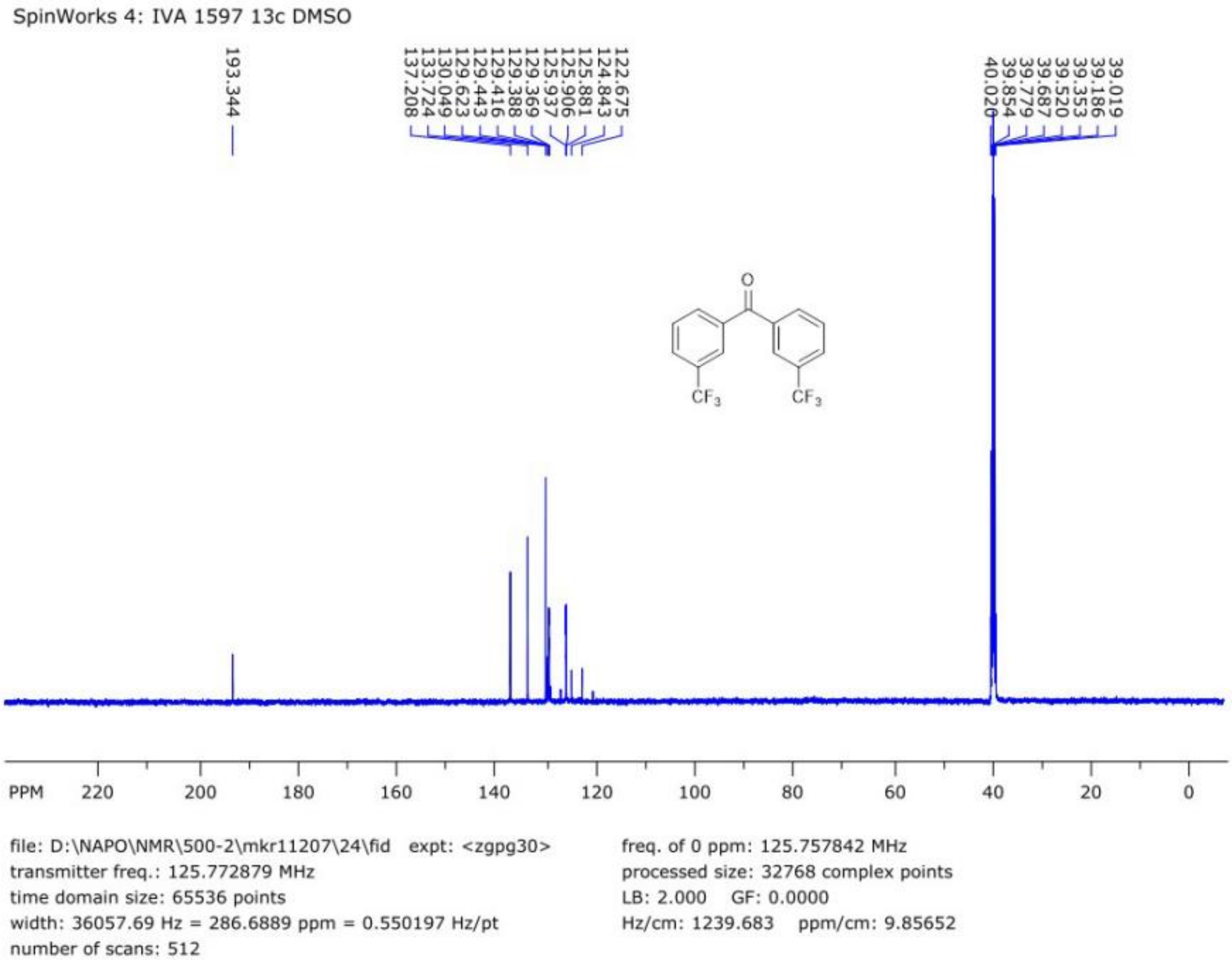
SpinWorks 4: IVA 1597 13c DMSO
193.344
137.208
133.724
130.049
129.623
129.443
129.416
129.388
129.369
125.937
125.906
125.881
124.843
122.675
40.020
39.854
39.779
39.687
39.520
39.353
39.186
39.019
O
CF3
CF3
PPM 220 200 180 160 140 120 100 80 60 40 20 0
file: D:\NAPO\NMR\500-2\mkr11207\24\fid expt: <zgpg30>
transmitter freq.: 125.772879 MHz
time domain size: 65536 points
width: 36057.69 Hz = 286.6889 ppm = 0.550197 Hz/pt
number of scans: 512
freq. of 0 ppm: 125.757842 MHz
processed size: 32768 complex points
LB: 2.000 GF: 0.0000
Hz/cm: 1239.683 ppm/cm: 9.85652

Compound 3j

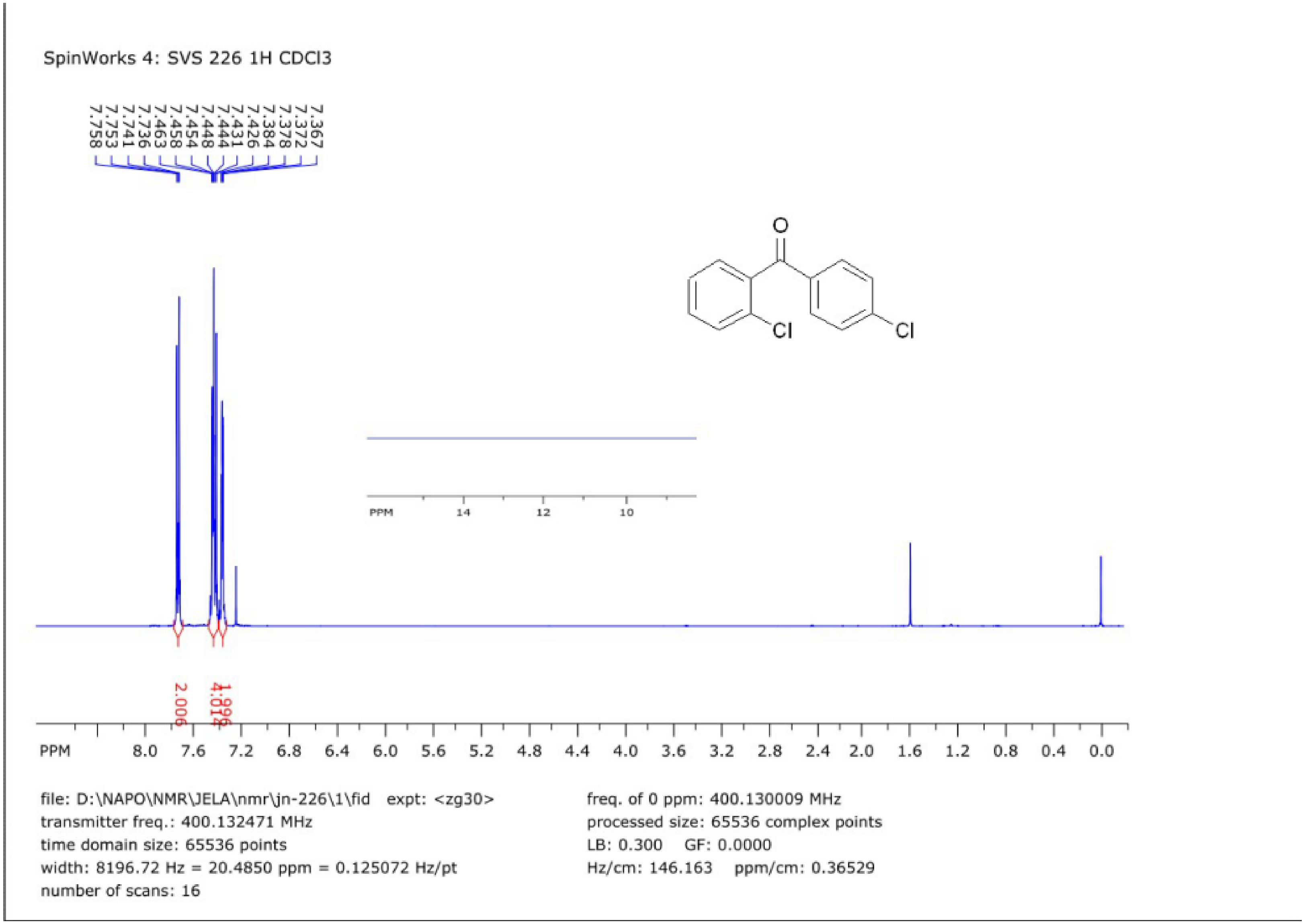

SpinWorks 4: SVS 226 1H CDCl3
7.758
7.753
7.741
7.736
7.463
7.458
7.454
7.448
7.444
7.431
7.426
7.384
7.378
7.372
7.367
O
Cl
Cl
PPM
14
12
10
2.006
4.012
1.996
PPM
8.0
7.6
7.2
6.8
6.4
6.0
5.6
5.2
4.8
4.4
4.0
3.6
3.2
2.8
2.4
2.0
1.6
1.2
0.8
0.4
0.0
file: D:\NAPO\NMR\JELA\nmr\jn-226\1\fid expt: <zg30>
transmitter freq.: 400.132471 MHz
time domain size: 65536 points
width: 8196.72 Hz = 20.4850 ppm = 0.125072 Hz/pt
number of scans: 16
freq. of 0 ppm: 400.130009 MHz
processed size: 65536 complex points
LB: 0.300 GF: 0.0000
Hz/cm: 146.163 ppm/cm: 0.36529

Compound 3j

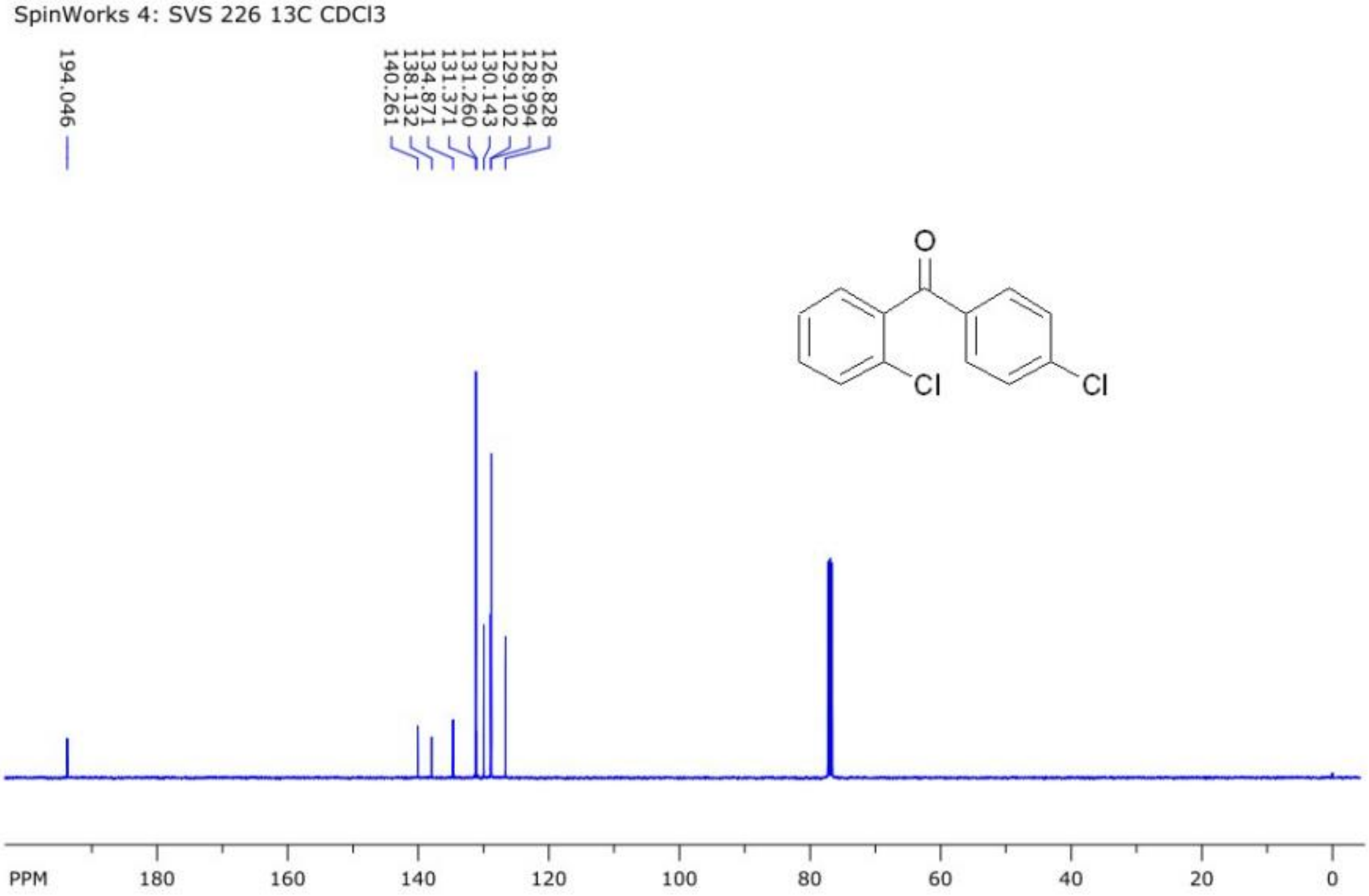

file: D:\NAPO\NMR\JELA\nmr\jn-226\2\fid expt: <zgpg30>
transmitter freq.: 100.622830 MHz
time domain size: 65536 points
width: 23809.52 Hz = 236.6215 ppm = 0.363305 Hz/pt
number of scans: 2000

freq. of 0 ppm: 100.612770 MHz
processed size: 32768 complex points
LB: 1.000 GF: 0.0000
Hz/cm: 836.494 ppm/cm: 8.31316

Compound 3k

SpinWorks 4: IVAB 3684 1H CDCl3

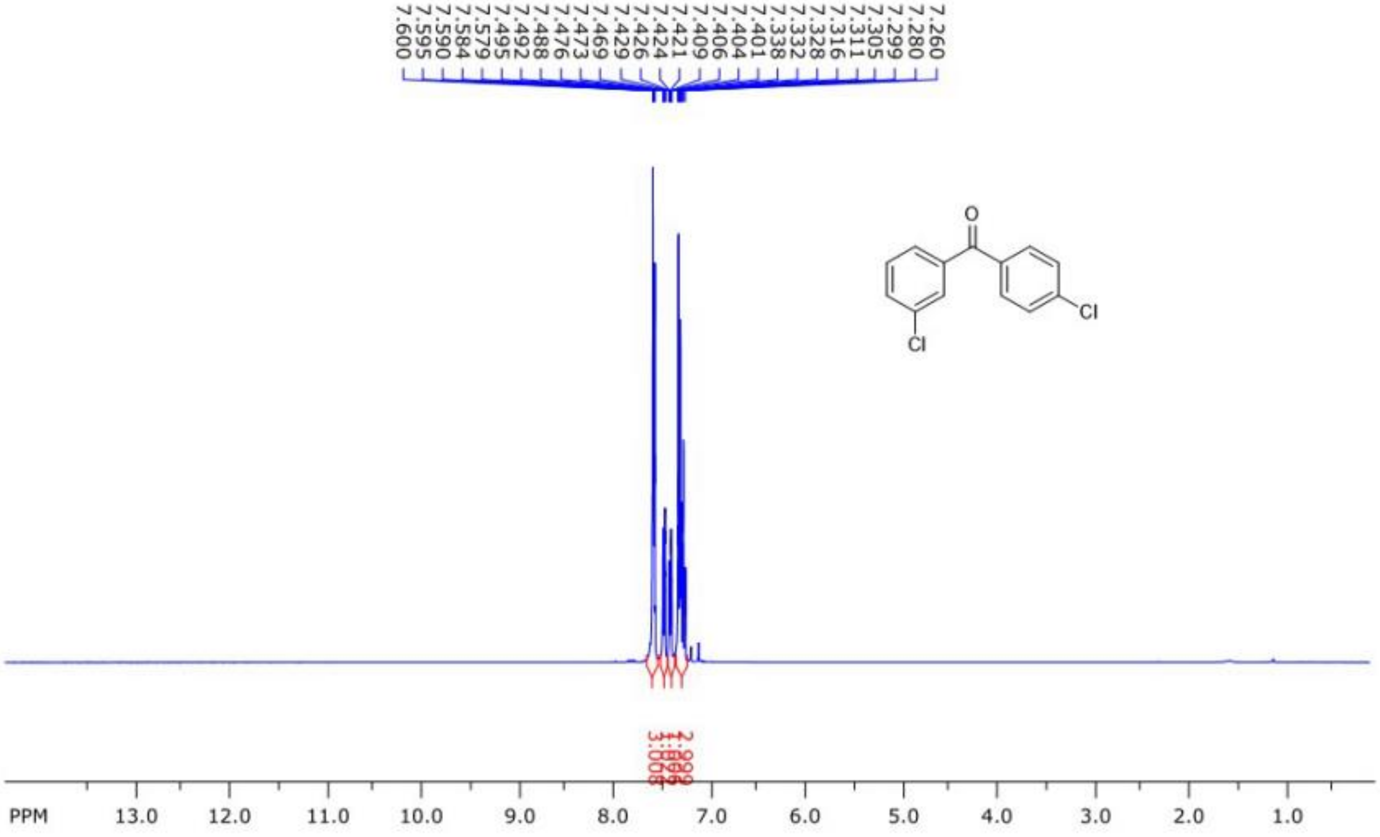

file: ...tones\Fids part 1\Ivab-3684\19\fid expt: <zg30>
transmitter freq.: 400.133001 MHz
time domain size: 65536 points
width: 6393.86 Hz = 15.9793 ppm = 0.097563 Hz/pt
number of scans: 64

freq. of 0 ppm: 400.130066 MHz
processed size: 65536 complex points
LB: 0.300 GF: 0.0000
Hz/cm: 228.590 ppm/cm: 0.57129

Compound 3k

SpinWorks 4: IVAB 3684 13C DMSO

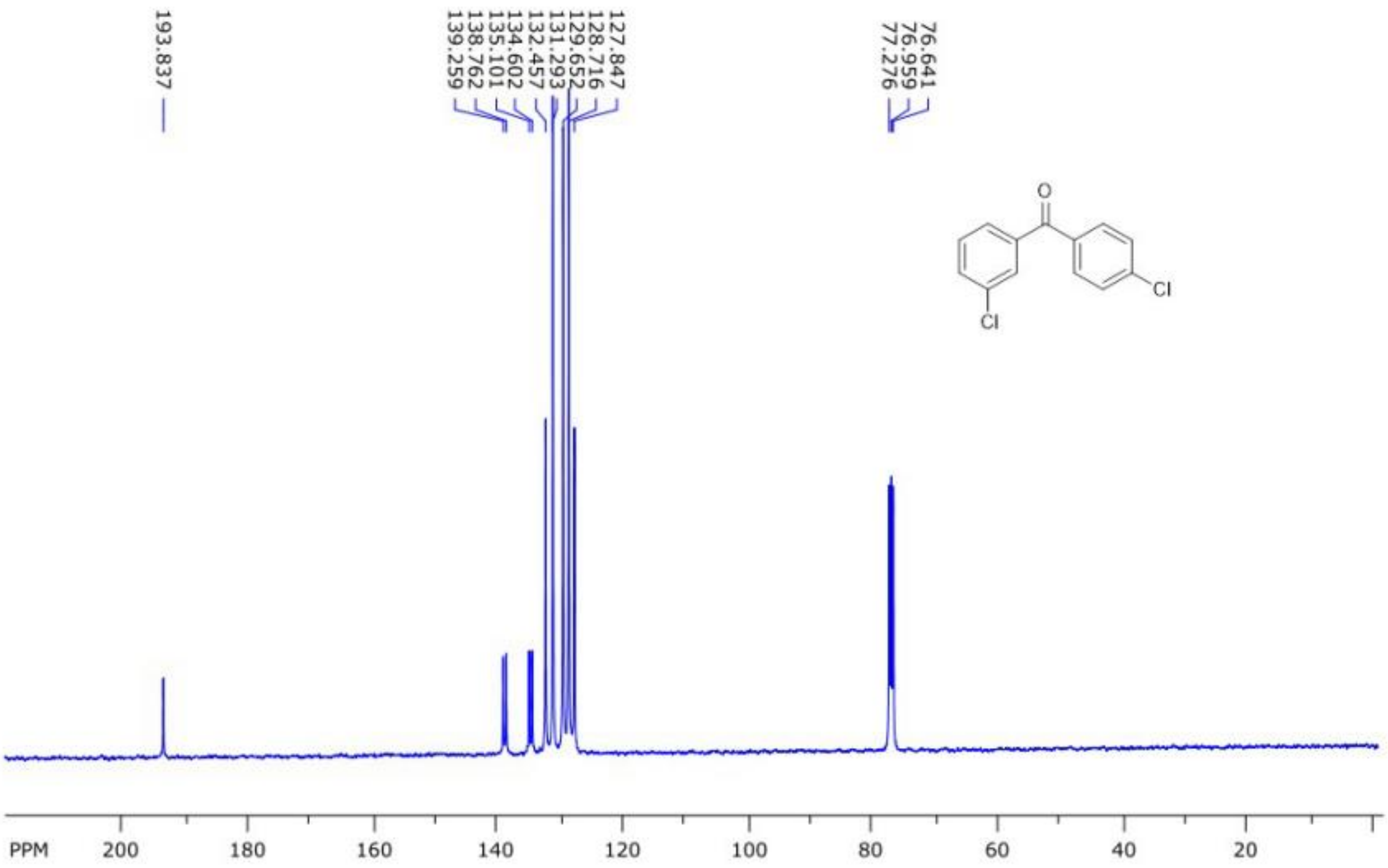

file: ...tones\Fids part 1\Ivab-3684\20\fid expt: <zgpg30>
transmitter freq.: 100.623836 MHz
time domain size: 65536 points
width: 24038.46 Hz = 238.8943 ppm = 0.366798 Hz/pt
number of scans: 1335

freq. of 0 ppm: 100.612780 MHz
processed size: 32768 complex points
LB: 10.000 GF: 0.0000
Hz/cm: 887.352 ppm/cm: 8.81850

Compound 3l

SpinWorks 4: IVAB 3680 1H CDCl3

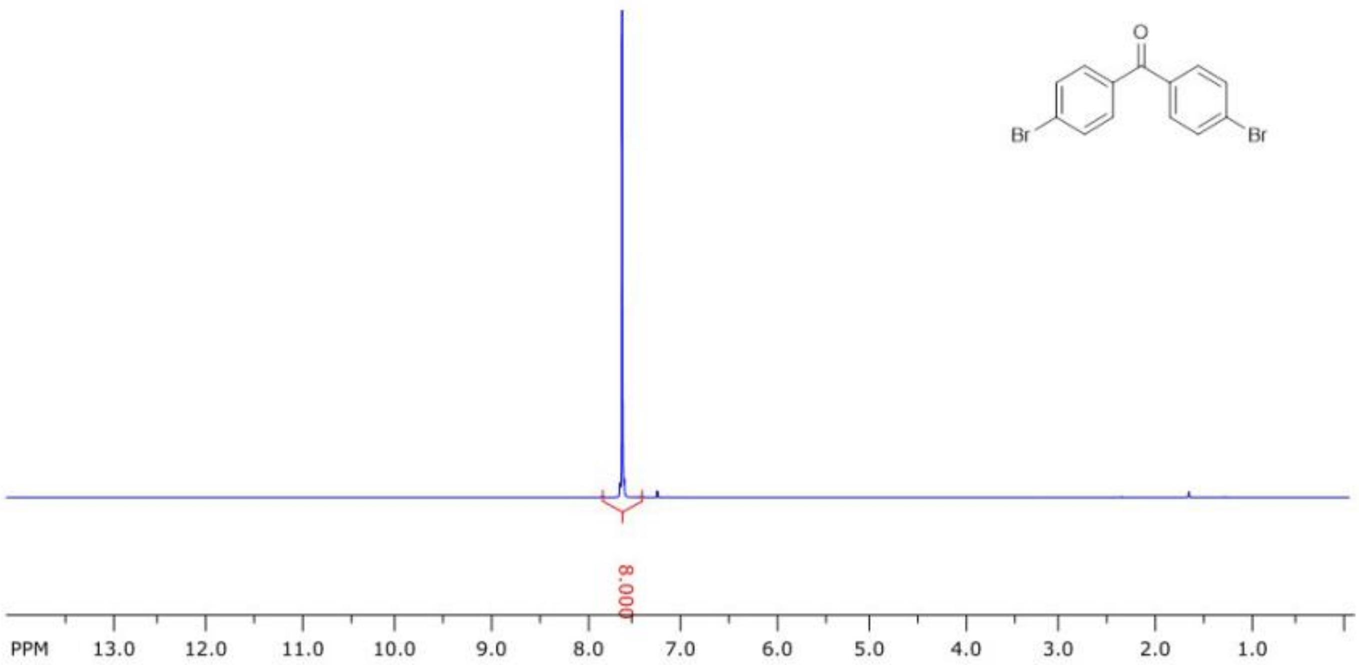

file: ...tones\Fids part 1\Ivab-3680\15\fid expt: <zg30>
transmitter freq.: 400.133001 MHz
time domain size: 65536 points
width: 6393.86 Hz = 15.9793 ppm = 0.097563 Hz/pt
number of scans: 64

freq. of 0 ppm: 400.130013 MHz
processed size: 65536 complex points
LB: 0.300 GF: 0.0000
Hz/cm: 227.565 ppm/cm: 0.56872

Compound 3l

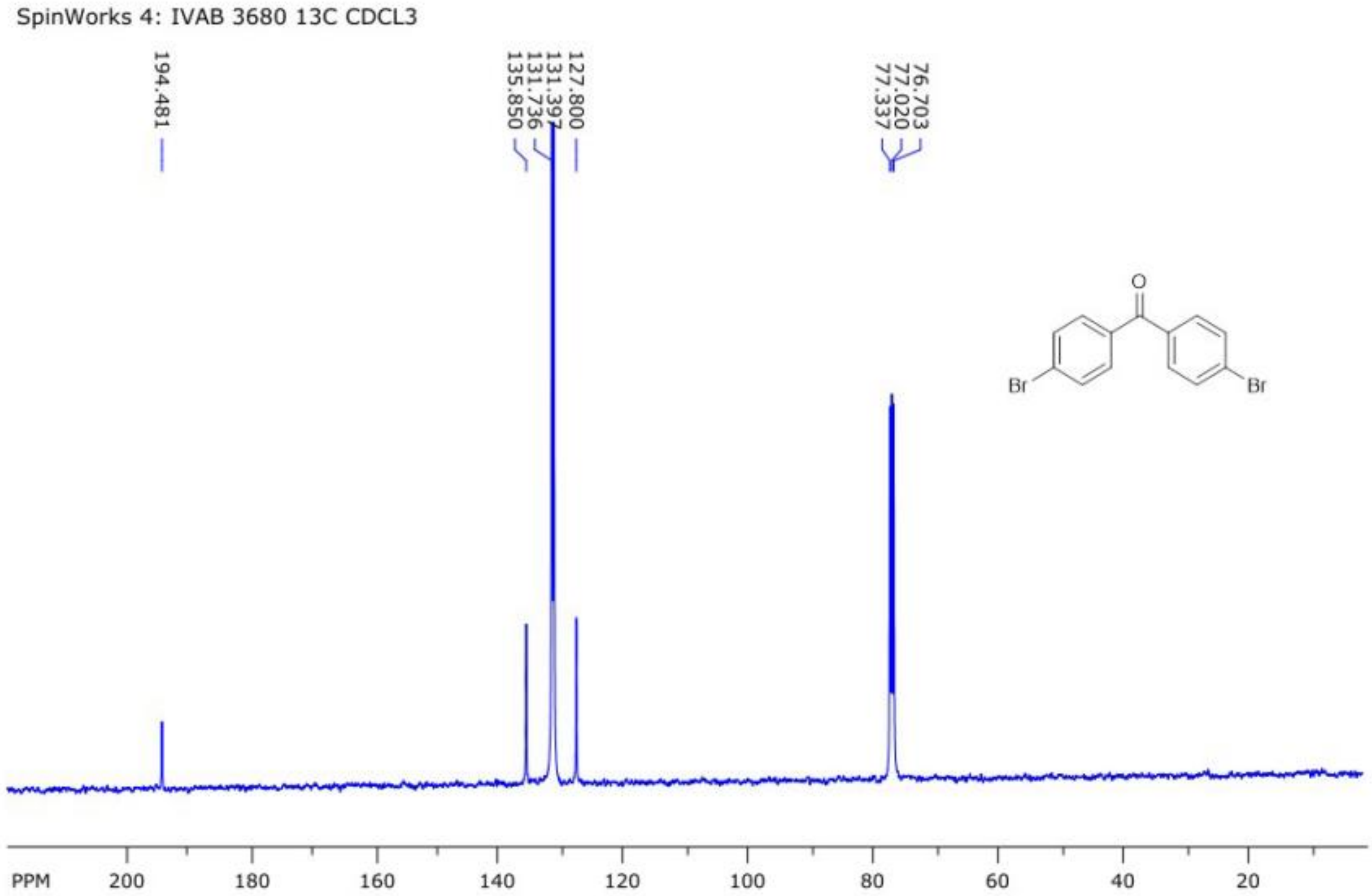

file: ...tones\Fids part 1\Ivab-3680\16\fid expt: <zgpg30>
transmitter freq.: 100.623836 MHz
time domain size: 65536 points
width: 24038.46 Hz = 238.8943 ppm = 0.366798 Hz/pt
number of scans: 768

freq. of 0 ppm: 100.612771 MHz
processed size: 32768 complex points
LB: 10.000 GF: 0.0000
Hz/cm: 877.717 ppm/cm: 8.72275

Compound 3m

SpinWorks 4: IVAB 3677 1H CDCL3

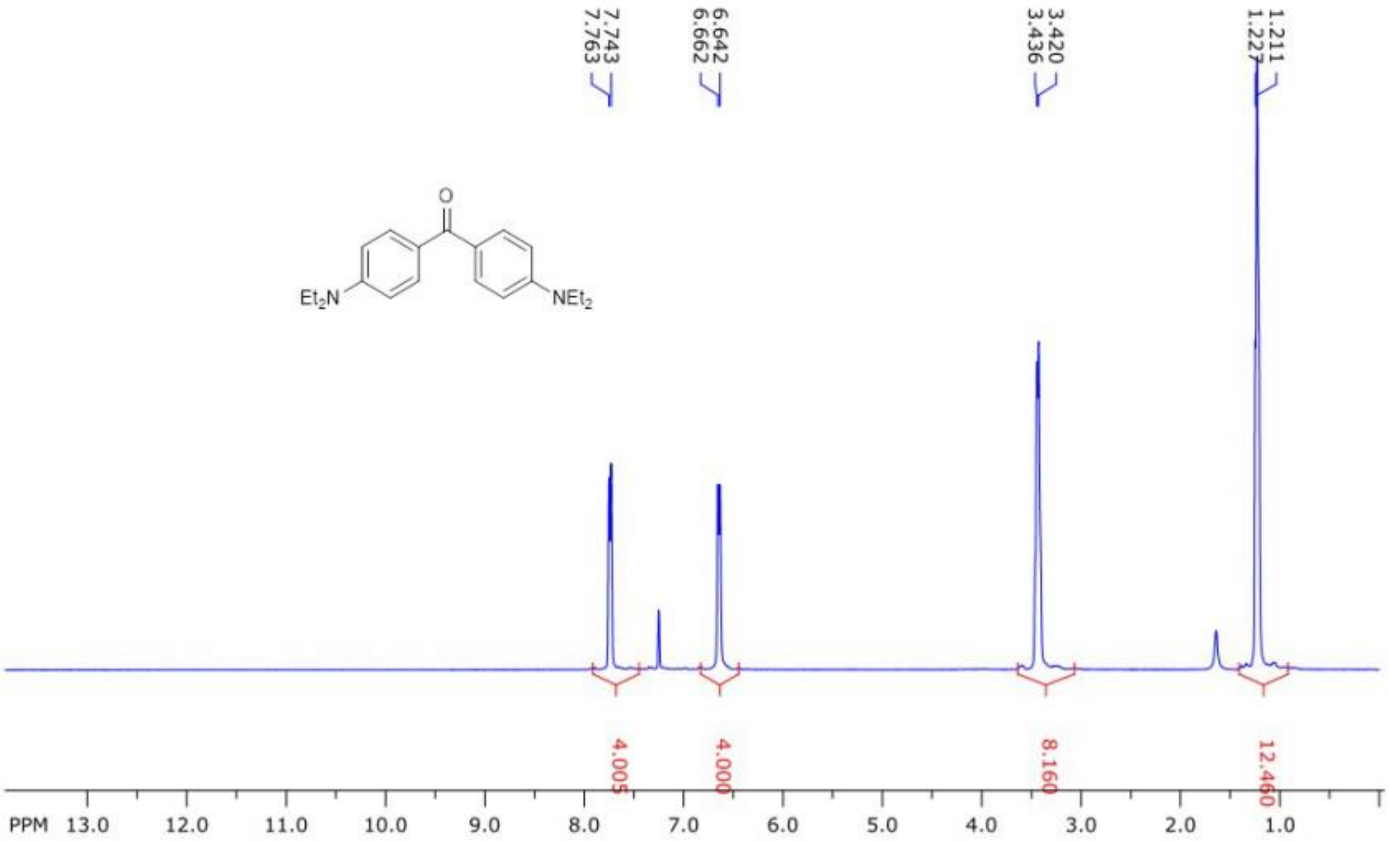

file: ...tones\Fids part 1\Ivab-3677\13\fid expt: <zg30>
transmitter freq.: 400.133001 MHz
time domain size: 65536 points
width: 6393.86 Hz = 15.9793 ppm = 0.097563 Hz/pt
number of scans: 64

freq. of 0 ppm: 400.130012 MHz
processed size: 65536 complex points
LB: 0.300 GF: 0.0000
Hz/cm: 222.440 ppm/cm: 0.55591

Compound 3m

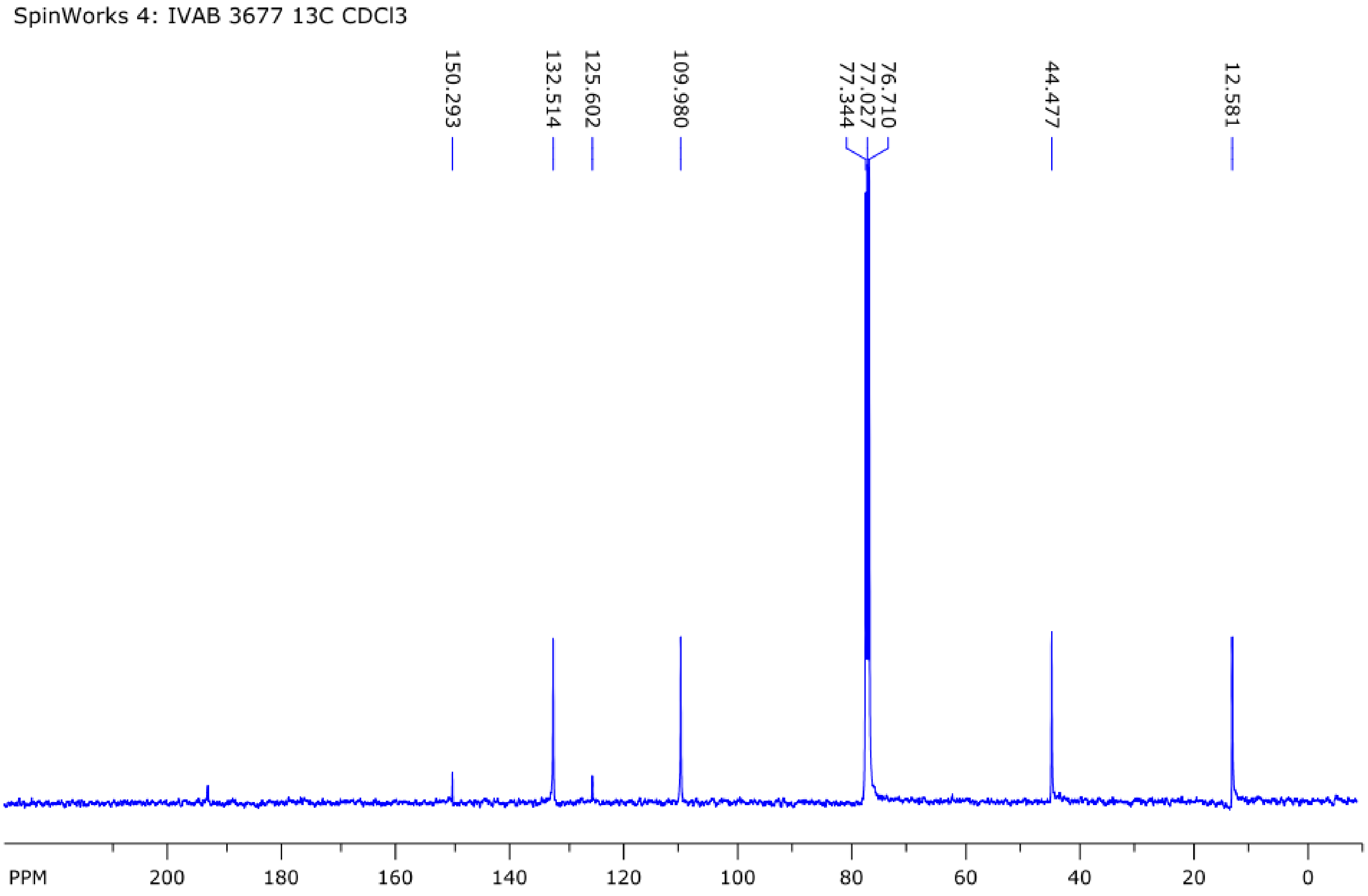

file: ...tones\Fids part 1\Ivab-3677\14\fid expt: <zgpg30>
transmitter freq.: 100.623836 MHz
time domain size: 65536 points
width: 24038.46 Hz = 238.8943 ppm = 0.366798 Hz/pt
number of scans: 1636

freq. of 0 ppm: 100.612769 MHz
processed size: 32768 complex points
LB: 10.000 GF: 0.0000
Hz/cm: 961.538 ppm/cm: 9.55577

Compound 3n

SpinWorks 4: IVAB 3647 1H CDCl3

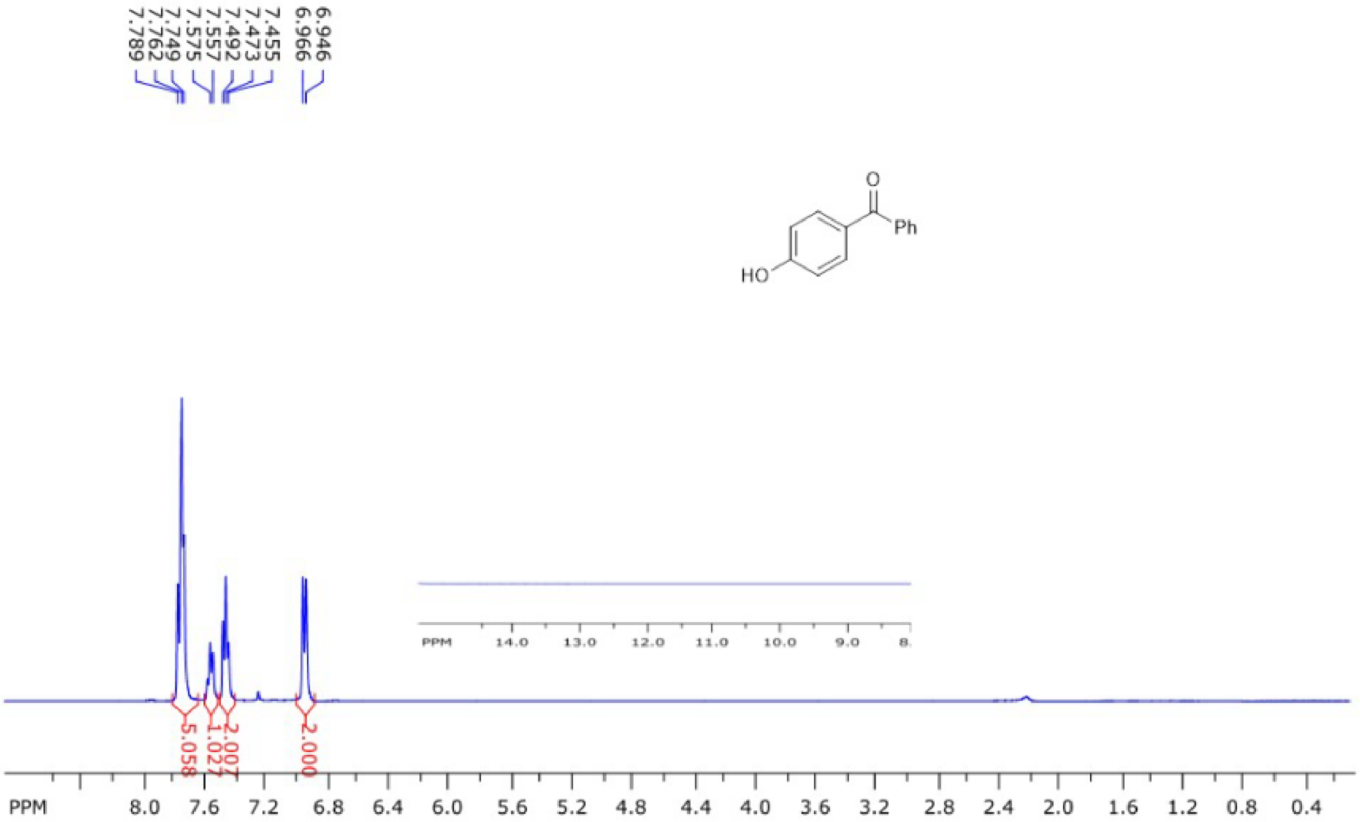

file: ...chael Slovakia\NMR\Ivab-3647\5\fid expt: <zg30>
transmitter freq.: 400.133001 MHz
time domain size: 65536 points
width: 6393.86 Hz = 15.9793 ppm = 0.097563 Hz/pt
number of scans: 64

freq. of 0 ppm: 400.130009 MHz
processed size: 65536 complex points
LB: 0.300 GF: 0.0000
Hz/cm: 141.457 ppm/cm: 0.35353

Compound 3n

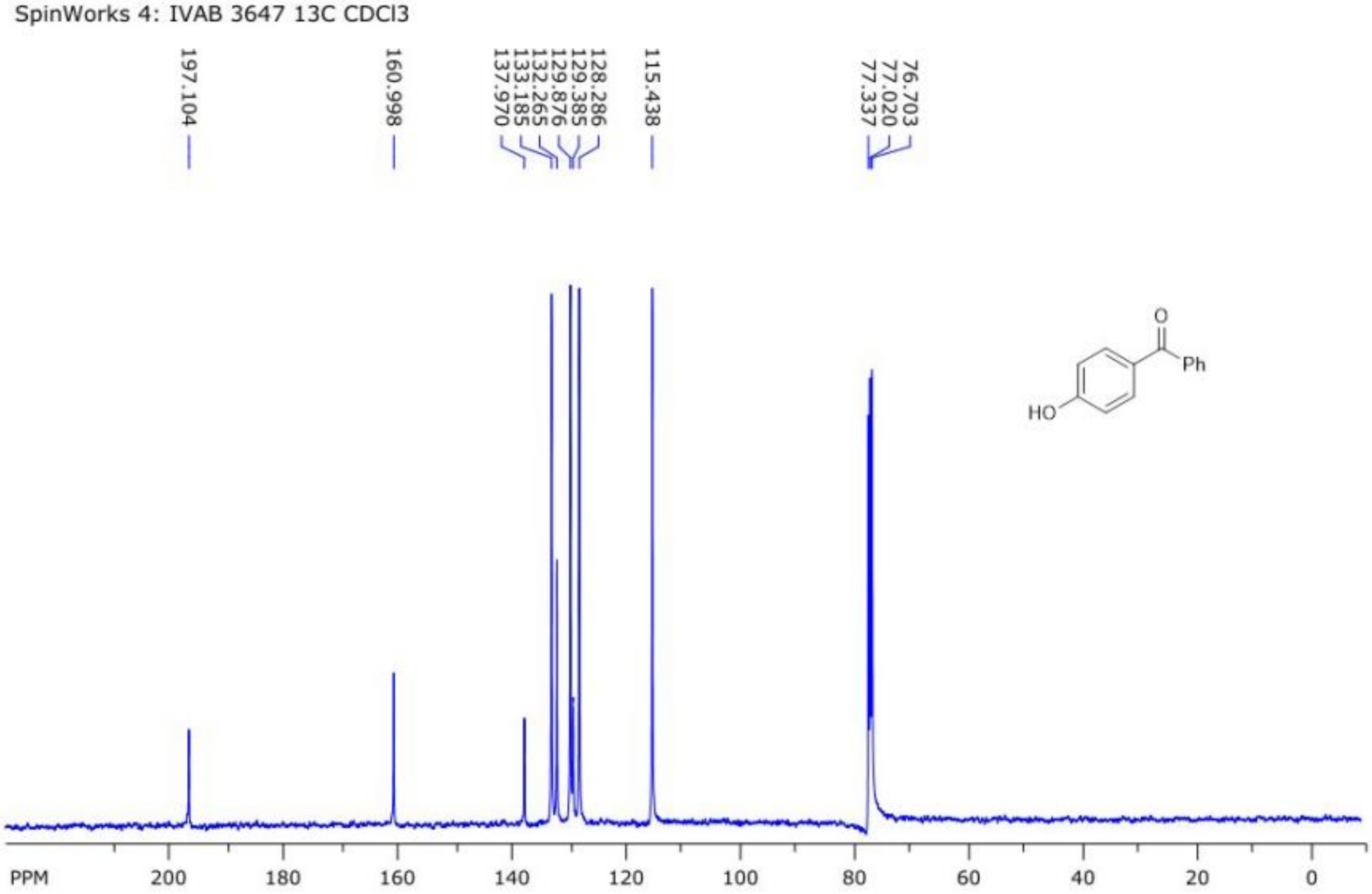

file: ...chael Slovakia\NMR\Ivab-3647\6\fid expt: <zgpg30>
transmitter freq.: 100.623836 MHz
time domain size: 65536 points
width: 24038.46 Hz = 238.8943 ppm = 0.366798 Hz/pt
[illegible]

freq. of 0 ppm: 100.612771 MHz
processed size: 32768 complex points
LB: 10.000 GF: 0.0000
Hz/cm: 961.538 ppm/cm: 9.55577

Compound 3o

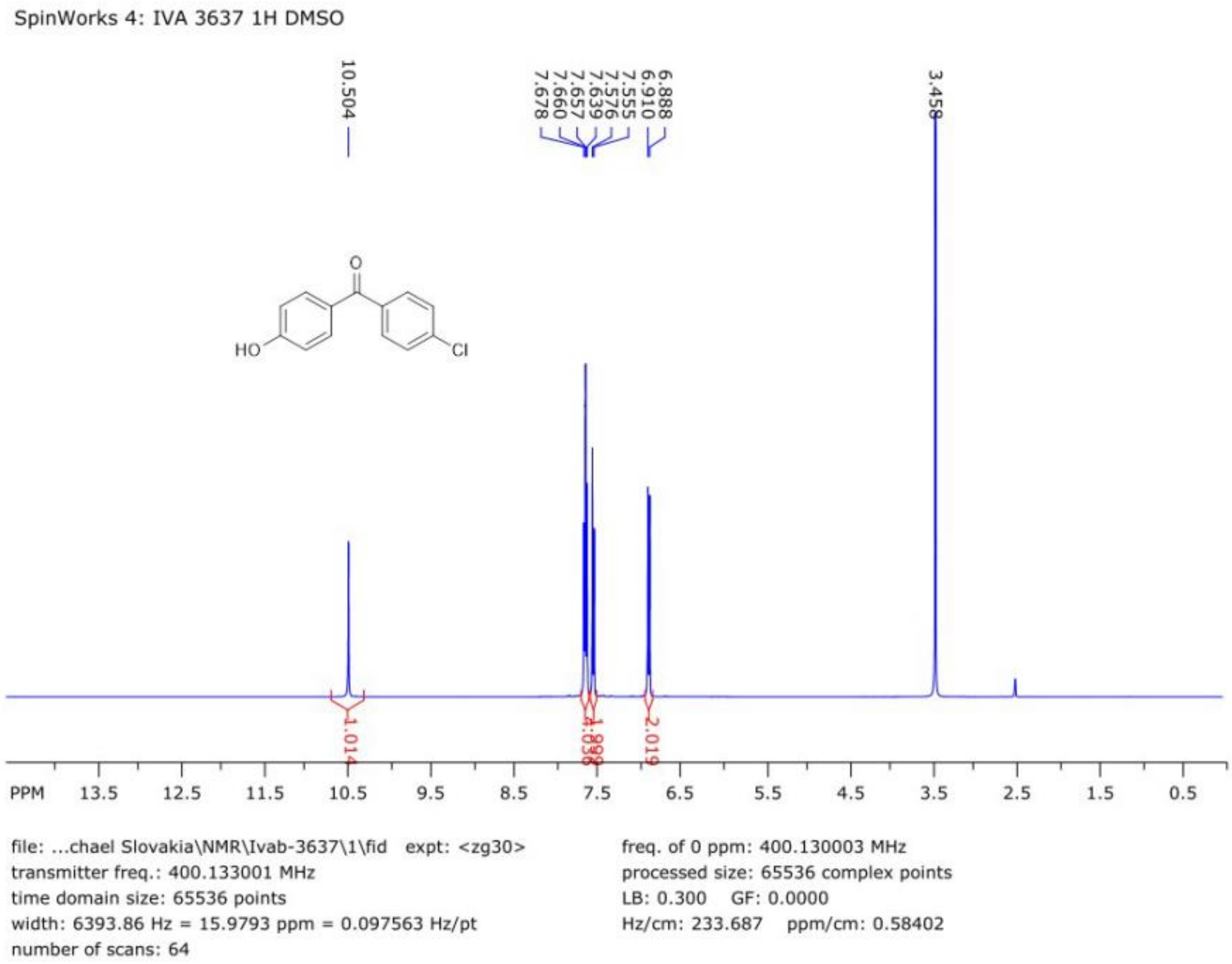

SpinWorks 4: IVA 3637 1H DMSO
10.504
7.678
7.660
7.657
7.639
7.576
7.555
6.910
6.888
3.458
O
HO
Cl
1.014
2.019
PPM 13.5 12.5 11.5 10.5 9.5 8.5 7.5 6.5 5.5 4.5 3.5 2.5 1.5 0.5
file: ...chael Slovakia\NMR\Ivab-3637\1\fid expt: <zg30>
transmitter freq.: 400.133001 MHz
time domain size: 65536 points
width: 6393.86 Hz = 15.9793 ppm = 0.097563 Hz/pt
number of scans: 64
freq. of 0 ppm: 400.130003 MHz
processed size: 65536 complex points
LB: 0.300 GF: 0.0000
Hz/cm: 233.687 ppm/cm: 0.58402

Compound 3o

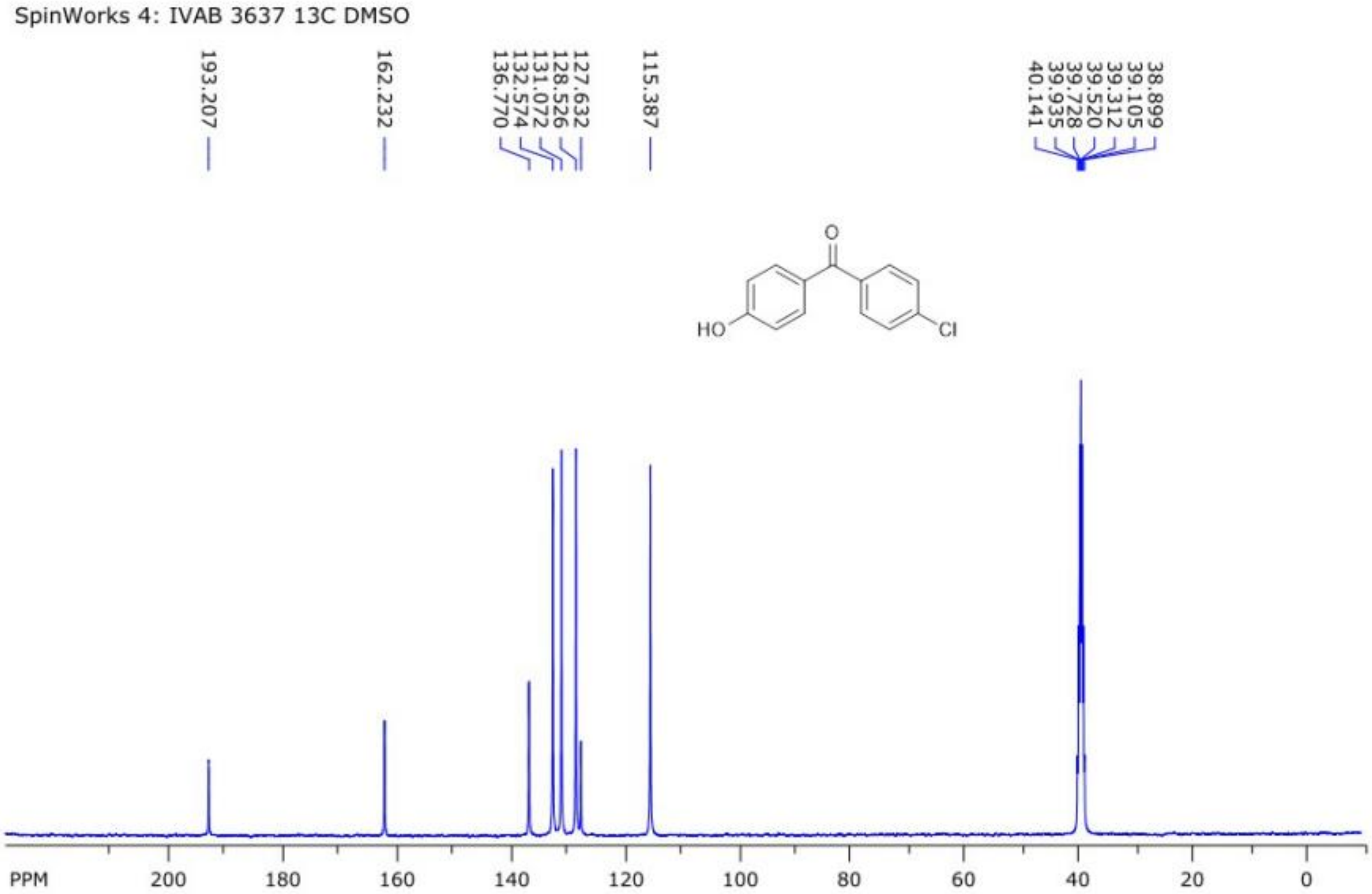

file: ...chael Slovakia\NMR\Ivab-3637\2\fid expt: <zgpg30>
transmitter freq.: 100.623836 MHz
time domain size: 65536 points
width: 24038.46 Hz = 238.8943 ppm = 0.366798 Hz/pt
number of scans: 1291

freq. of 0 ppm: 100.612808 MHz
processed size: 32768 complex points
LB: 10.000 GF: 0.0000
Hz/cm: 961.538 ppm/cm: 9.55577

Compound 3p

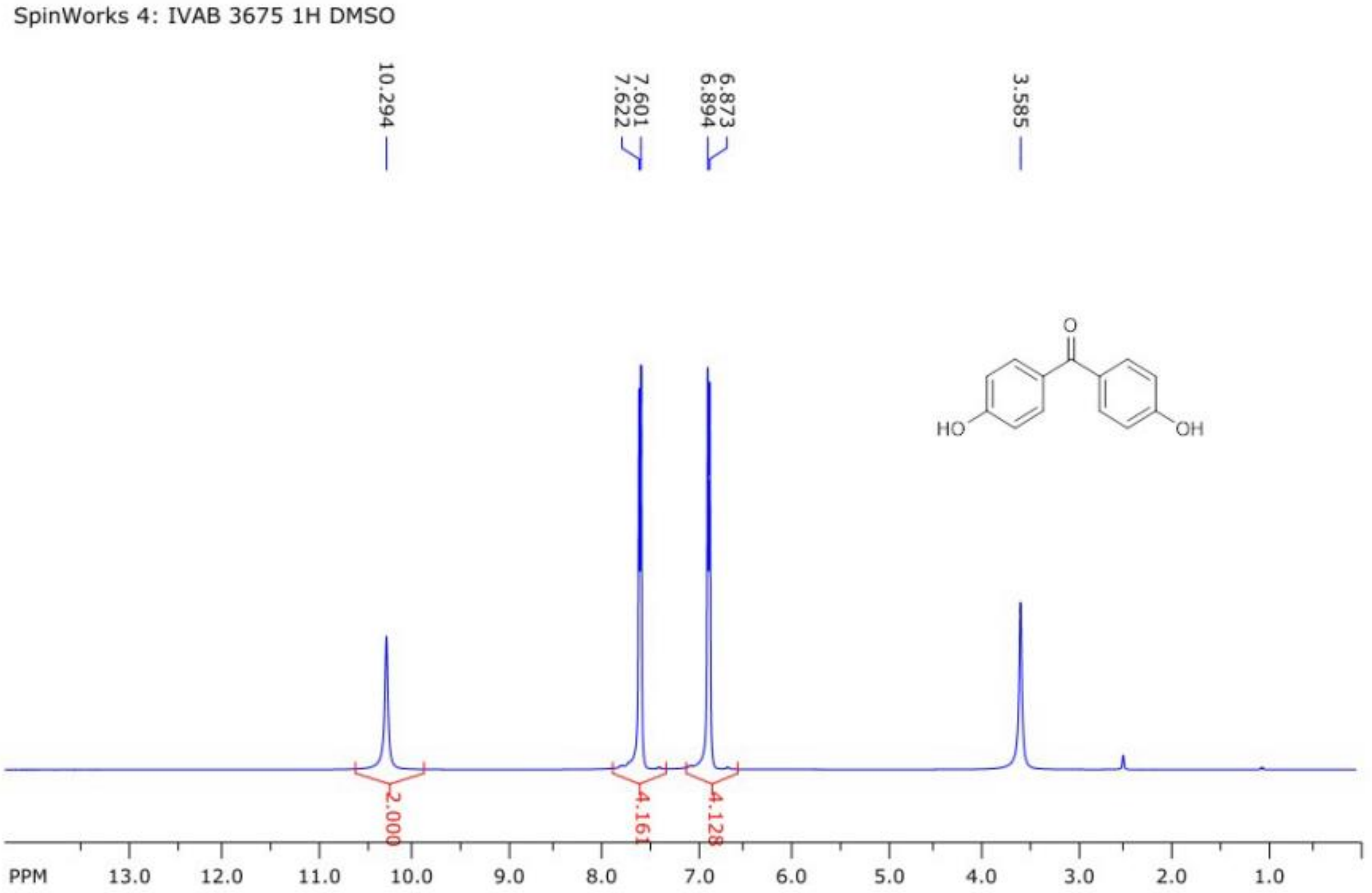

file: ...NMR\Ivab-3675\1H_DMSO_notCDCl3\fid expt: <zg30>
transmitter freq.: 400.133001 MHz
time domain size: 65536 points
width: 6393.86 Hz = 15.9793 ppm = 0.097563 Hz/pt
number of scans: 64

freq. of 0 ppm: 400.130003 MHz
processed size: 65536 complex points
LB: 0.300 GF: 0.0000
Hz/cm: 228.878 ppm/cm: 0.57200

Compound 3p

SpinWorks 4: IVAB 3675 13C DMSO

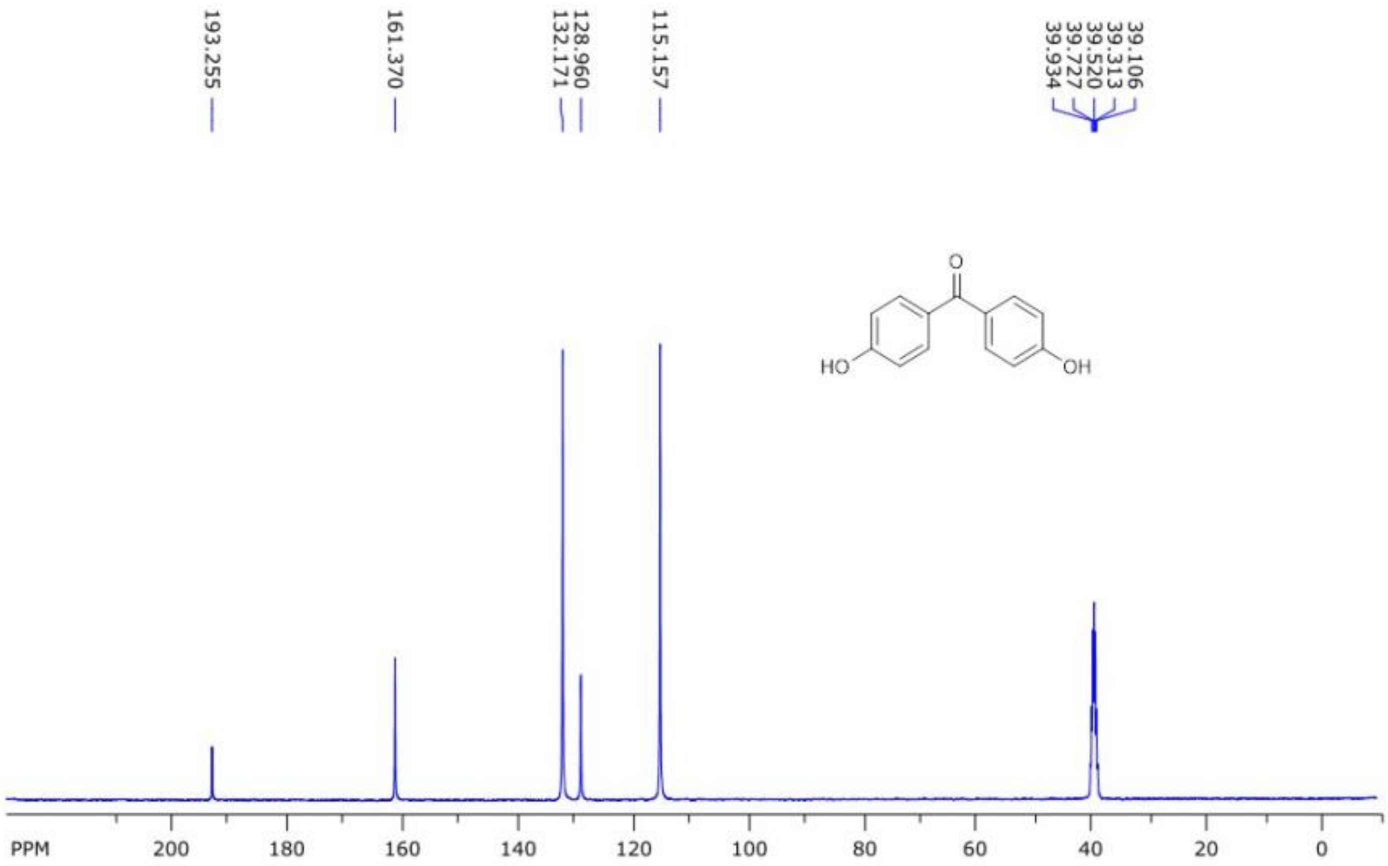

file: ...lovakia\NMR\Ivab-3675\13C_DMSO\fid expt: <zgpg30>
transmitter freq.: 100.623836 MHz
time domain size: 65536 points
width: 24038.46 Hz = 238.8943 ppm = 0.366798 Hz/pt
number of scans: 625

freq. of 0 ppm: 100.612801 MHz
processed size: 32768 complex points
LB: 10.000 GF: 0.0000
Hz/cm: 961.538 ppm/cm: 9.55577

Compound 3q

SpinWorks 4: SVS 396 1H CDCl3

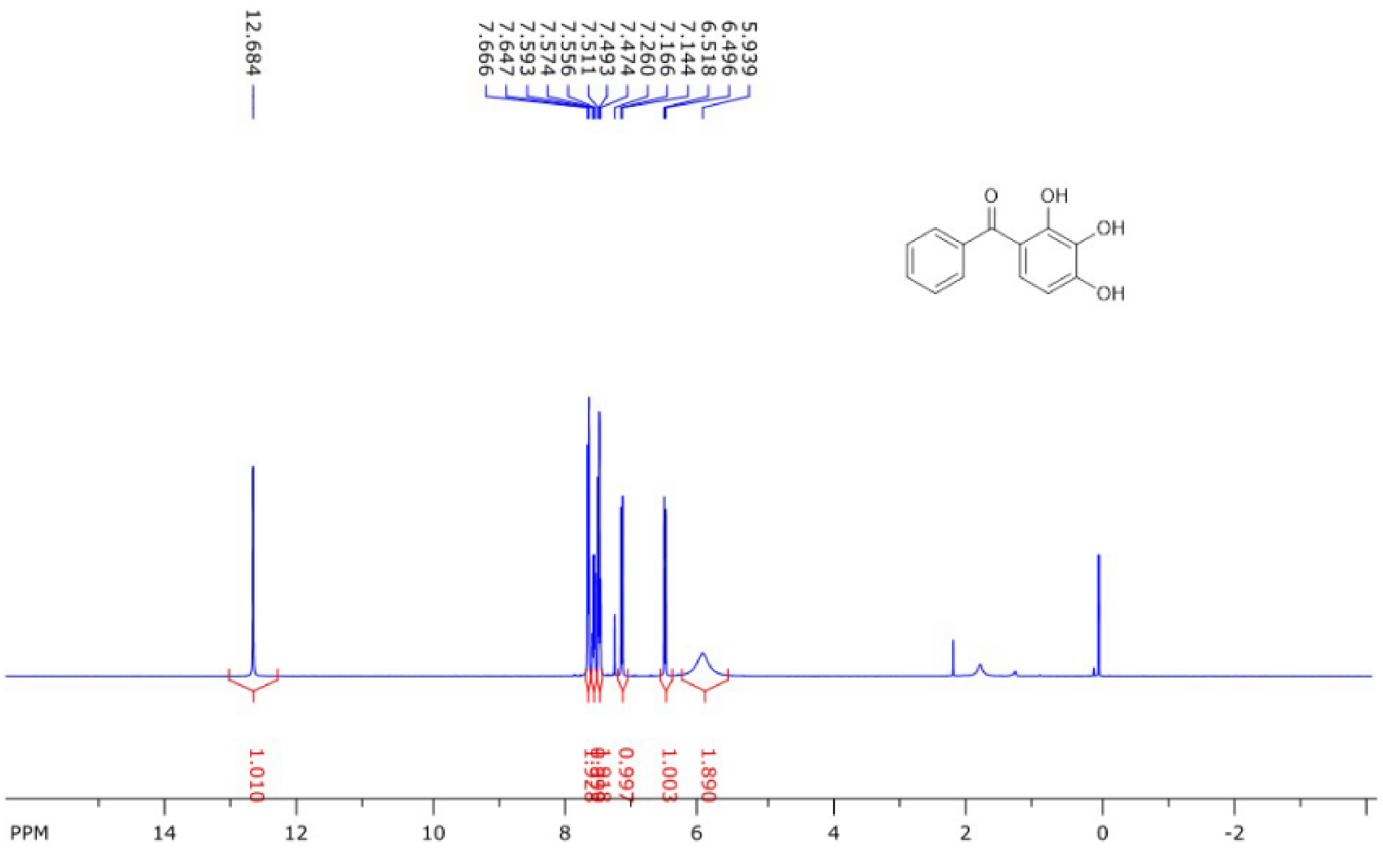

file: ...NAPO\NMR\JELA\nmr\jn-SVS-396\1\fid expt: <zg30>
transmitter freq.: 400.132471 MHz
time domain size: 65536 points
width: 8196.72 Hz = 20.4850 ppm = 0.125072 Hz/pt
number of scans: 16

freq. of 0 ppm: 400.130009 MHz
processed size: 65536 complex points
LB: 0.300 GF: 0.0000
Hz/cm: 327.869 ppm/cm: 0.81940

Compound 3q

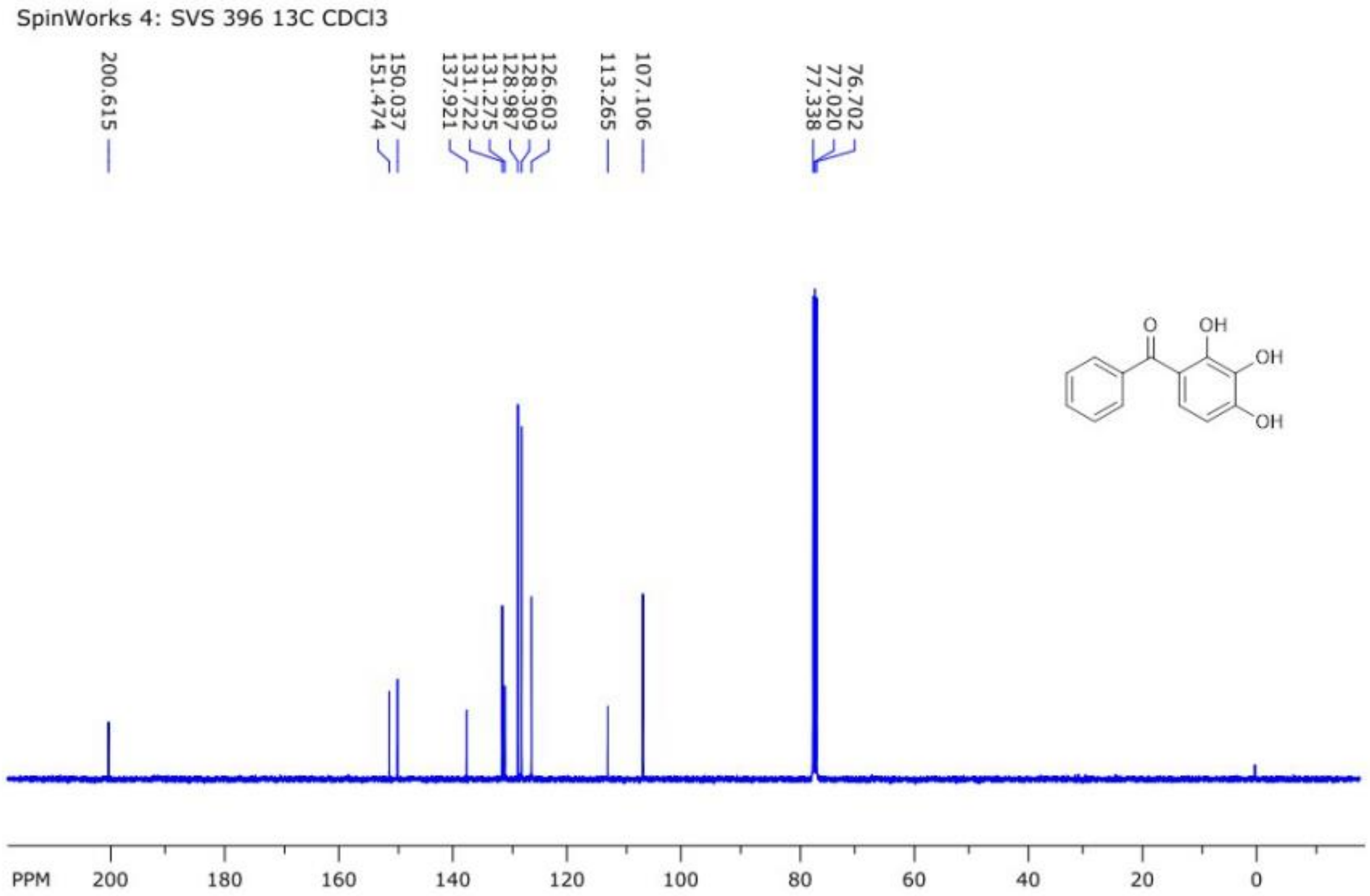

file: ...NAPO\NMR\JELA\nmr\jn-SVS-396\2\fid expt: <zgpg30>
transmitter freq.: 100.622830 MHz
time domain size: 65536 points
width: 23809.52 Hz = 236.6215 ppm = 0.363305 Hz/pt
number of scans: 1024

freq. of 0 ppm: 100.612768 MHz
processed size: 32768 complex points
LB: 1.000 GF: 0.0000
Hz/cm: 952.381 ppm/cm: 9.46486

Compound 3r

SpinWorks 4: SVS 400 1H CDCl3

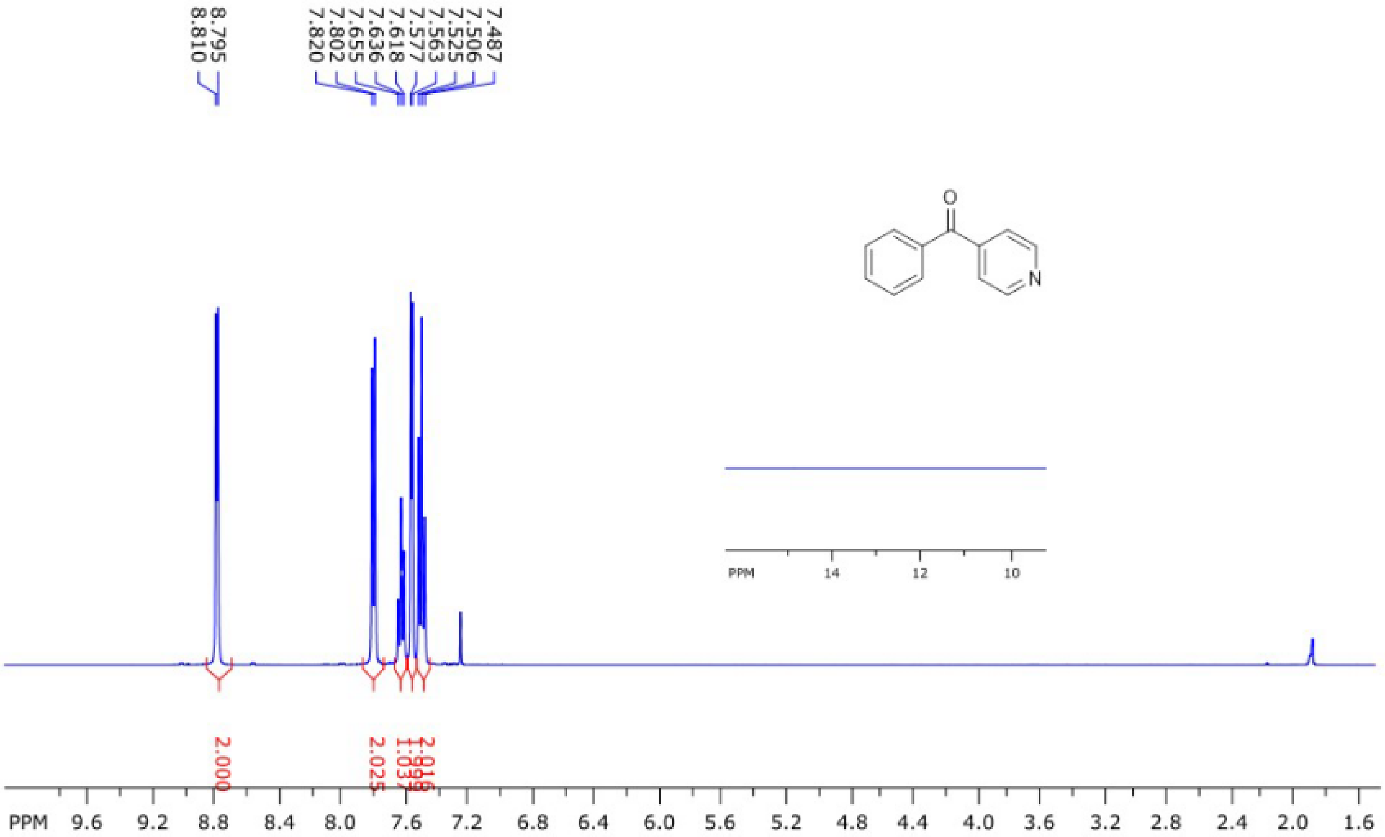

file: ...NAPO\NMR\JELA\nmr\jn-SVS-400\1\fid expt: <zg30>
transmitter freq.: 400.132471 MHz
time domain size: 65536 points
width: 8196.72 Hz = 20.4850 ppm = 0.125072 Hz/pt
number of scans: 16

freq. of 0 ppm: 400.130009 MHz
processed size: 65536 complex points
LB: 0.300 GF: 0.0000
Hz/cm: 138.944 ppm/cm: 0.34724

Compound 3r

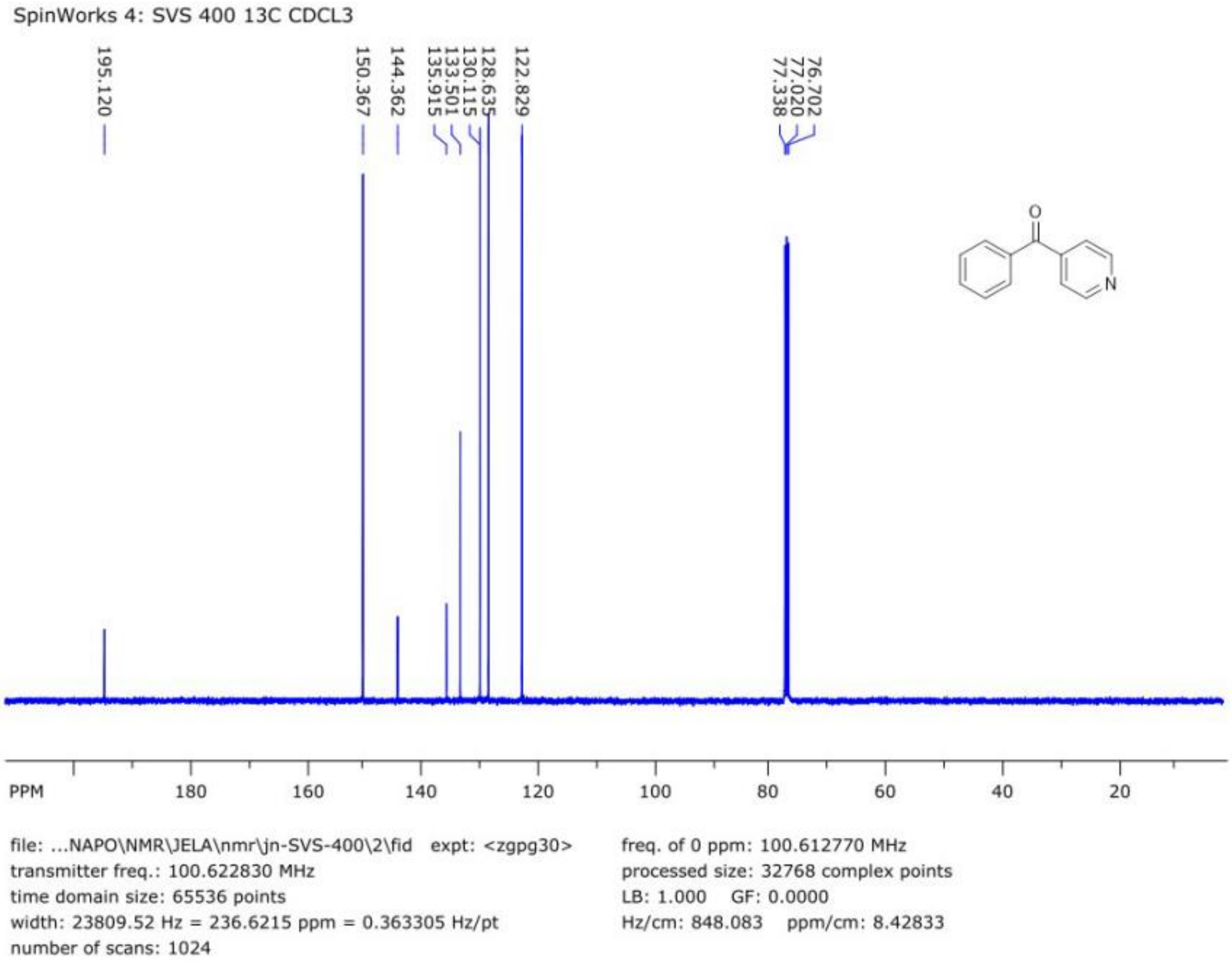
SpinWorks 4: SVS 400 13C CDCL3
195.120
150.367
144.362
135.915
133.501
130.115
128.635
122.829
77.338
77.020
76.702
N
O
PPM
180
160
140
120
100
80
60
40
20
file: ...NAPO\NMR\JELA\nmr\jn-SVS-400\2\fid expt: <zgpg30>
transmitter freq.: 100.622830 MHz
time domain size: 65536 points
width: 23809.52 Hz = 236.6215 ppm = 0.363305 Hz/pt
number of scans: 1024
freq. of 0 ppm: 100.612770 MHz
processed size: 32768 complex points
LB: 1.000 GF: 0.0000
Hz/cm: 848.083 ppm/cm: 8.42833

Compound 3s

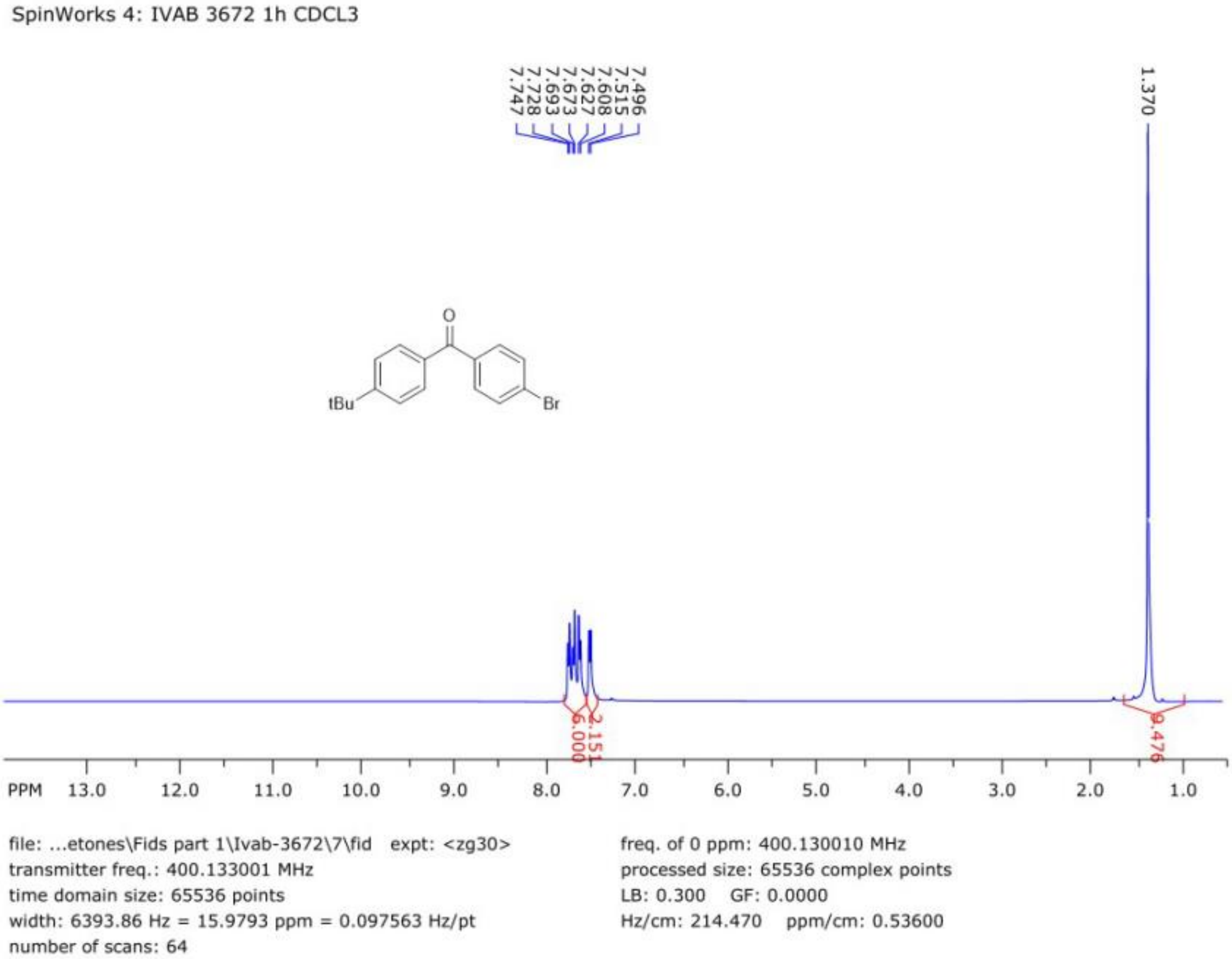
SpinWorks 4: IVAB 3672 1h CDCL3
7.747
7.728
7.693
7.673
7.627
7.608
7.515
7.496
1.370
O
tBu
Br
6.000
2.151
9.476
PPM 13.0 12.0 11.0 10.0 9.0 8.0 7.0 6.0 5.0 4.0 3.0 2.0 1.0
file: ...etones\Fids part 1\Ivab-3672\7\fid expt: <zg30>
transmitter freq.: 400.133001 MHz
time domain size: 65536 points
width: 6393.86 Hz = 15.9793 ppm = 0.097563 Hz/pt
number of scans: 64
freq. of 0 ppm: 400.130010 MHz
processed size: 65536 complex points
LB: 0.300 GF: 0.0000
Hz/cm: 214.470 ppm/cm: 0.53600

Compound 3s

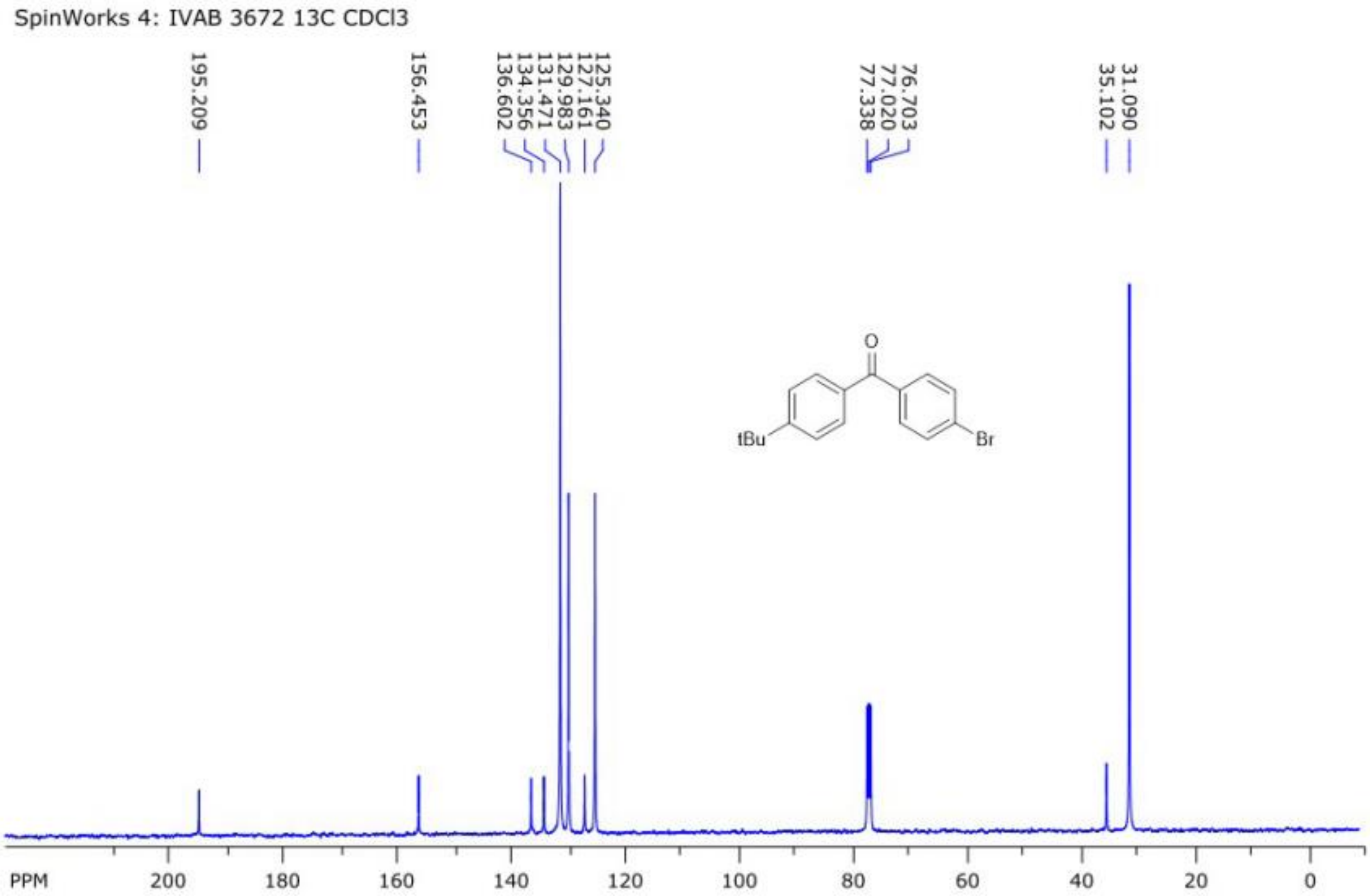

file: ...etones\Fids part 1\Ivab-3672\8\fid expt: <zgpg30>
transmitter freq.: 100.623836 MHz
time domain size: 65536 points
width: 24038.46 Hz = 238.8943 ppm = 0.366798 Hz/pt
number of scans: 512

freq. of 0 ppm: 100.612774 MHz
processed size: 32768 complex points
LB: 10.000 GF: 0.0000
Hz/cm: 961.538 ppm/cm: 9.55577

Compound 3t

SpinWorks 4: IVA 2207 1H CDCl3

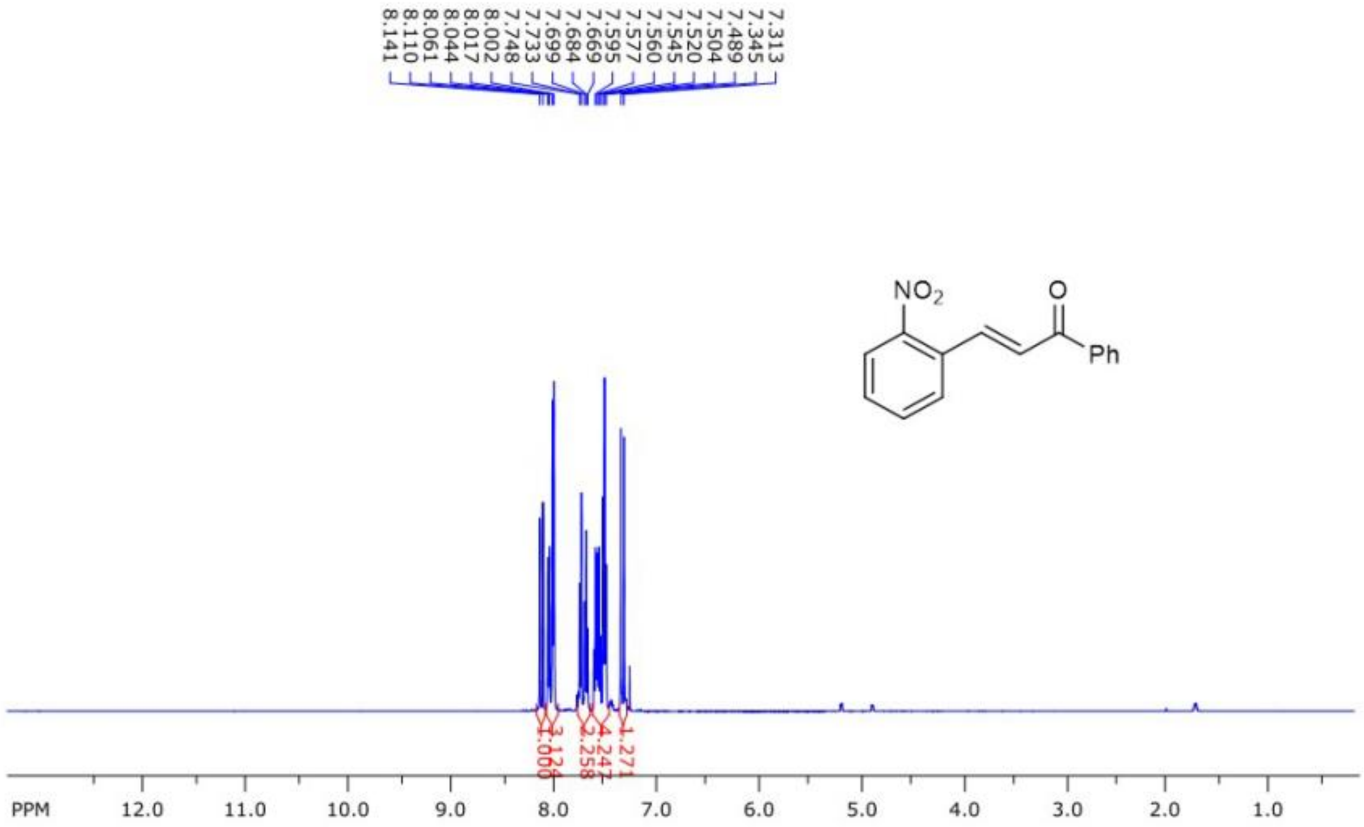

file: ...etones\Fids part 1\IVA 2207\27\fid expt: <zg30>
transmitter freq.: 500.133001 MHz
time domain size: 65536 points
width: 12335.53 Hz = 24.6645 ppm = 0.188225 Hz/pt
number of scans: 24

freq. of 0 ppm: 500.130023 MHz
processed size: 65536 complex points
LB: 0.300 GF: 0.0000
Hz/cm: 264.509 ppm/cm: 0.52888

Compound 3t

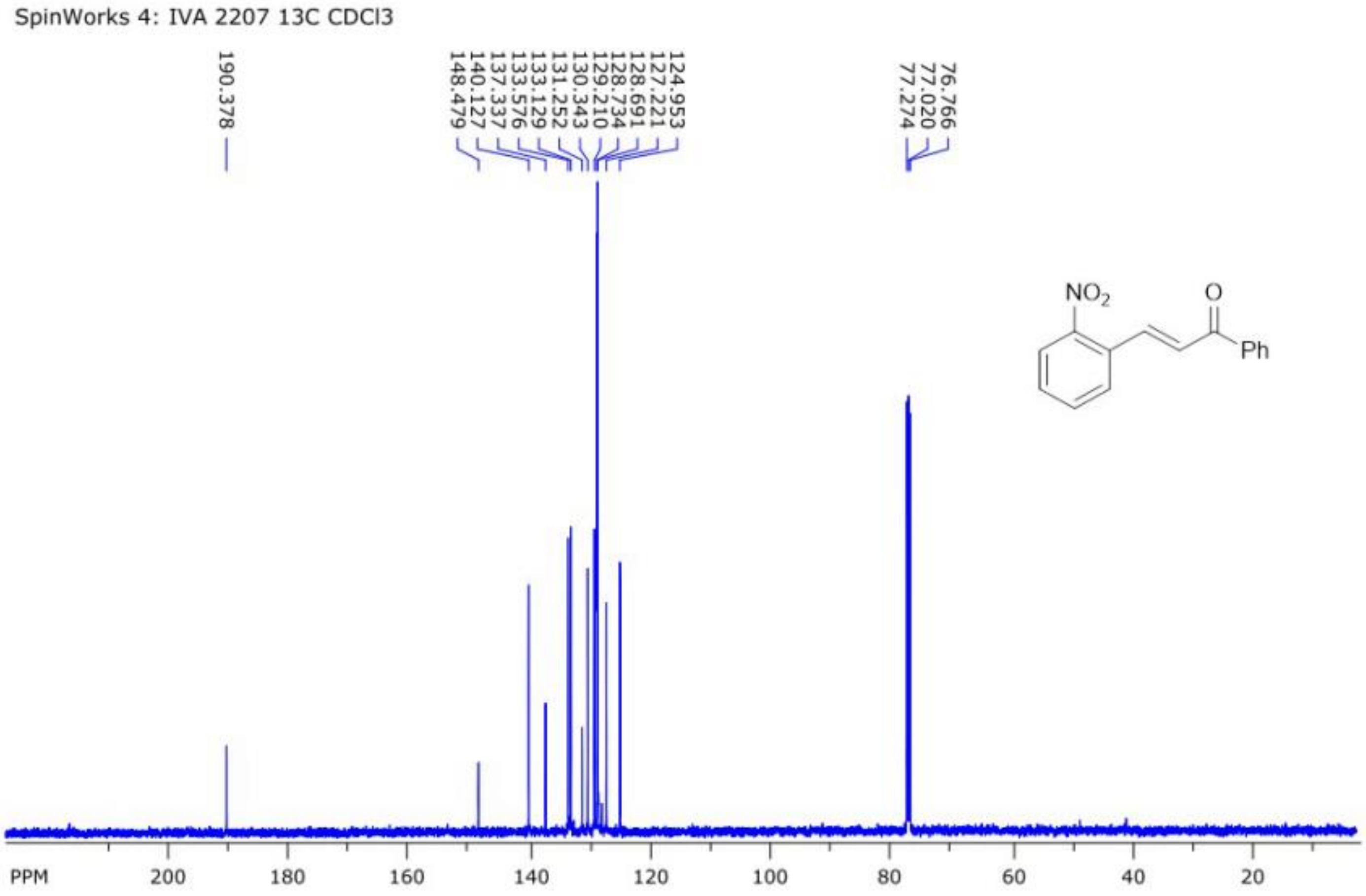

file: ...etones\Fids part 1\IVA 2207\28\fid expt: <zgpg30>
transmitter freq.: 125.772879 MHz
time domain size: 65536 points
width: 36057.69 Hz = 286.6889 ppm = 0.550197 Hz/pt
number of scans: 512

freq. of 0 ppm: 125.757798 MHz
processed size: 32768 complex points
LB: 2.000 GF: 0.0000
Hz/cm: 1128.700 ppm/cm: 8.97411

Compound 3u

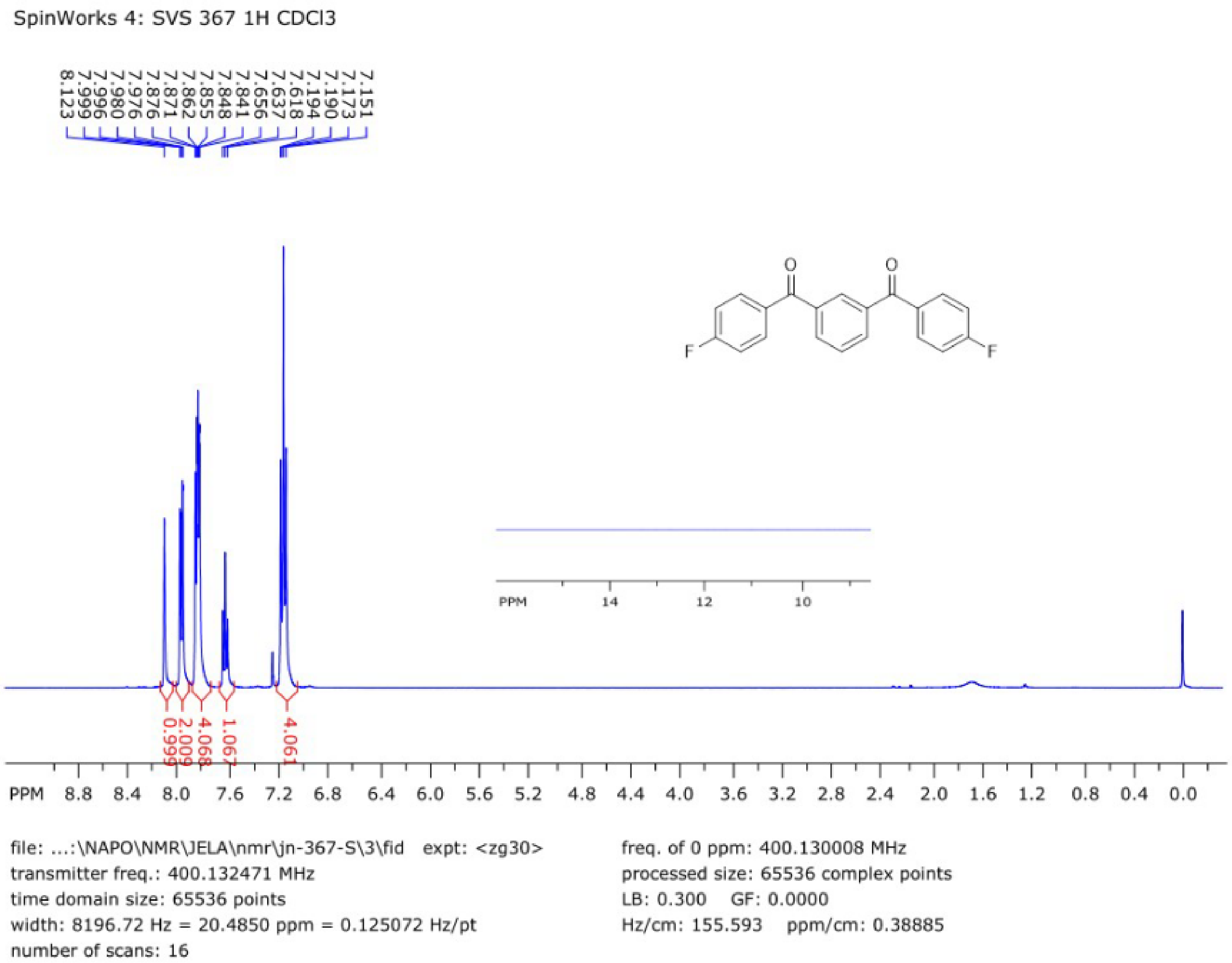
SpinWorks 4: SVS 367 1H CDCl3
8.123
7.999
7.996
7.980
7.976
7.876
7.871
7.862
7.855
7.848
7.841
7.656
7.637
7.618
7.194
7.190
7.173
7.151
0.999
2.009
4.068
1.067
4.061
PPM 8.8 8.4 8.0 7.6 7.2 6.8 6.4 6.0 5.6 5.2 4.8 4.4 4.0 3.6 3.2 2.8 2.4 2.0 1.6 1.2 0.8 0.4 0.0
PPM 14 12 10
O
O
F
F
file: ...:\NAPO\NMR\JELA\nmr\jn-367-S\3\fid expt: <zg30>
transmitter freq.: 400.132471 MHz
time domain size: 65536 points
width: 8196.72 Hz = 20.4850 ppm = 0.125072 Hz/pt
number of scans: 16
freq. of 0 ppm: 400.130008 MHz
processed size: 65536 complex points
LB: 0.300 GF: 0.0000
Hz/cm: 155.593 ppm/cm: 0.38885

Compound 3u

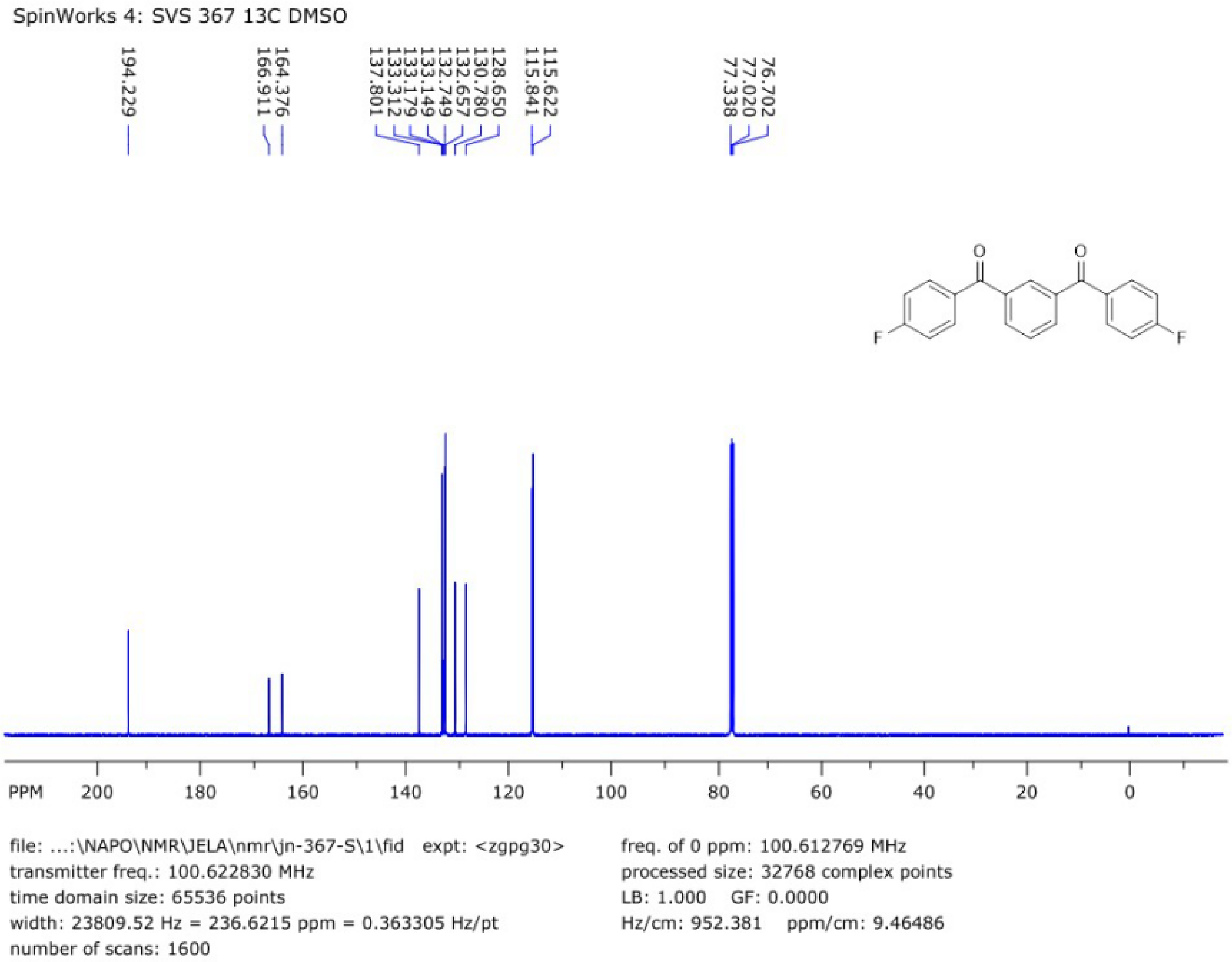

SpinWorks 4: SVS 367 13C DMSO
194.229
166.911
164.376
137.801
133.312
133.179
133.149
132.749
132.657
130.780
128.650
115.841
115.622
77.338
77.020
76.702
O
O
F
F
PPM 200 180 160 140 120 100 80 60 40 20 0
file: ...:\NAPO\NMR\JELA\nmr\jn-367-S\1\fid expt: <zgpg30>
transmitter freq.: 100.622830 MHz
time domain size: 65536 points
width: 23809.52 Hz = 236.6215 ppm = 0.363305 Hz/pt
number of scans: 1600
freq. of 0 ppm: 100.612769 MHz
processed size: 32768 complex points
LB: 1.000 GF: 0.0000
Hz/cm: 952.381 ppm/cm: 9.46486

Compound 3v

SpinWorks 4:

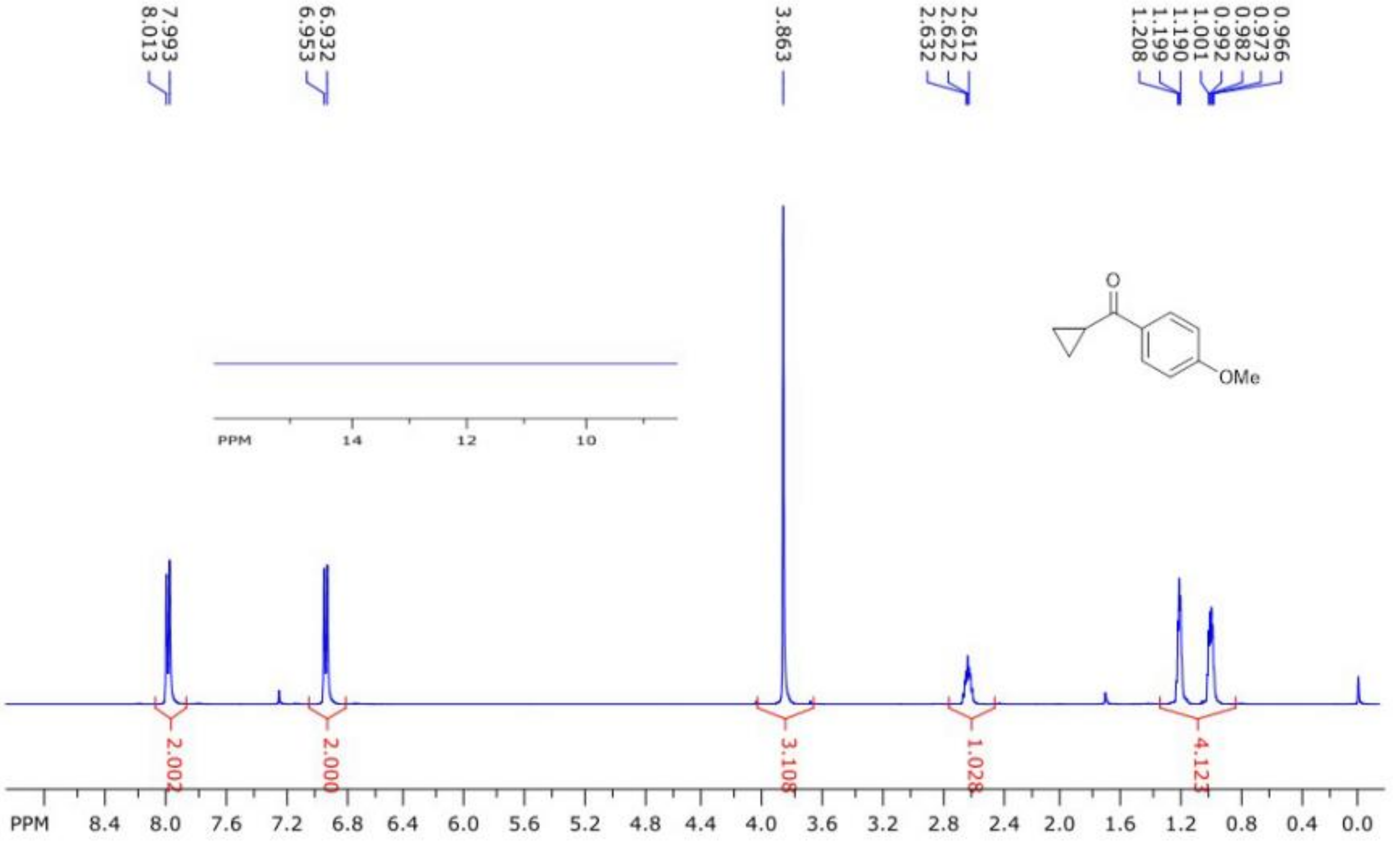

file: D:\NAPO\NMR\JELA\nmr\jn-368\2\fid expt: <zg30>
transmitter freq.: 400.132471 MHz
time domain size: 65536 points
width: 8196.72 Hz = 20.4850 ppm = 0.125072 Hz/pt
number of scans: 16

freq. of 0 ppm: 400.130010 MHz
processed size: 65536 complex points
LB: 0.300 GF: 0.0000
Hz/cm: 147.976 ppm/cm: 0.36982

Compound 3v

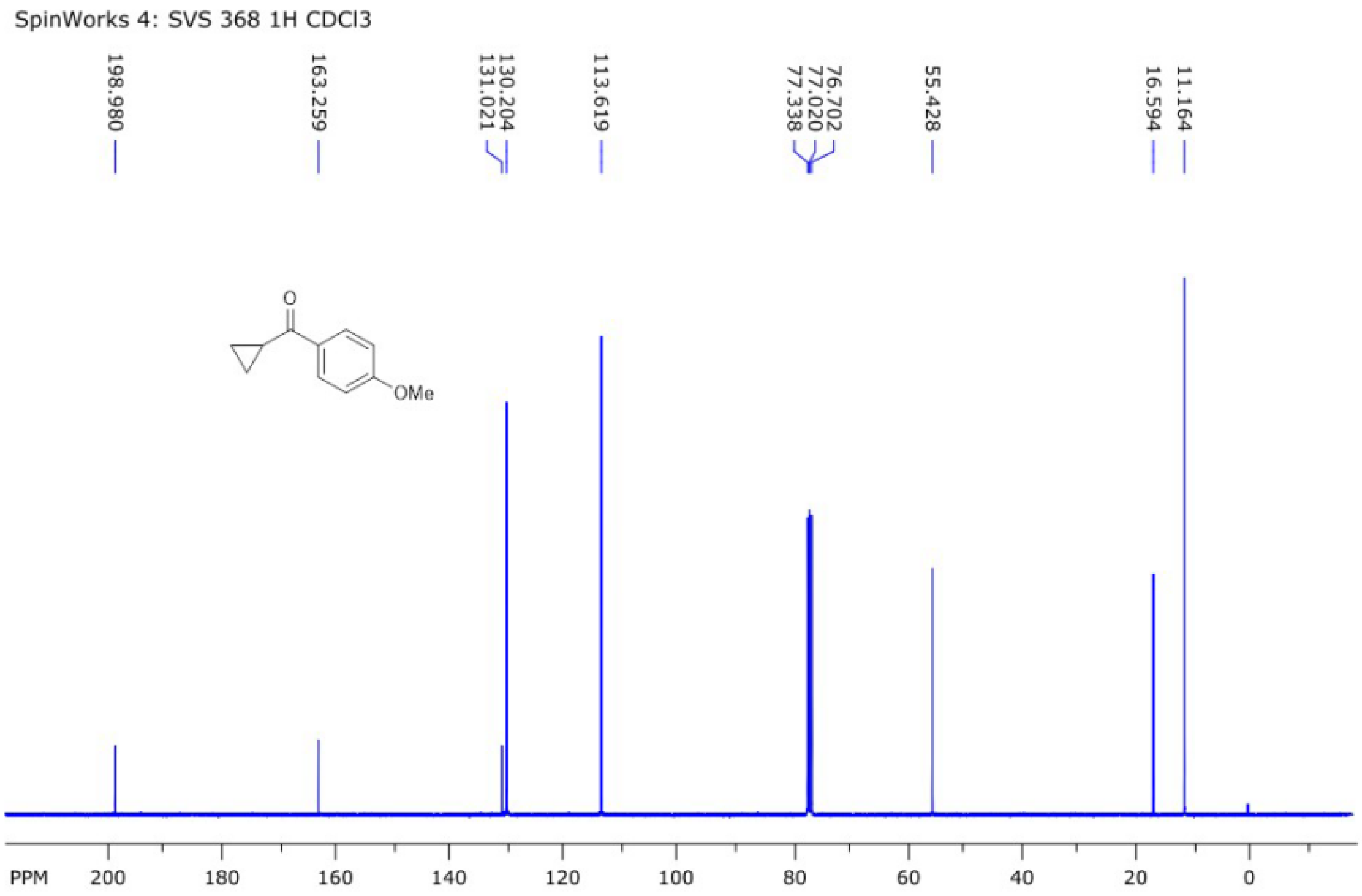

file: D:\NAPO\NMR\JELA\nmr\jn-368\1\fid expt: <zgpg30>
transmitter freq.: 100.622830 MHz
time domain size: 65536 points
width: 23809.52 Hz = 236.6215 ppm = 0.363305 Hz/pt
number of scans: 1024

freq. of 0 ppm: 100.612770 MHz
processed size: 32768 complex points
LB: 1.000 GF: 0.0000
Hz/cm: 952.381 ppm/cm: 9.46486

Compound 3w

SpinWorks 4: SVS 258 1H DMSO

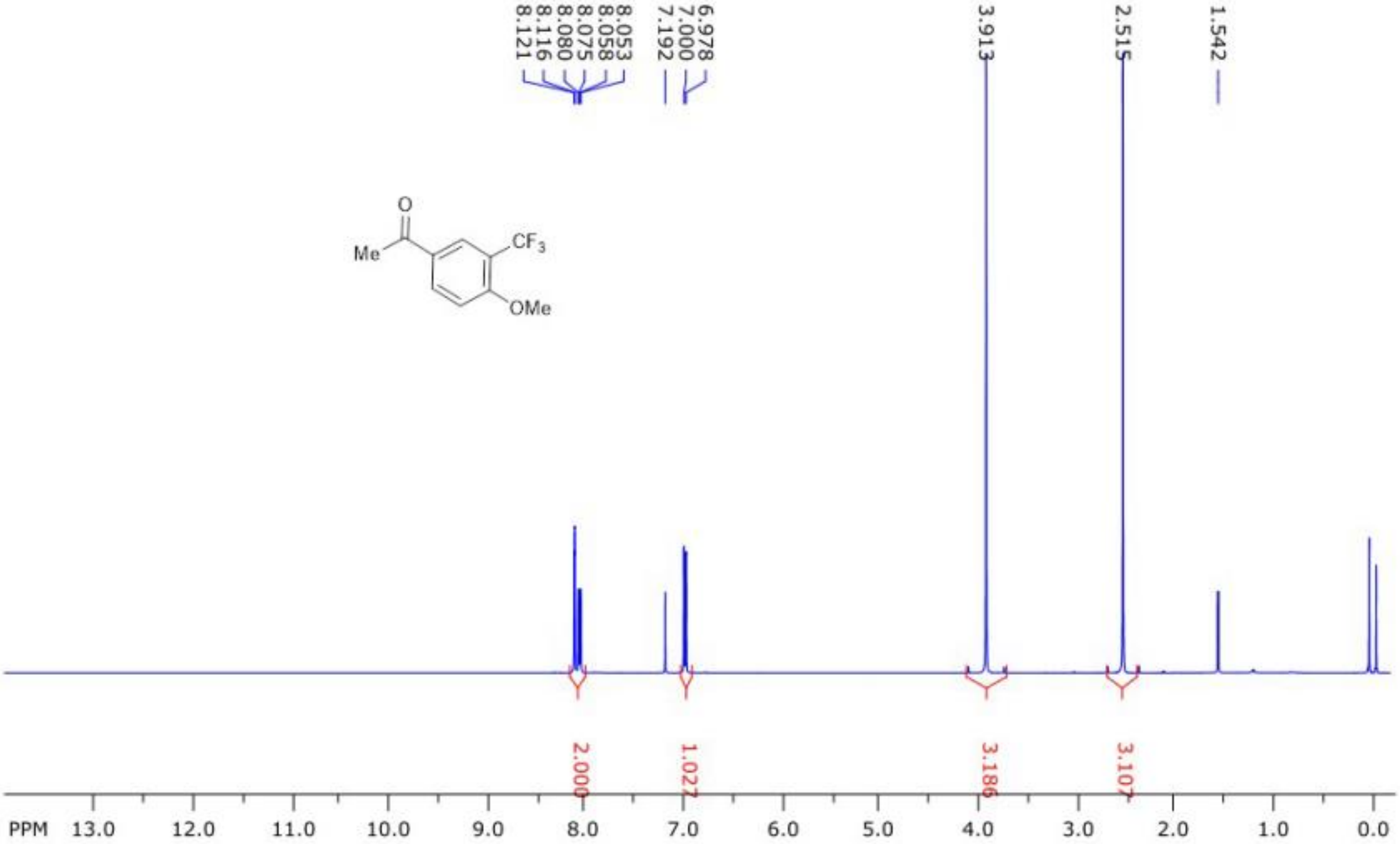

file: ... ketones\Fids part 1\SVS 258\1\fid expt: <zg30>
transmitter freq.: 400.132471 MHz
time domain size: 65536 points
width: 8196.72 Hz = 20.4850 ppm = 0.125072 Hz/pt
number of scans: 16

freq. of 0 ppm: 400.130036 MHz
processed size: 65536 complex points
LB: 0.300 GF: 0.0000
Hz/cm: 226.683 ppm/cm: 0.56652

Compound 3w

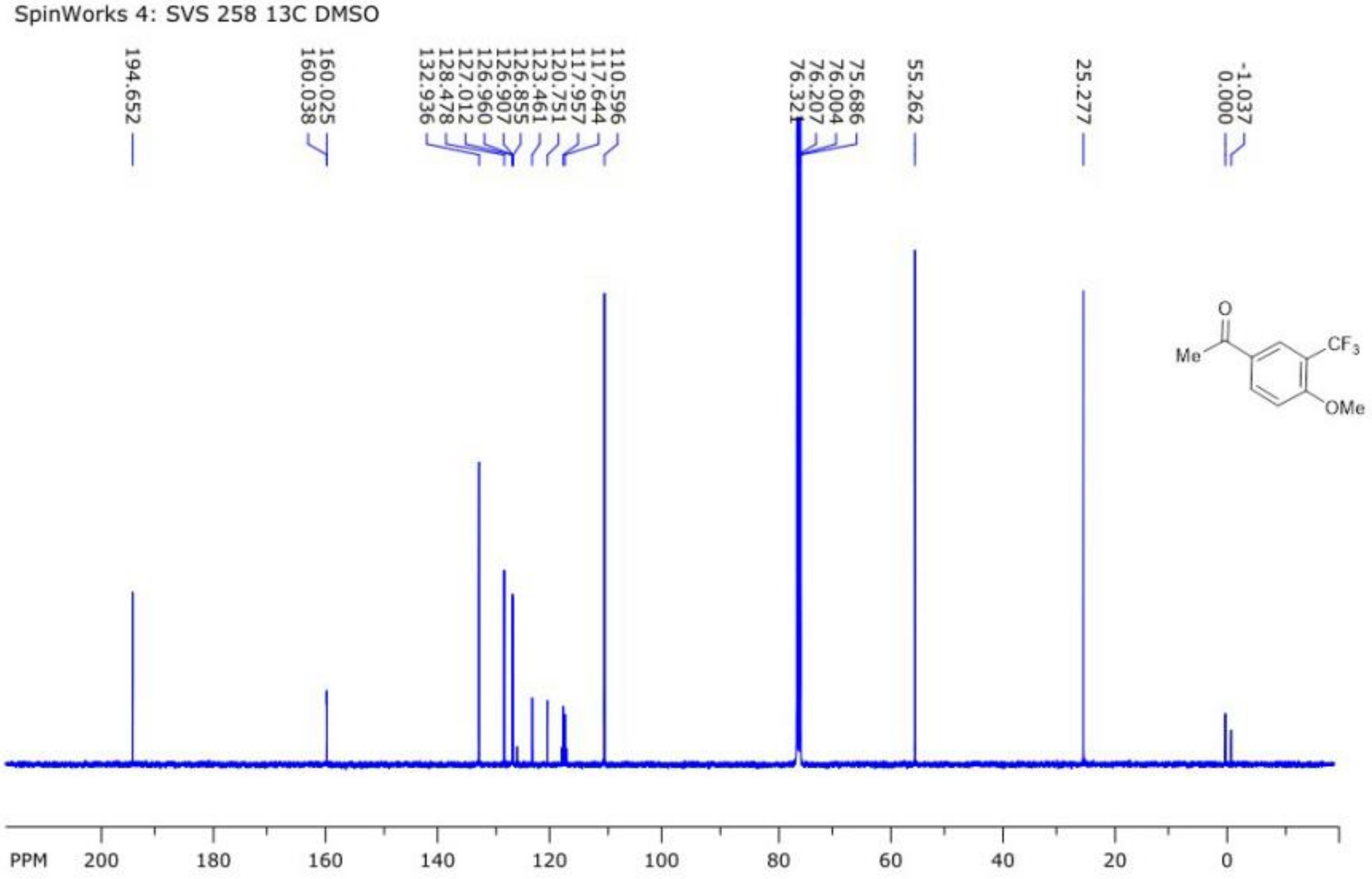

file: ... ketones\Fids part 1\SVS 258\2\fid expt: <zgpg30>
transmitter freq.: 100.622830 MHz
time domain size: 65536 points
width: 23809.52 Hz = 236.6215 ppm = 0.363305 Hz/pt
number of scans: 5000

freq. of 0 ppm: 100.612871 MHz
processed size: 32768 complex points
LB: 1.000 GF: 0.0000
Hz/cm: 952.381 ppm/cm: 9.46486

Compound 3x

SpinWorks 4: IVAB 3642 1H CDCl3

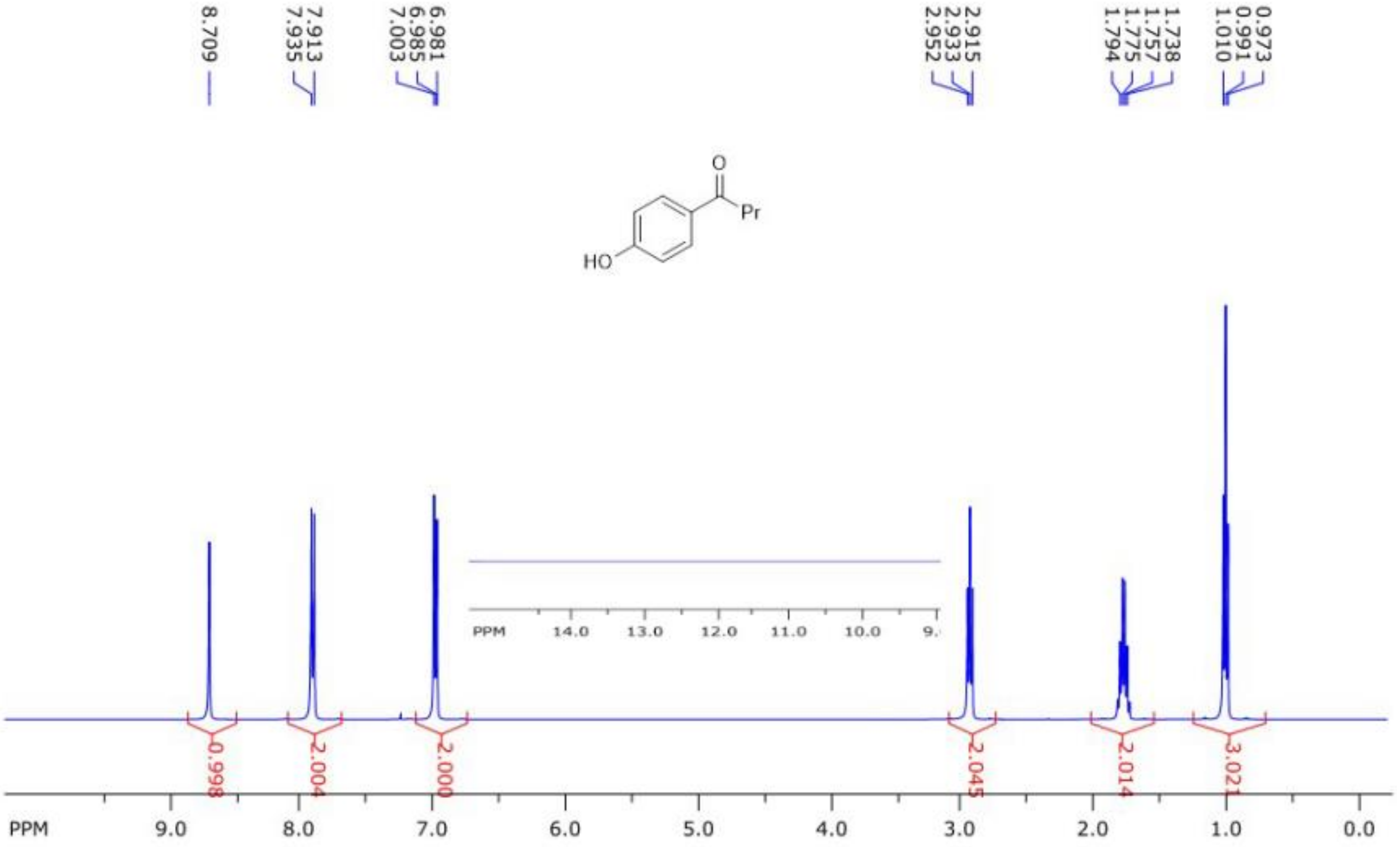

file: ...chael Slovakia\NMR\Ivab-3642\3\fid expt: <zg30>
transmitter freq.: 400.133001 MHz
time domain size: 65536 points
width: 6393.86 Hz = 15.9793 ppm = 0.097563 Hz/pt
number of scans: 64

freq. of 0 ppm: 400.130011 MHz
processed size: 65536 complex points
LB: 0.300 GF: 0.0000
Hz/cm: 168.051 ppm/cm: 0.41999

Compound 3x

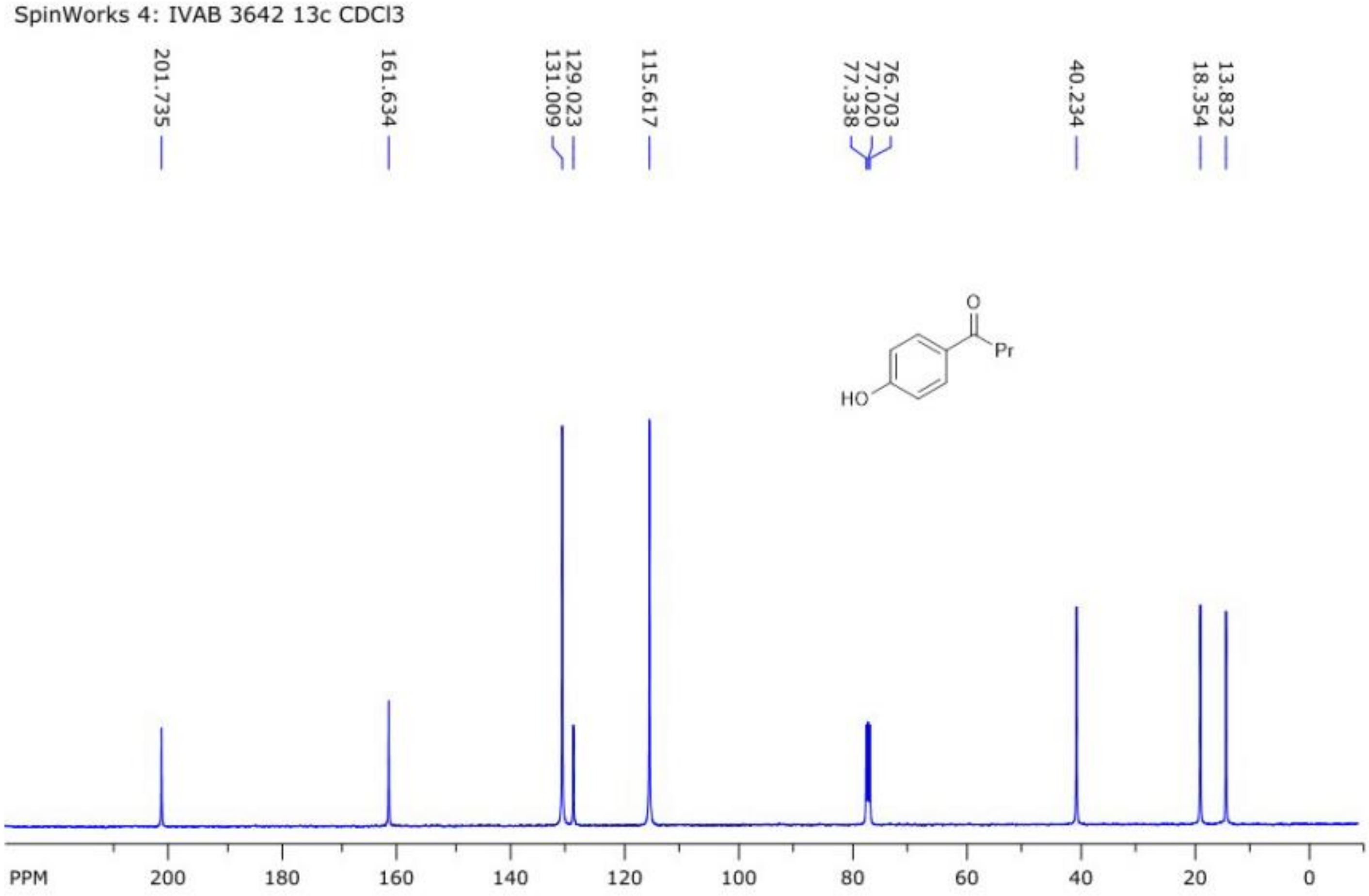

file: ...chael Slovakia\NMR\Ivab-3642\4\fid expt: <zgpg30>
transmitter freq.: 100.623836 MHz
time domain size: 65536 points
width: 24038.46 Hz = 238.8943 ppm = 0.366798 Hz/pt
number of scans: 1122

freq. of 0 ppm: 100.612777 MHz
processed size: 32768 complex points
LB: 10.000 GF: 0.0000
Hz/cm: 961.538 ppm/cm: 9.55577

Compound 3y

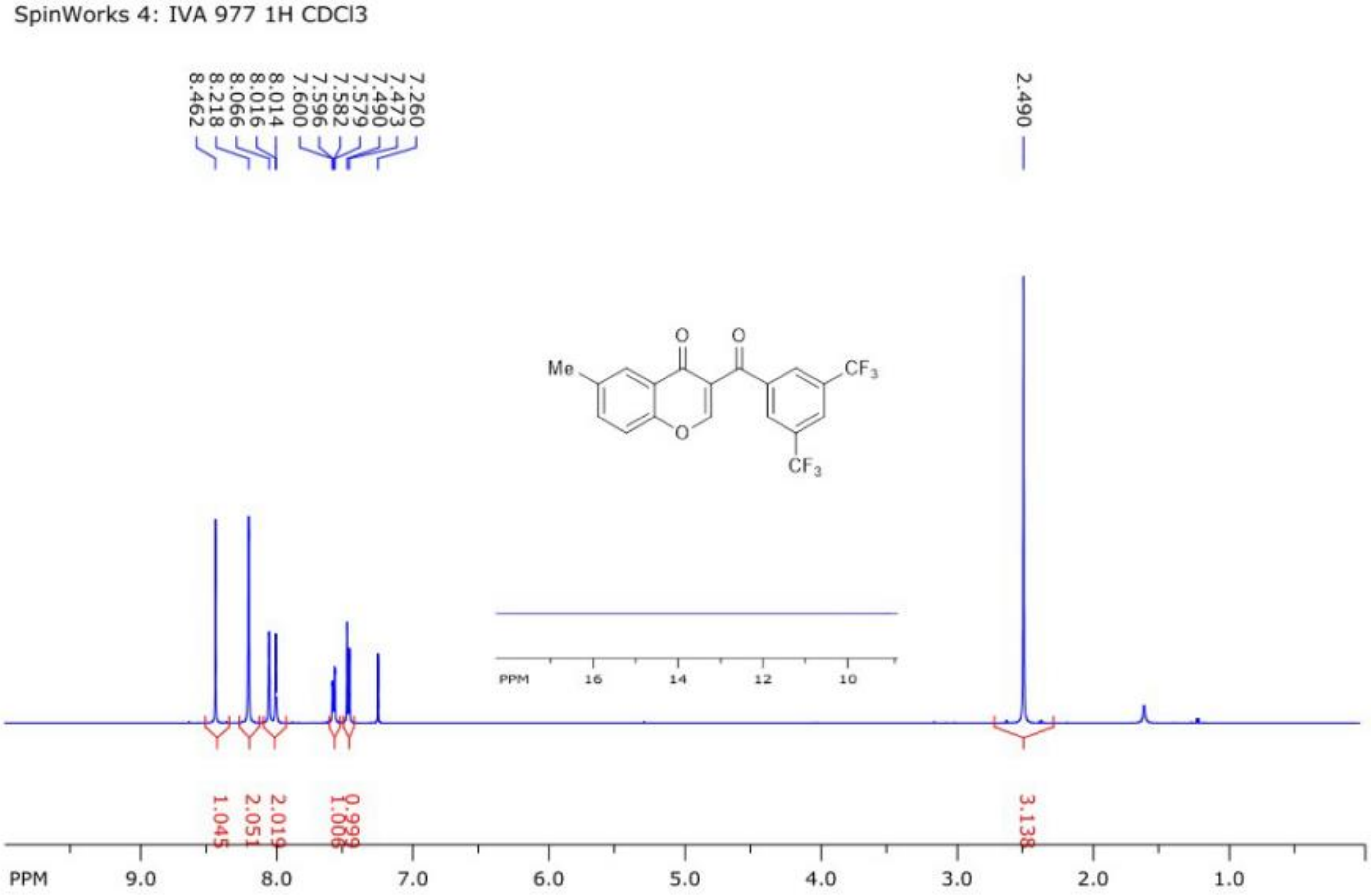

file: D:\NAPO\NMR\500-1\mkr11405\23\fid expt: <zg30>
transmitter freq.: 500.133001 MHz
time domain size: 65536 points
width: 12335.53 Hz = 24.6645 ppm = 0.188225 Hz/pt
number of scans: 24

freq. of 0 ppm: 500.130023 MHz
processed size: 65536 complex points
LB: 0.300 GF: 0.0000
Hz/cm: 200.316 ppm/cm: 0.40053

Compound 3y

SpinWorks 4: IVA 977 13C CDCl3

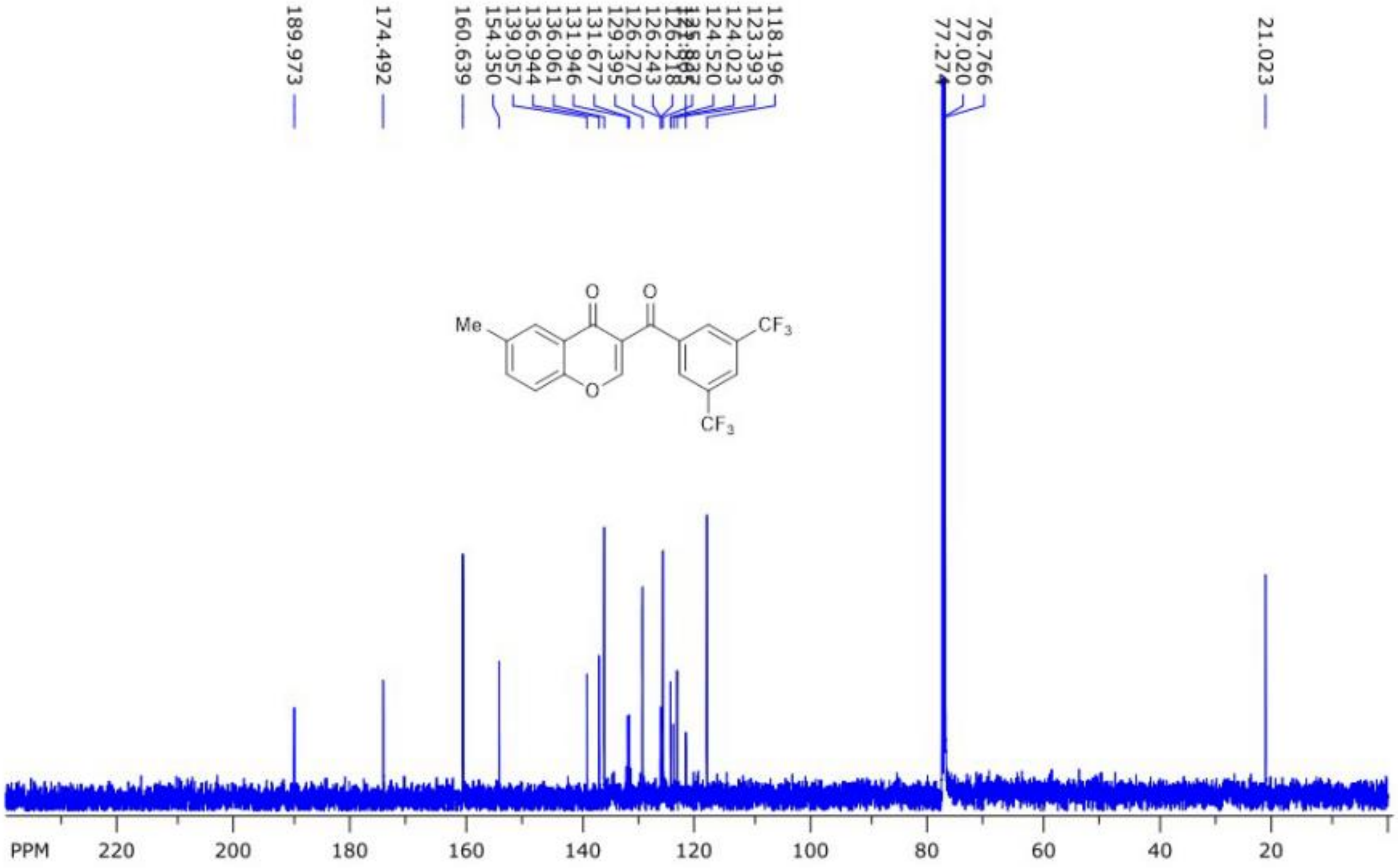

file: D:\NAPO\NMR\500-1\mkr11405\24\fid expt: <zgpg30>
transmitter freq.: 125.772879 MHz
time domain size: 65536 points
width: 36057.69 Hz = 286.6889 ppm = 0.550197 Hz/pt
number of scans: 256

freq. of 0 ppm: 125.757792 MHz
processed size: 32768 complex points
LB: 2.000 GF: 0.0000
Hz/cm: 1209.369 ppm/cm: 9.61550

Compound 3z

SpinWorks 4: IVA 1033 1H CDCl3

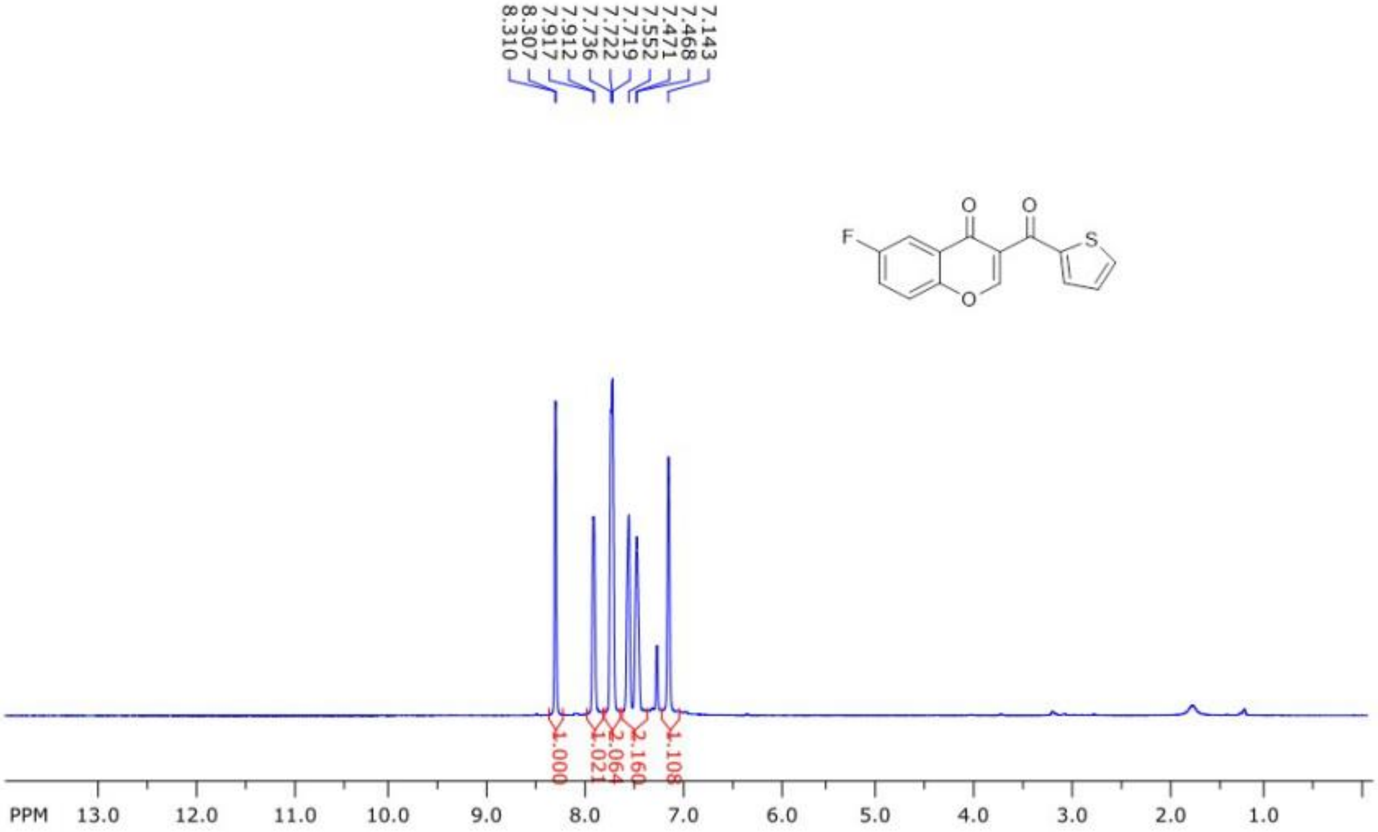

file: ...ketones\Fids part 1\IVA 1033\1\fid expt: <zg30>
transmitter freq.: 500.133001 MHz
time domain size: 65536 points
width: 12335.53 Hz = 24.6645 ppm = 0.188225 Hz/pt
number of scans: 32

freq. of 0 ppm: 500.130021 MHz
processed size: 65536 complex points
LB: 0.300 GF: 0.0000
Hz/cm: 281.319 ppm/cm: 0.56249

Compound 3z

SpinWorks 4: IVA 1033 13C CDCl3

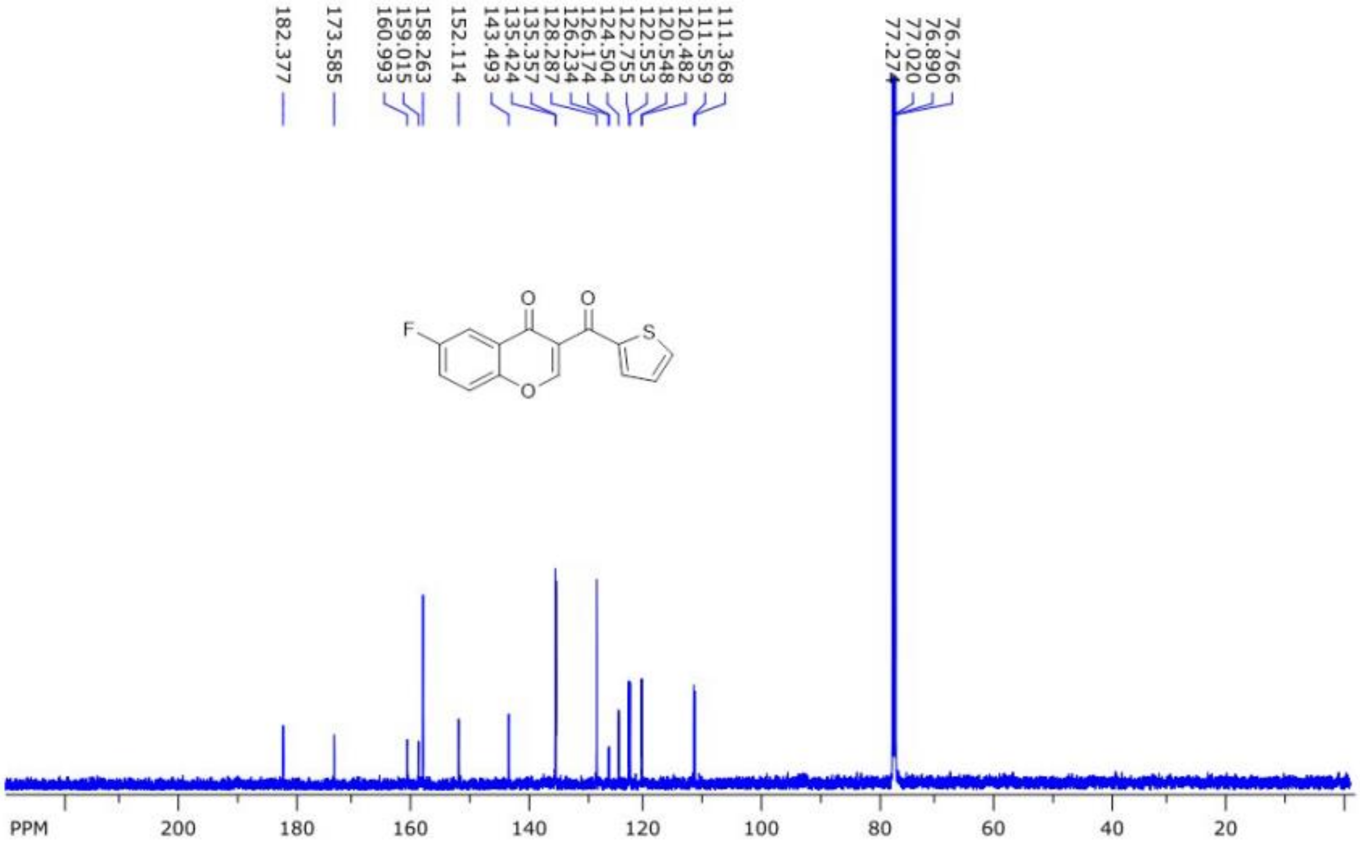

file: ...ketones\Fids part 1\IVA 1033\2\fid expt: <zgpg30>
transmitter freq.: 125.772879 MHz
time domain size: 65536 points
width: 36057.69 Hz = 286.6889 ppm = 0.550197 Hz/pt
number of scans: 512

freq. of 0 ppm: 125.757796 MHz
processed size: 32768 complex points
LB: 2.000 GF: 0.0000
Hz/cm: 1166.275 ppm/cm: 9.27286

Compound 3aa

SpinWorks 4: IVA 1219 1H CDCl3

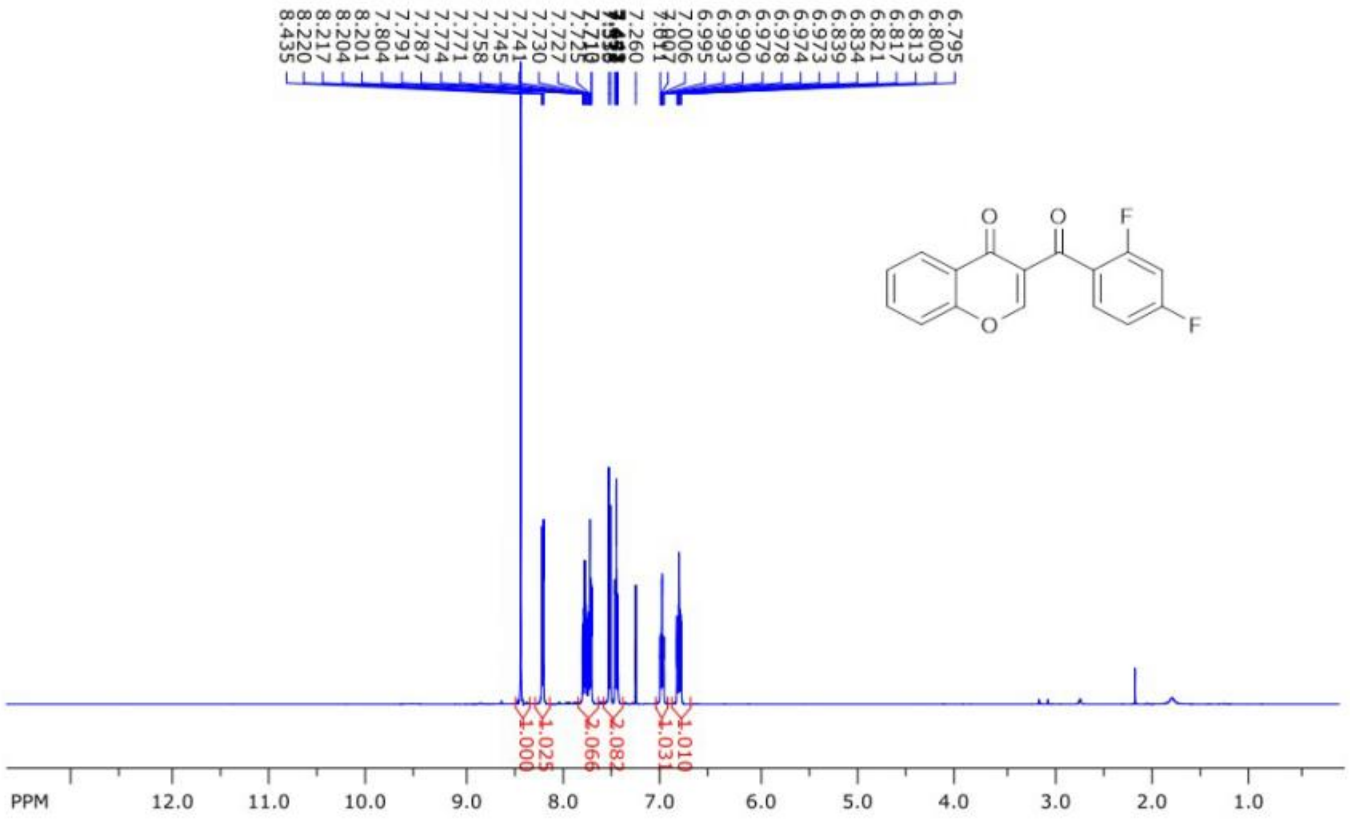

file: ...etones\Fids part 1\IVA 1219\15\fid expt: <zg30>
transmitter freq.: 500.133001 MHz
time domain size: 65536 points
width: 12335.53 Hz = 24.6645 ppm = 0.188225 Hz/pt
number of scans: 32

freq. of 0 ppm: 500.130023 MHz
processed size: 65536 complex points
LB: 0.300 GF: 0.0000
Hz/cm: 272.420 ppm/cm: 0.54469

Compound 3aa

SpinWorks 4: IVA 1216 13C CDCL3

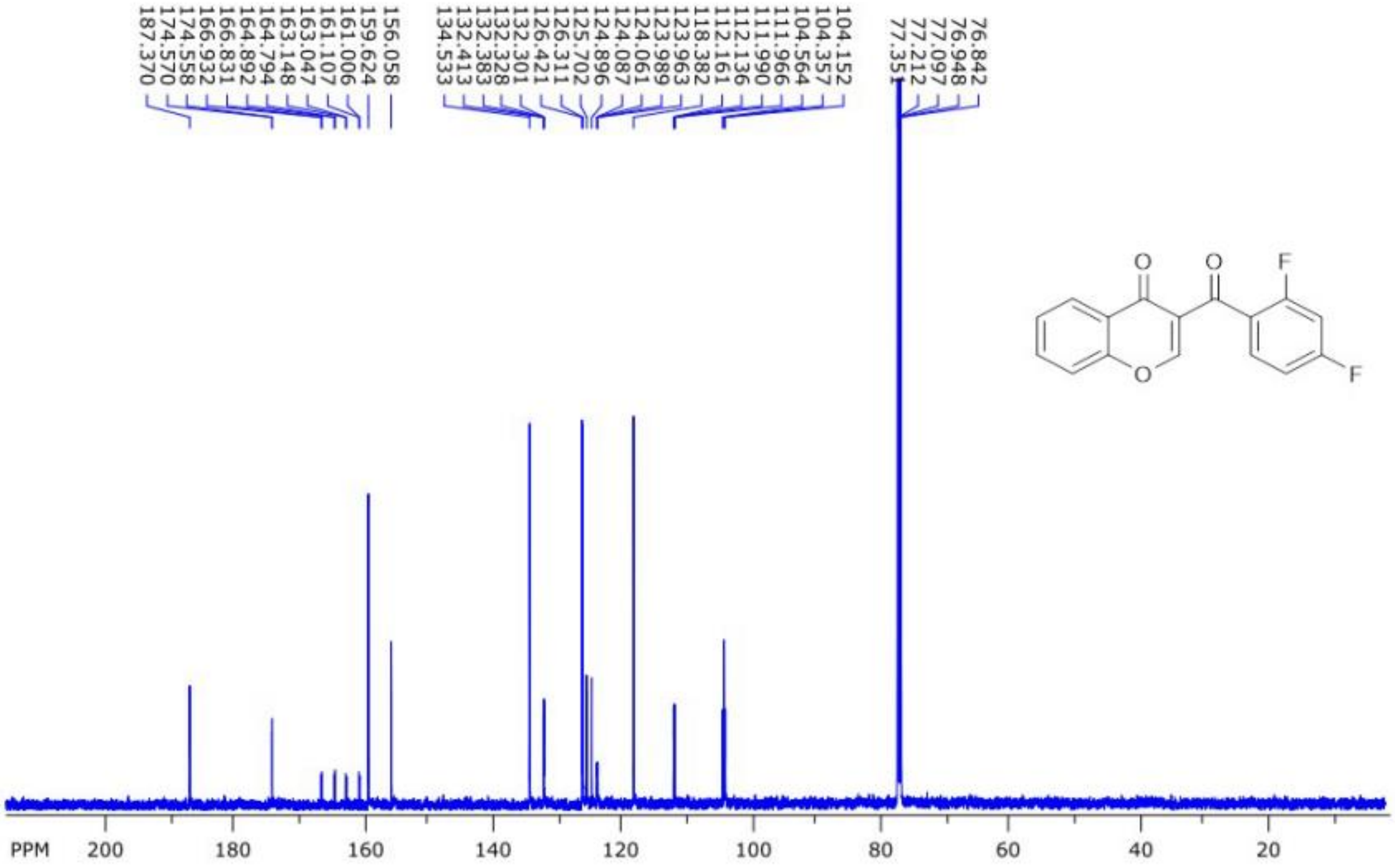

file: ...etones\Fids part 1\IVA 1219\16\fid expt: <zgpg30>
transmitter freq.: 125.772879 MHz
time domain size: 65536 points
width: 36057.69 Hz = 286.6889 ppm = 0.550197 Hz/pt
number of scans: 512

freq. of 0 ppm: 125.757789 MHz
processed size: 32768 complex points
LB: 2.000 GF: 0.0000
Hz/cm: 1078.118 ppm/cm: 8.57194

Compound 7a

SpinWorks 4: IVA 1984 1H DMSO

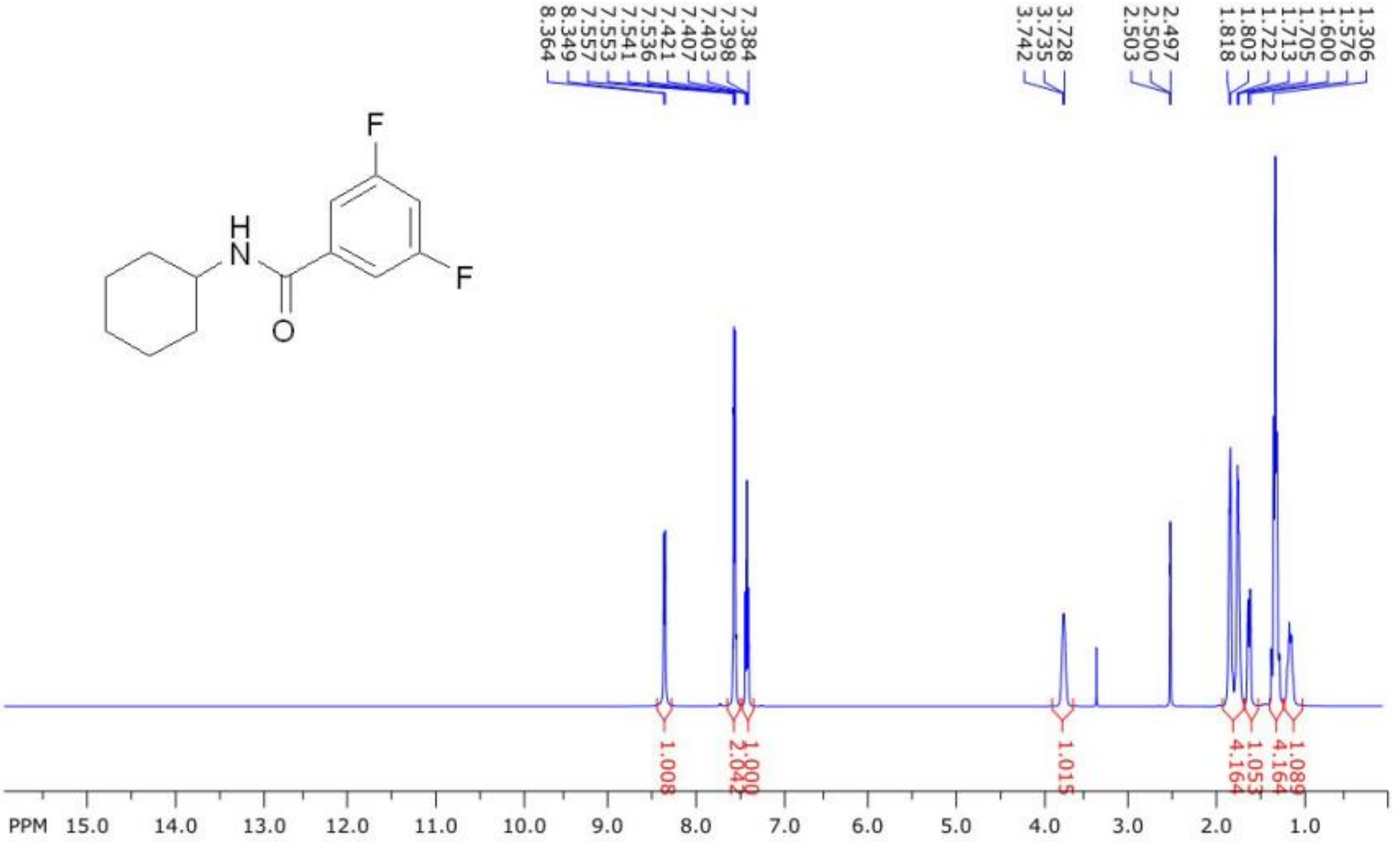

file: D:\NAPO\NMR\500-2\mkr11903\15\fid expt: <zg30>
transmitter freq.: 500.133001 MHz
time domain size: 65536 points
width: 12335.53 Hz = 24.6645 ppm = 0.188225 Hz/pt
number of scans: 24

freq. of 0 ppm: 500.130005 MHz
processed size: 65536 complex points
LB: 0.300 GF: 0.0000
Hz/cm: 319.305 ppm/cm: 0.63844

Compound 7a

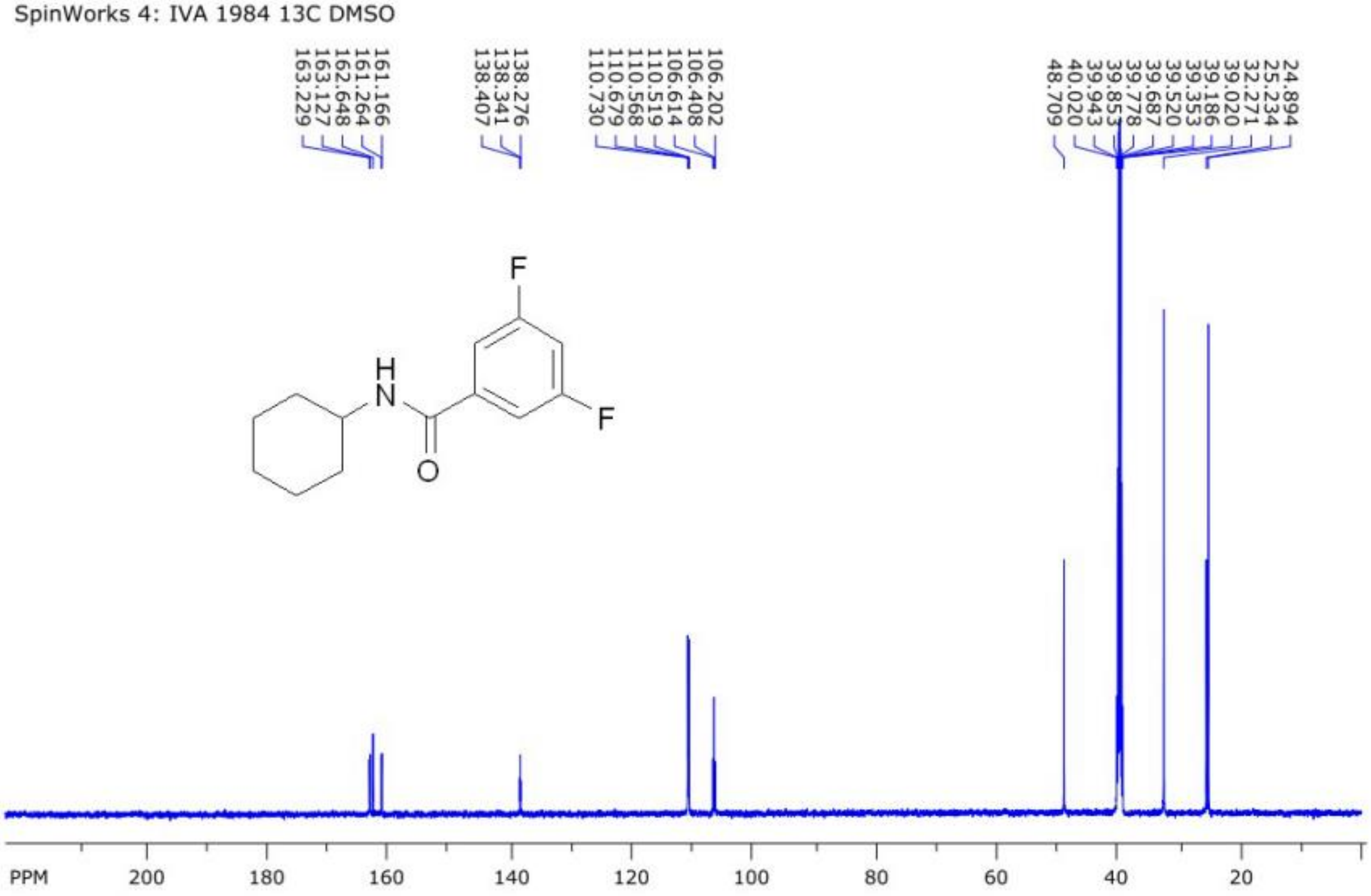

file: D:\NAPO\NMR\500-2\mkr11903\16\fid expt: <zgpg30>
transmitter freq.: 125.772879 MHz
time domain size: 65536 points
width: 36057.69 Hz = 286.6889 ppm = 0.550197 Hz/pt
number of scans: 512

freq. of 0 ppm: 125.757841 MHz
processed size: 32768 complex points
LB: 2.000 GF: 0.0000
Hz/cm: 1124.809 ppm/cm: 8.94317

Compound 7b

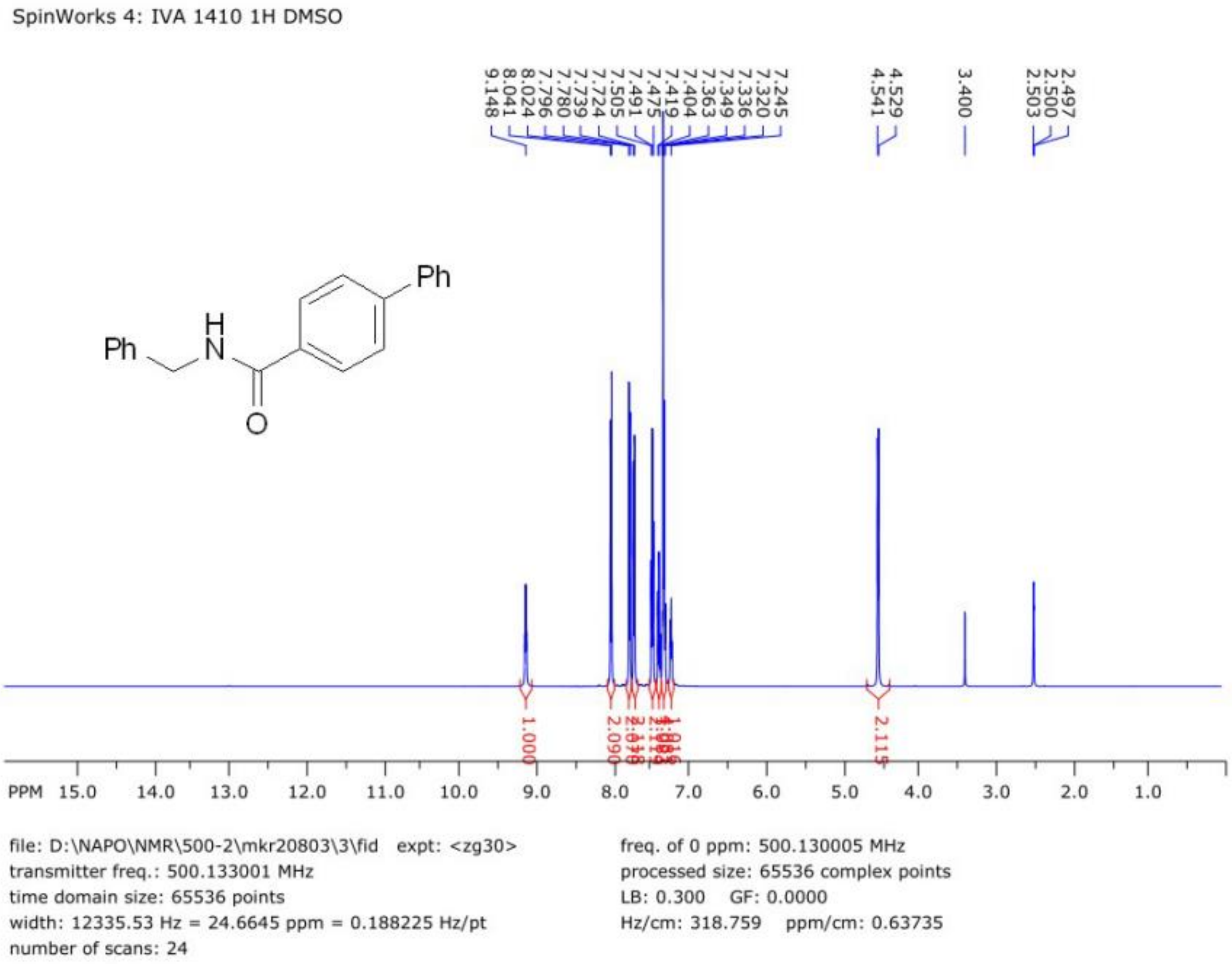
SpinWorks 4: IVA 1410 1H DMSO
9.148
8.041
8.024
7.796
7.780
7.739
7.724
7.505
7.491
7.475
7.419
7.404
7.363
7.349
7.336
7.320
7.245
4.541
4.529
3.400
2.503
2.500
2.497
Ph
H
N
O
Ph
1.000
2.090
2.115
PPM 15.0 14.0 13.0 12.0 11.0 10.0 9.0 8.0 7.0 6.0 5.0 4.0 3.0 2.0 1.0
file: D:\NAPO\NMR\500-2\mkr20803\3\fid expt: <zg30>
transmitter freq.: 500.133001 MHz
time domain size: 65536 points
width: 12335.53 Hz = 24.6645 ppm = 0.188225 Hz/pt
number of scans: 24
freq. of 0 ppm: 500.130005 MHz
processed size: 65536 complex points
LB: 0.300 GF: 0.0000
Hz/cm: 318.759 ppm/cm: 0.63735

Compound 7b

SpinWorks 4: IVA 1410 13C DMSO

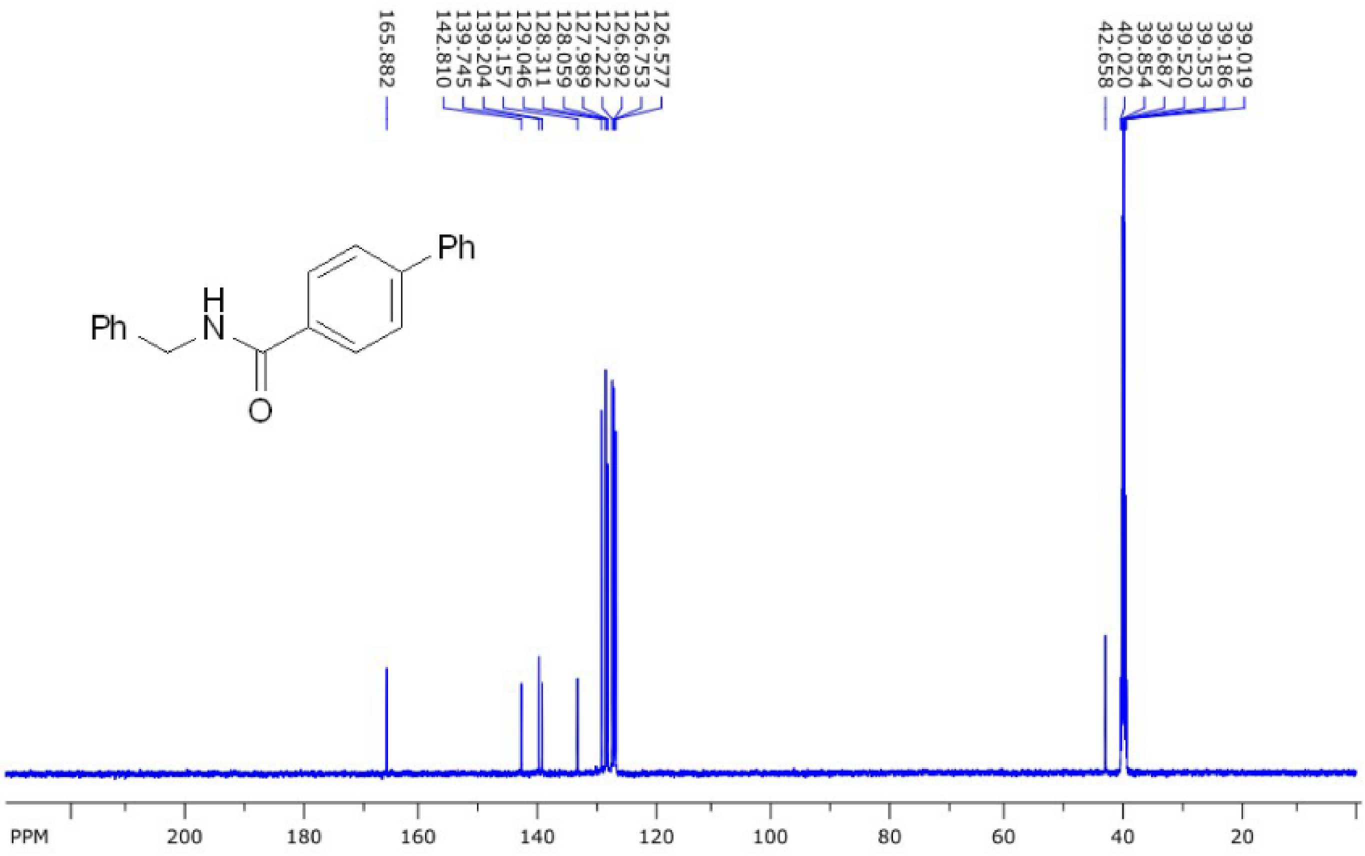

file: D:\NAPO\NMR\500-2\mkr20803\4\fid expt: <zgpg30>
transmitter freq.: 125.772879 MHz
time domain size: 65536 points
width: 36057.69 Hz = 286.6889 ppm = 0.550197 Hz/pt
number of scans: 512

freq. of 0 ppm: 125.757844 MHz
processed size: 32768 complex points
LB: 2.000 GF: 0.0000
Hz/cm: 1164.695 ppm/cm: 9.26031

Compound 7c

SpinWorks 4: IVA 1815 1H CDCl3

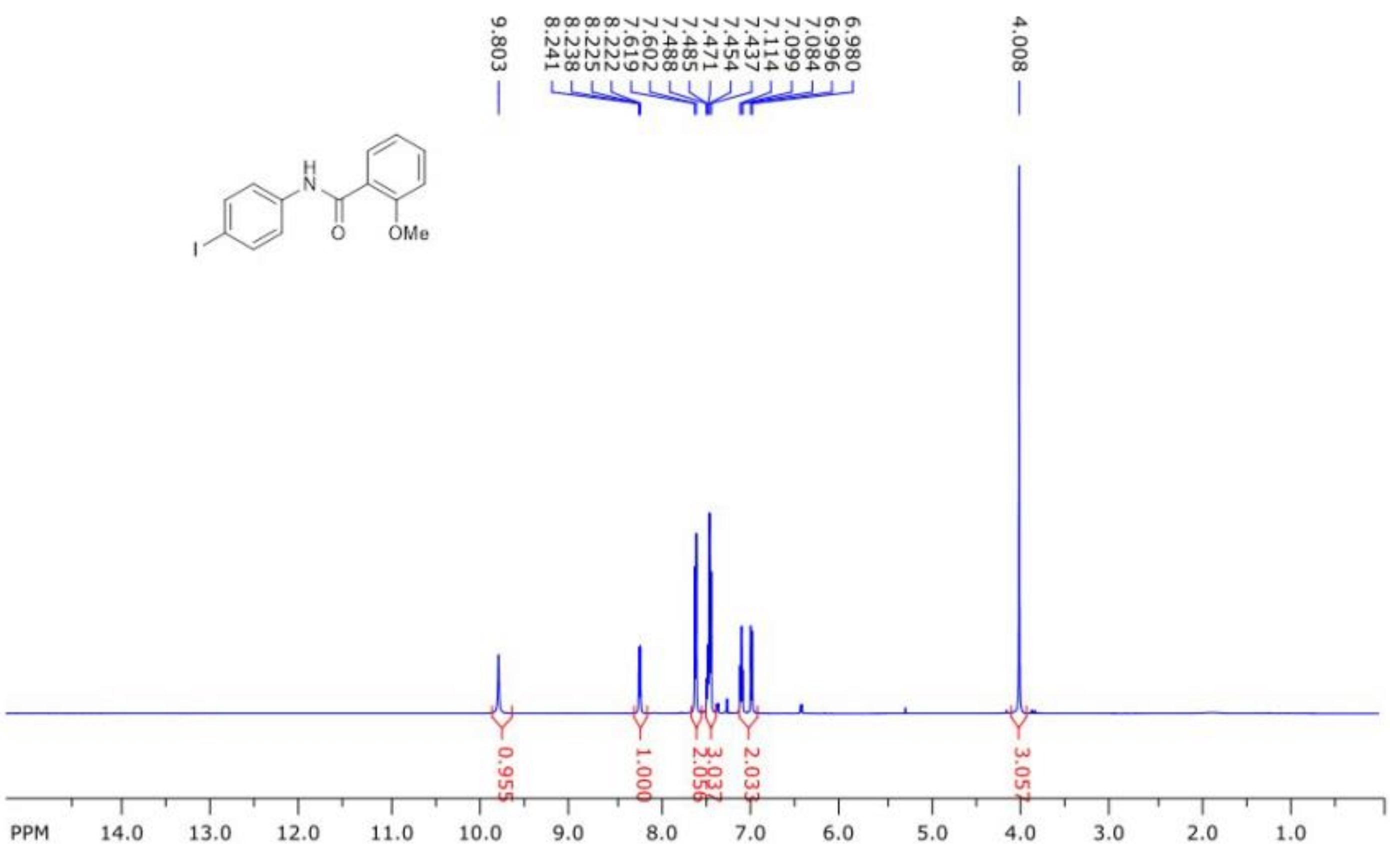

file: ...APO\NMR\500-2\mkr11706\21 1815\fid expt: <zg30>
transmitter freq.: 500.133001 MHz
time domain size: 65536 points
width: 12335.53 Hz = 24.6645 ppm = 0.188225 Hz/pt
number of scans: 24

freq. of 0 ppm: 500.130024 MHz
processed size: 65536 complex points
LB: 0.300 GF: 0.0000
Hz/cm: 305.659 ppm/cm: 0.61116

Compound 7c

SpinWorks 4: IVA 1815 13C CDCl3

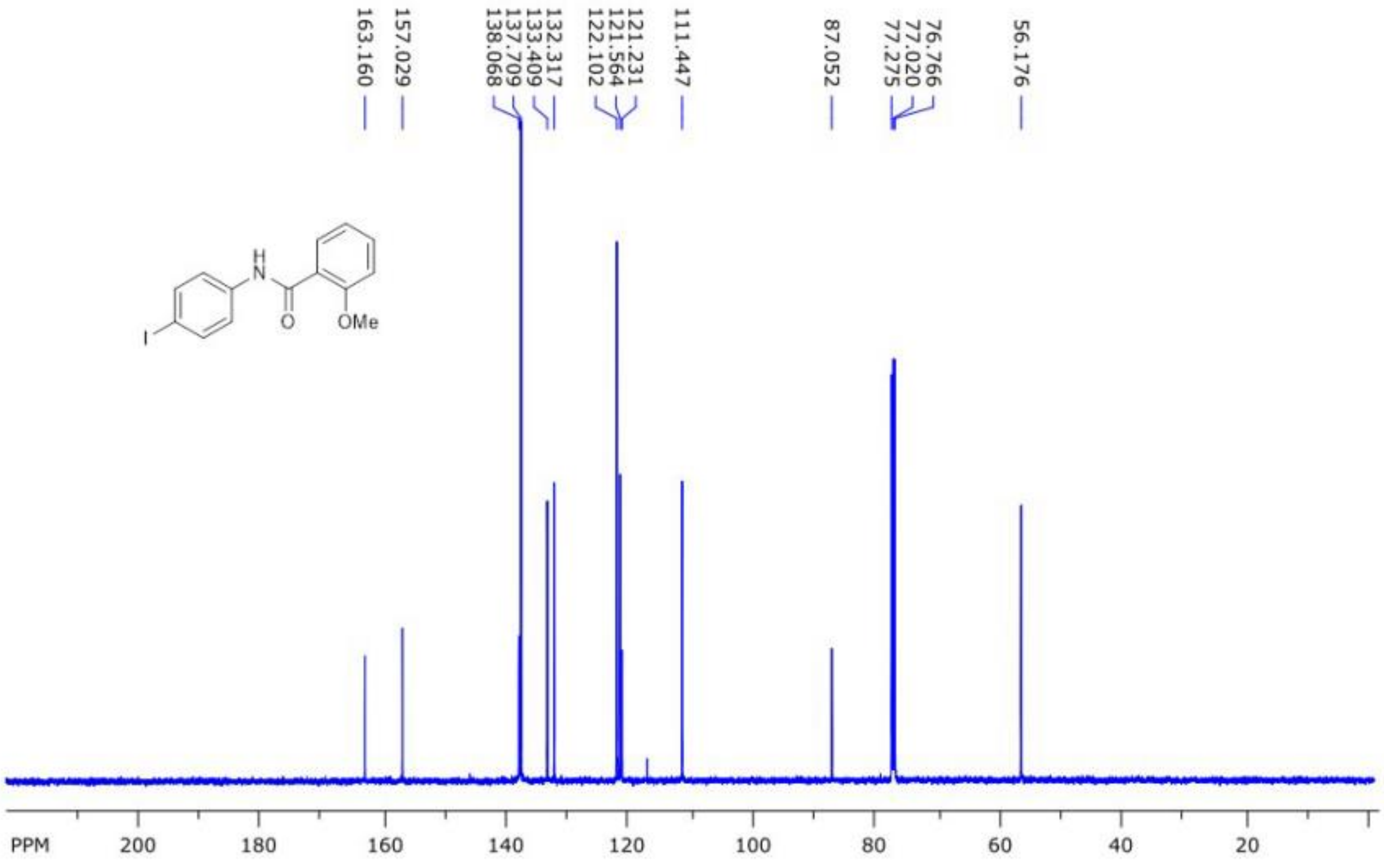

file: D:\NAPO\NMR\500-2\mkr11706\22\fid expt: <zgpg30>
transmitter freq.: 125.772879 MHz
time domain size: 65536 points
width: 36057.69 Hz = 286.6889 ppm = 0.550197 Hz/pt
number of scans: 512

freq. of 0 ppm: 125.757807 MHz
processed size: 32768 complex points
LB: 2.000 GF: 0.0000
Hz/cm: 1121.618 ppm/cm: 8.91780

Compound 7d

SpinWorks 4: IVA 2845 1H DMSO

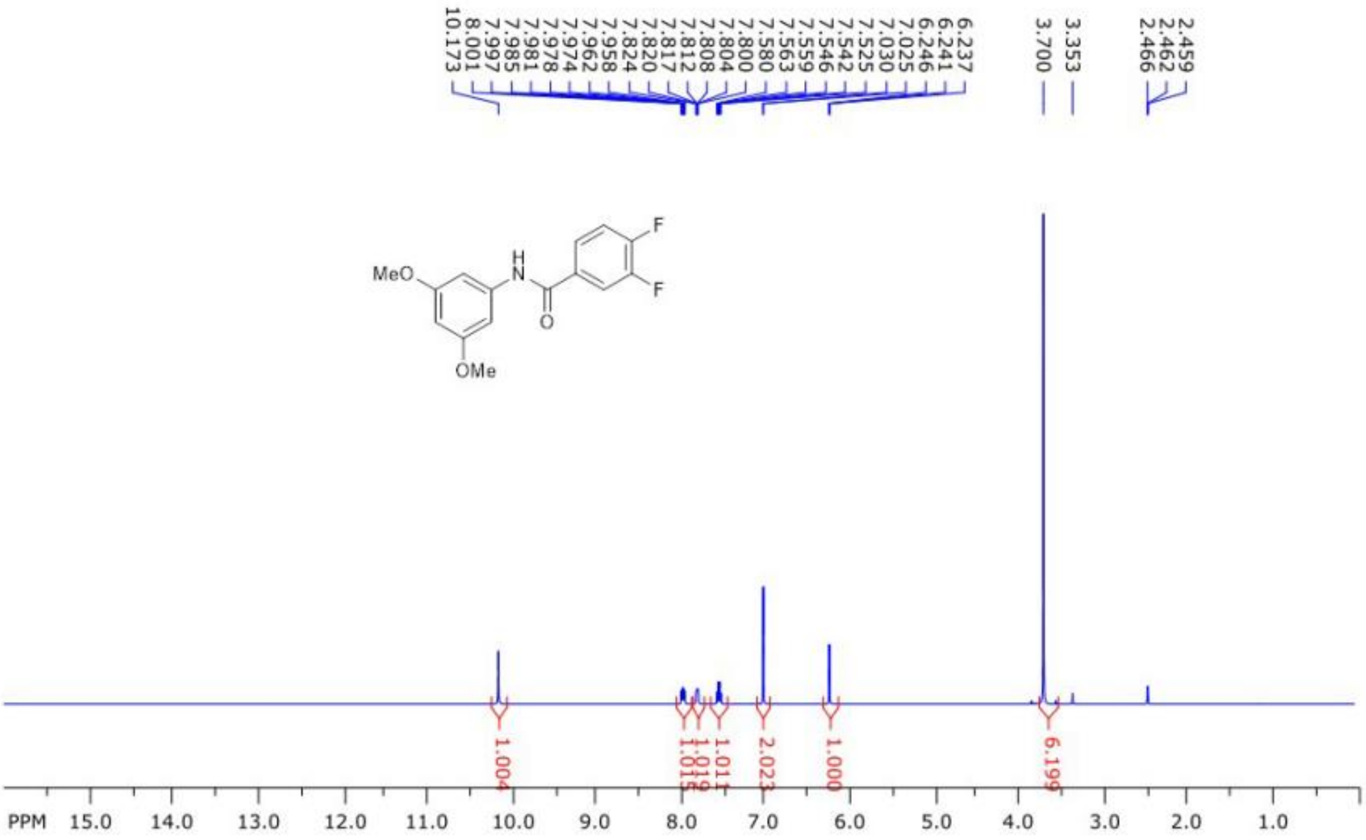

file: ...APO\NMR\500-2\mkr12207\19 2845\fid expt: <zg30>
transmitter freq.: 500.133001 MHz
time domain size: 65536 points
width: 12335.53 Hz = 24.6645 ppm = 0.188225 Hz/pt
number of scans: 24

freq. of 0 ppm: 500.130024 MHz
processed size: 65536 complex points
LB: 0.300 GF: 0.0000
Hz/cm: 320.942 ppm/cm: 0.64171

Compound 7d

SpinWorks 4: IVA 2845 13C DMSO

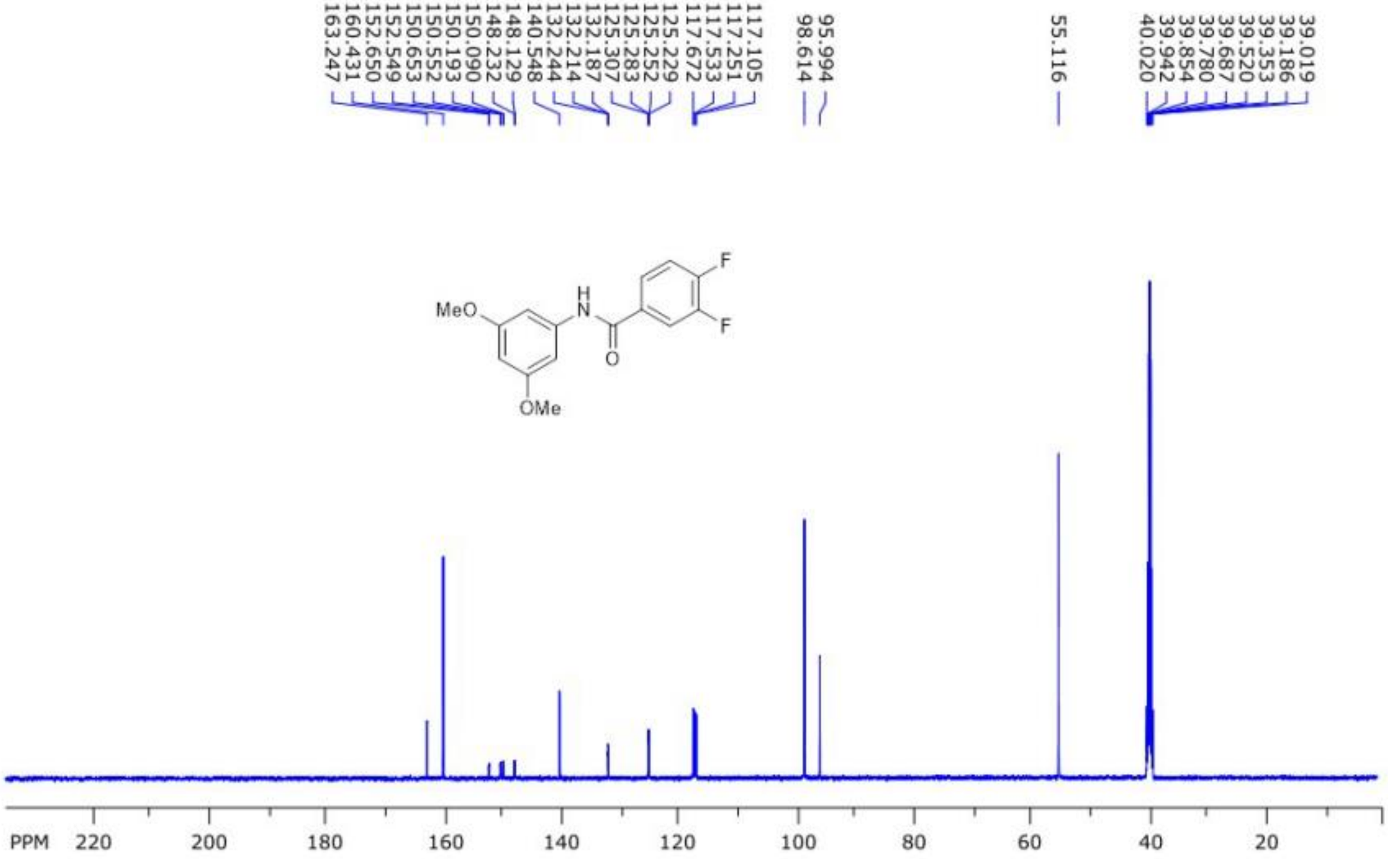

file: D:\NAPO\NMR\500-2\mkr12207\20\fid expt: <zgpg30>
transmitter freq.: 125.772879 MHz
time domain size: 65536 points
width: 36057.69 Hz = 286.6889 ppm = 0.550197 Hz/pt
number of scans: 512

freq. of 0 ppm: 125.757839 MHz
processed size: 32768 complex points
LB: 2.000 GF: 0.0000
Hz/cm: 1180.650 ppm/cm: 9.38716

Compound 7e

SpinWorks 4: SVS 330 1H CDCl3

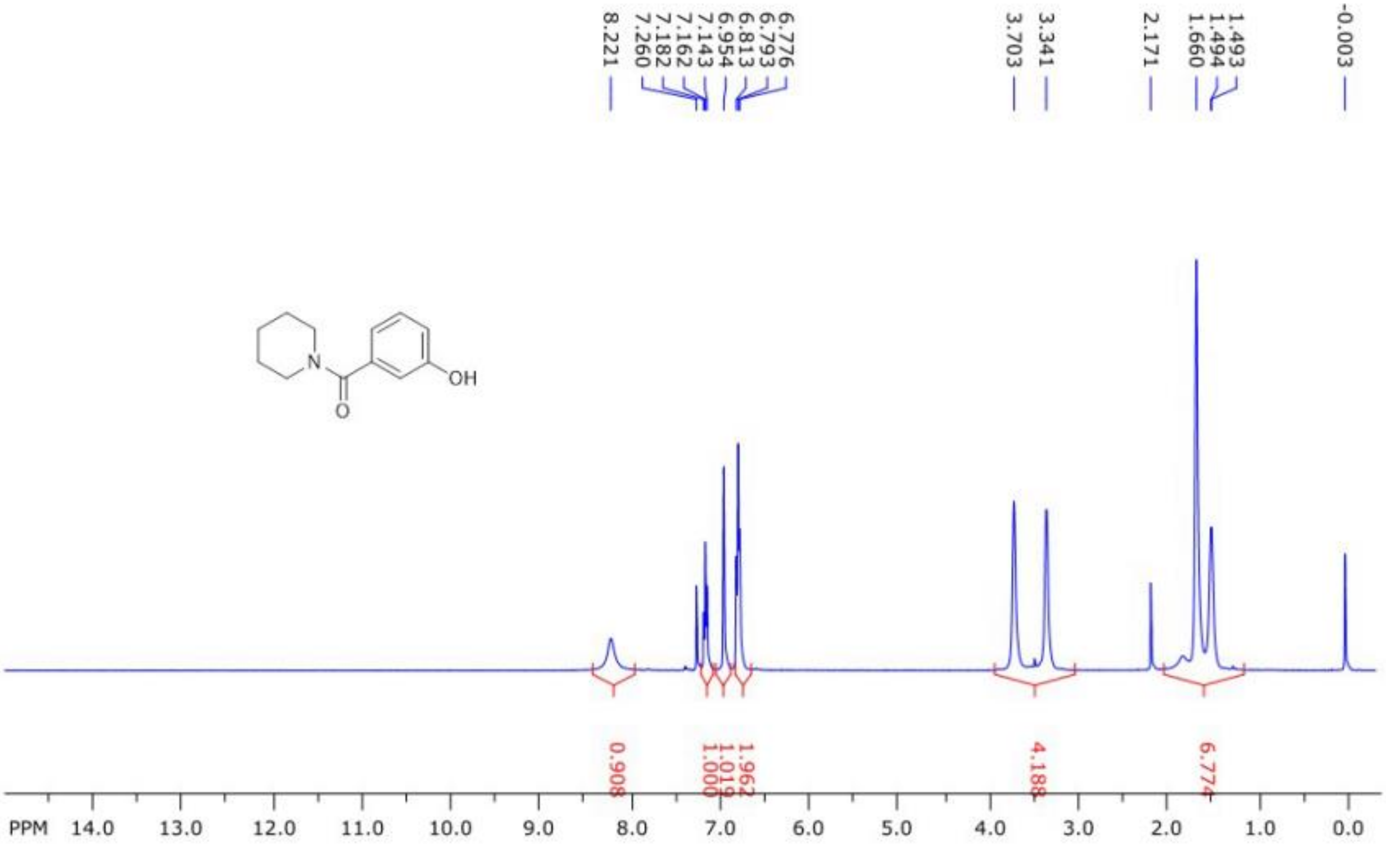

file: D:\NAPO\NMR\JELA\nmr\jn-330\2\fid expt: <zg30>
transmitter freq.: 400.132471 MHz
time domain size: 65536 points
width: 8196.72 Hz = 20.4850 ppm = 0.125072 Hz/pt
number of scans: 16

freq. of 0 ppm: 400.130009 MHz
processed size: 65536 complex points
LB: 0.300 GF: 0.0000
Hz/cm: 245.902 ppm/cm: 0.61455

Compound 7e

SpinWorks 4: SVS 330 13C CDCl3

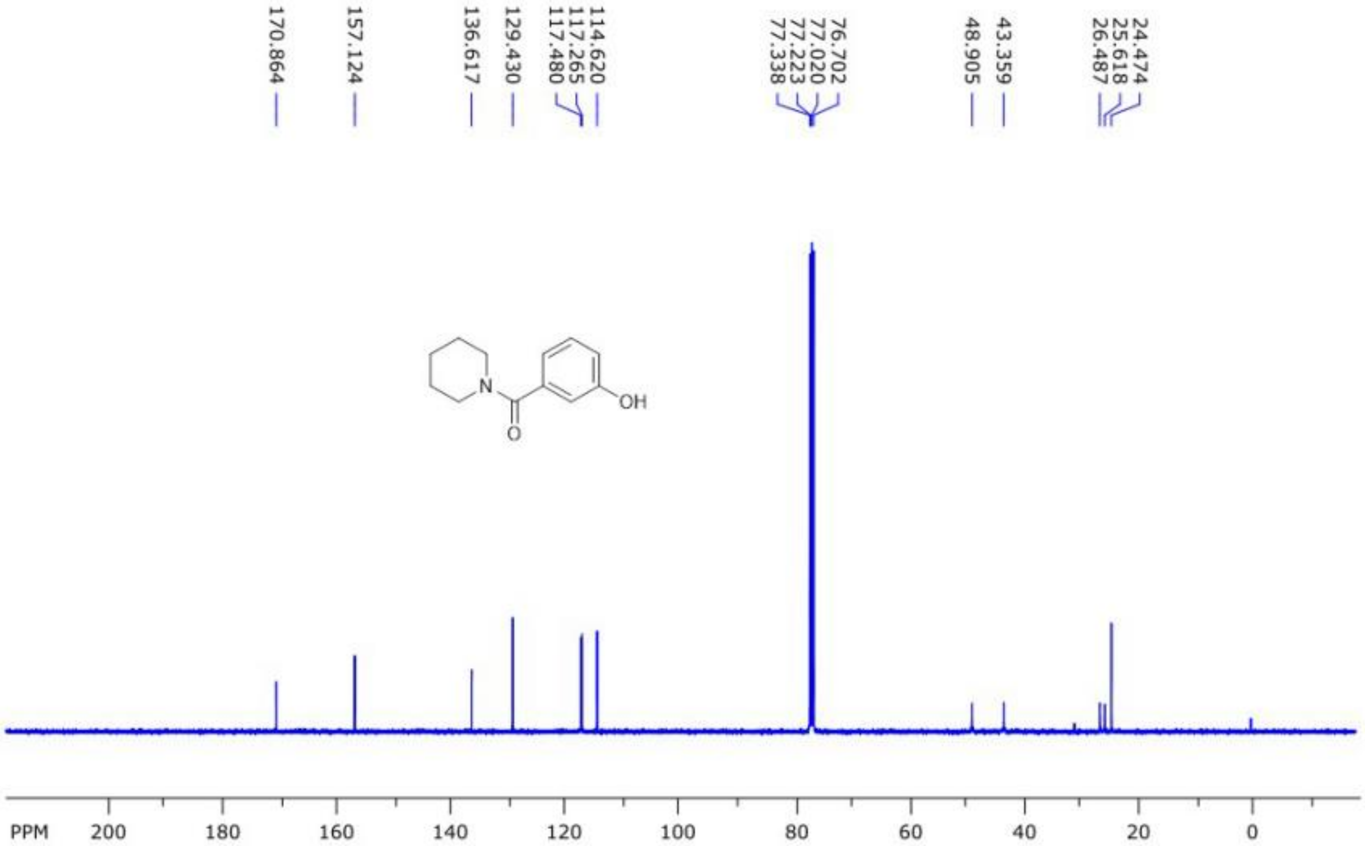

file: D:\NAPO\NMR\JELA\nmr\jn-330\1\fid expt: <zgpg30>
transmitter freq.: 100.622830 MHz
time domain size: 65536 points
width: 23809.52 Hz = 236.6215 ppm = 0.363305 Hz/pt
number of scans: 1024

freq. of 0 ppm: 100.612768 MHz
processed size: 32768 complex points
LB: 1.000 GF: 0.0000
Hz/cm: 952.381 ppm/cm: 9.46486

Compound 8

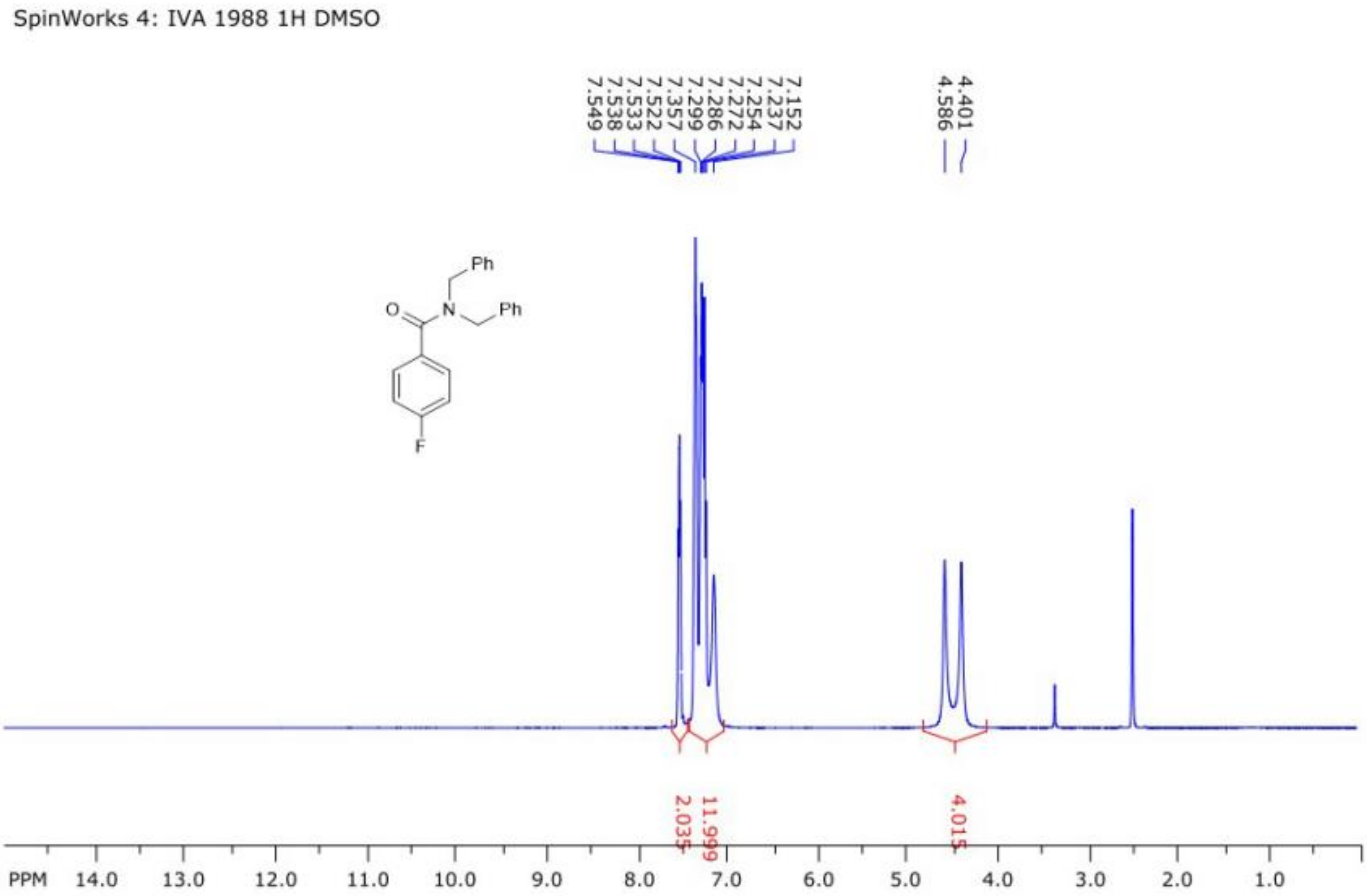

file: D:\NAPO\NMR\500-2\mkr11802\17\fid expt: <zg30>
transmitter freq.: 500.133001 MHz
time domain size: 65536 points
width: 12335.53 Hz = 24.6645 ppm = 0.188225 Hz/pt
number of scans: 24

freq. of 0 ppm: 500.130004 MHz
processed size: 65536 complex points
LB: 0.300 GF: 0.0000
Hz/cm: 300.601 ppm/cm: 0.60104

Compound 8

SpinWorks 4: IVA 1988 13C DMSO

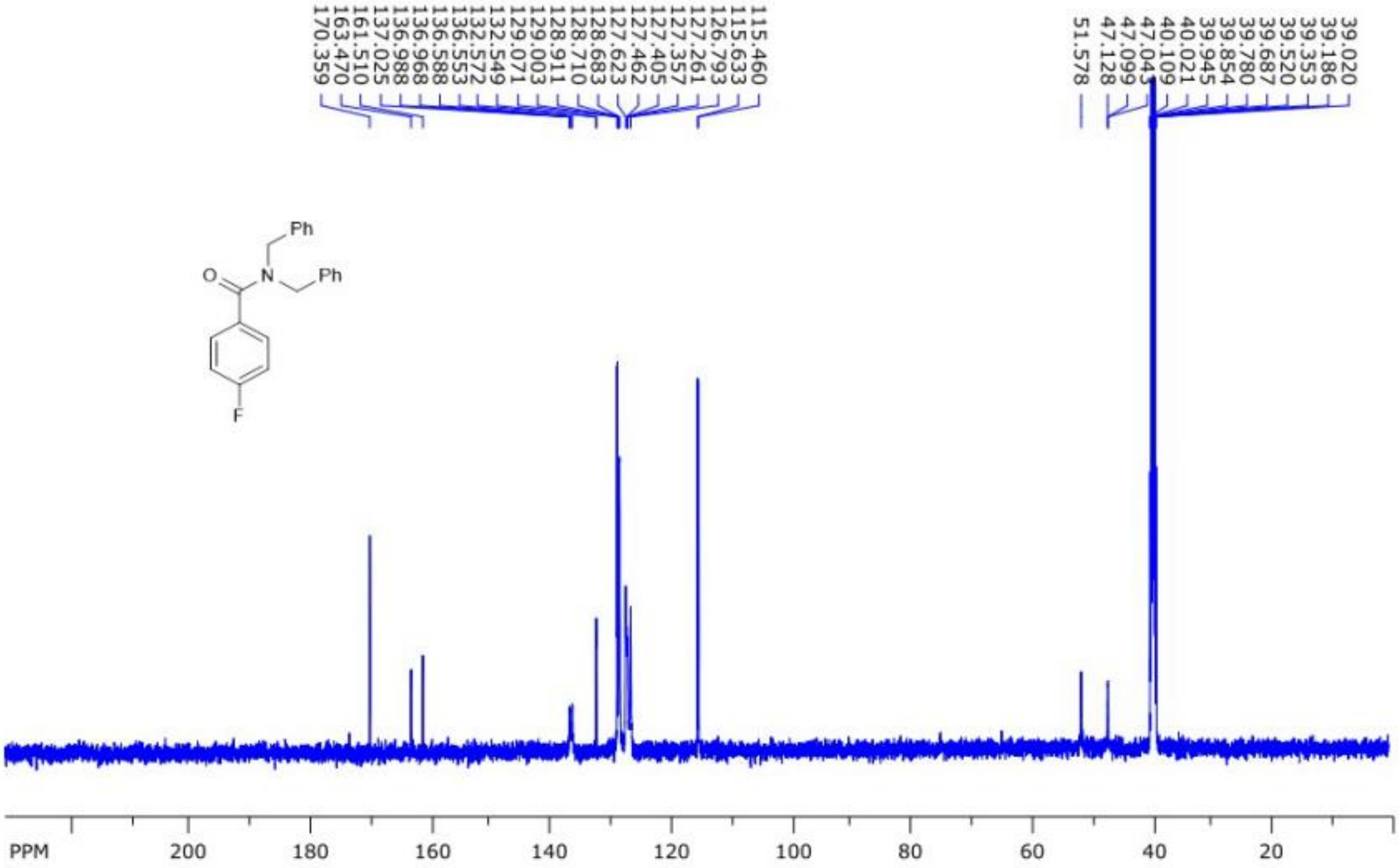

file: D:\NAPO\NMR\500-2\mkr11802\18\fid expt: <zgpg30>
transmitter freq.: 125.772879 MHz
time domain size: 65536 points
width: 36057.69 Hz = 286.6889 ppm = 0.550197 Hz/pt
number of scans: 512

freq. of 0 ppm: 125.757845 MHz
processed size: 32768 complex points
LB: 2.000 GF: 0.0000
Hz/cm: 1161.939 ppm/cm: 9.23839
